# Osteoporose – Definition, Risikoerfassung, Diagnose, Prävention und Therapie (Update 2024): Leitlinie der Österreichischen Gesellschaft für Knochen- und Mineralstoffwechsel

**DOI:** 10.1007/s00508-024-02441-2

**Published:** 2024-10-02

**Authors:** Hans Peter Dimai, Christian Muschitz, Karin Amrein, Rosemarie Bauer, Daniel Cejka, Rudolf Wolfgang Gasser, Reinhard Gruber, Judith Haschka, Timothy Hasenöhrl, Franz Kainberger, Katharina Kerschan-Schindl, Roland Kocijan, Jürgen König, Norbert Kroißenbrunner, Ulrike Kuchler, Christine Oberforcher, Johannes Ott, Georg Pfeiler, Peter Pietschmann, Paul Puchwein, Alexander Schmidt-Ilsinger, Ralf Harun Zwick, Astrid Fahrleitner-Pammer

**Affiliations:** 1 Klinische Abteilung für Endokrinologie und Diabetologie, Universitätsklinik für Innere Medizin, Medizinische Universität Graz, Graz, Österreich; 2 healthPi Medical Center, Medizinische Universität Wien, Wollzeile 1–3, 1010 Wien, Österreich; 3 Dachverband der Sozialversicherungsträger, Wien, Österreich; 4 Interne 3 – Nieren- und Hochdruckerkrankungen, Transplantationsmedizin, Rheumatologie, Ordensklinikum Linz Elisabethinen, Linz, Österreich; 5 Universitätsklinik für Innere Medizin, Medizinische Universität Innsbruck, Innsbruck, Österreich; 6 Universitätszahnklinik, Medizinische Universität Wien, Wien, Österreich; 7 Hanusch Krankenhaus Wien, 1. Medizinische Abteilung, Ludwig Boltzmann Institut für Osteologie, Wien, Österreich; 8 Rheuma-Zentrum Wien-Oberlaa, Wien, Österreich; 9 Universitätsklinik für Physikalische Medizin, Rehabilitation und Arbeitsmedizin, Medizinische Universität Wien, Wien, Österreich; 10 Klinische Abteilung für Biomedizinische Bildgebung und Bildgeführte Therapie, Universitätsklinik für Radiologie und Nuklearmedizin, Medizinische Universität Wien, Wien, Österreich; 11 Department für Ernährungswissenschaften, Universität Wien, Wien, Österreich; 12 Hochschwabpraxis, Turnau, Österreich; 13 Osteoporose Selbsthilfe Österreich, Bregenz, Österreich; 14 Klinische Abteilung für gynäkologische Endokrinologie und Reproduktionsmedizin, Universitätsklinik für Frauenheilkunde, Medizinische Universität Wien, Wien, Österreich; 15 Klinische Abteilung für Gynäkologie und Gynäkologische Onkologie, Universitätsklinik für Frauenheilkunde, Medizinische Universität Wien, Wien, Österreich; 16 Institut für Pathophysiologie und Allergieforschung, Zentrum für Pathophysiologie, Infektiologie und Immunologie (CEPII), Medizinische Universität Wien, Wien, Österreich; 17 Universitätsklinik für Orthopädie und Traumatologie, Medizinische Universität Graz, Graz, Österreich; 18 Österreichische Apothekerkammer, Wien, Österreich; 19 Ludwig Boltzmann Institut für Rehabilitation Research, Therme Wien Med, Wien, Österreich; 20 Privatordination Prof. Dr. Astrid Fahrleitner-Pammer, http://www.knochenwelt.at; 21 Klinische Abteilung für Endokrinologie und Diabetes, Universitätsklinik für Innere Medizin, Medizinische Universität Graz, Graz, Österreich; 22 Medizinische Universität Wien, Währinger Gürtel 18–20, 1090 Wien, Österreich

**Keywords:** Osteoporotische Fraktur, Frakturwahrscheinlichkeit, FRAX, Interventionsschwellen, Monitoring, Osteoporotic fracture, Fracture probability, FRAX, Intervention threshold, Monitoring

## Abstract

**Hintergrund:**

Österreich zählt zu den Ländern mit der höchsten Inzidenz und Prävalenz osteoporotischer Frakturen weltweit. Leitlinien zur Prävention und zum Management der Osteoporose wurden erstmals im Jahr 2010 unter der Schirmherrschaft des damaligen Hauptverbandes der Österreichischen Sozialversicherungsträger veröffentlicht und im Jahr 2017 aktualisiert. Die vorliegende umfassend aktualisierte Leitlinie der Österreichischen Gesellschaft für Knochen- und Mineralstoffwechsel (ÖGKM) richtet sich an Ärztinnen und Ärzte aller Fachrichtungen sowie an Entscheidungsträger und Institutionen im österreichischen Gesundheitssystem. Ziel dieser Leitlinie ist es, die Qualität der medizinischen Versorgung von Patienten mit Osteoporose und osteoporotischen Frakturen in Österreich zu stärken und zu verbessern.

**Methoden:**

Evidenzbasierte Empfehlungen wurden unter Berücksichtigung randomisierter kontrollierter Studien, systematischer Reviews und Metaanalysen sowie europäischer und internationaler Quellleitlinien zur Osteoporose, welche bis zum 1. Juni 2023 veröffentlicht wurden, erstellt. Die verwendeten Empfehlungsstärken („bedingt“ und „stark“) basieren auf der Stärke der jeweiligen Evidenzgrade. Letztere orientieren sich an den SIGN-Kriterien (1++ bis 3), welche in NOGG-Kriterien (Ia bis IV) transformiert wurden.

**Ergebnisse:**

Die Leitlinie umfasst alle Aspekte im Zusammenhang mit Osteoporose und osteoporotischen Frakturen, einschließlich sekundärer Ursachen, Prävention, Diagnose, Erfassung der 10-Jahres-Frakturwahrscheinlichkeit mittels FRAX®, Ermittlung FRAX®-basierter Österreich-spezifischer Interventionsschwellen, medikamentöser und nichtmedikamentöser Therapieoptionen sowie Möglichkeiten des Therapiemonitorings. Empfehlungen für den niedergelassenen Bereich und Entscheidungsträger und Institutionen im österreichischen Gesundheitssystem berücksichtigen strukturierte Versorgungsmodelle sowie Möglichkeiten zur gezielten Vorsorge.

**Schlussfolgerung:**

Die vorliegende Leitlinie stellt umfassende, evidenzbasierte Informationen sowie Handlungsanleitungen zum Krankheitsbild der Osteoporose zur Verfügung. Es ist davon auszugehen, dass die Qualität der Versorgung von Personen mit diesem Krankheitsbild in allen Ebenen des österreichischen Gesundheitswesens entscheidend verbessert werden kann.

## Abstract (en)

**Background:**

Austria is among the countries with the highest incidence and prevalence of osteoporotic fractures worldwide. Guidelines for the prevention and management of osteoporosis were first published in 2010 under the auspices of the then Federation of Austrian Social Security Institutions and updated in 2017. The present comprehensively updated guidelines of the Austrian Society for Bone and Mineral Research are aimed at physicians of all specialties as well as decision makers and institutions in the Austrian healthcare system. The aim of these guidelines is to strengthen and improve the quality of medical care of patients with osteoporosis and osteoporotic fractures in Austria.

**Methods:**

These evidence-based recommendations were compiled taking randomized controlled trials, systematic reviews and meta-analyses as well as European and international reference guidelines published before 1 June 2023 into consideration. The grading of recommendations used (“conditional” and “strong”) are based on the strength of the evidence. The evidence levels used mutual conversions of SIGN (1++ to 3) to NOGG criteria (Ia to IV).

**Results:**

The guidelines include all aspects associated with osteoporosis and osteoporotic fractures, such as secondary causes, prevention, diagnosis, estimation of the 10-year fracture risk using FRAX®, determination of Austria-specific FRAX®-based intervention thresholds, drug-based and non-drug-based treatment options and treatment monitoring. Recommendations for the office-based setting and decision makers and institutions in the Austrian healthcare system consider structured care models and options for osteoporosis-specific screening.

**Conclusion:**

The guidelines present comprehensive, evidence-based information and instructions for the treatment of osteoporosis. It is expected that the quality of medical care for patients with this clinical picture will be substantially improved at all levels of the Austrian healthcare system.

## 1 Einführung

### 1.1 Zielgruppen, Ziele und Hintergrund der Leitlinie

Diese österreichische Leitlinie richtet sich an Ärztinnen und Ärzte für Allgemeinmedizin sowie an Fachärztinnen und Fachärzte aller Richtungen, um eine wissenschaftlich fundierte und dennoch praxisnahe Orientierungshilfe in der Prävention und Behandlung der Erkrankung Osteoporose und osteoporotischer Frakturen zur Verfügung zu stellen. Darüber hinaus soll sie allen Entscheidungsträgern und Institutionen im österreichischen Gesundheitssystem als zuverlässiges Nachschlagwerk im Kontext der Osteoporose und osteoporotischer Frakturen dienen und somit die Qualität der medizinischen Versorgung von Patienten mit Osteoporose in Österreich stärken und verbessern. Präventiver und/oder therapeutischer Nutzen für die Patienten stehen klar im Vordergrund dieser Leitlinien, während ökonomische Aspekte einschließlich Aspekten der Erstattungsökonomie keine Berücksichtigung finden.

Die in dieser Leitlinie konsensuell erstellten Empfehlungen basieren auf den jeweiligen Evidenzgraden, welche sich aus der zur Verfügung stehenden wissenschaftlichen Literatur zu einem definierten Thema bis zu einem definierten Stichtag ableiten lassen (s. Appendix B „Evidenzgrade und Empfehlungsstärken“). Dabei orientiert sich die vorliegende Leitlinie entsprechend einer Empfehlung des Europarates an bereits zur Verfügung stehenden nationalen und internationalen (rezenten) Leitlinien, welche unter Einhaltung aller maßgeblichen qualitativen Kriterien Evidenzgrade und Empfehlungsstärken beinhalten (s. Appendix C „Methodik der Leitlinienrecherche“).

### 1.2 Definition der Osteoporose

Empfehlungen:

- Entsprechend einer WHO-Empfehlung kann eine Osteoporose bei Männern ab dem 50. Lebensjahr und postmenopausalen Frauen *im Sinne der operationalen Definition* dann diagnostiziert werden, wenn die mittels 2‑Spektren-Röntgenabsorptiometrie (i. e. DXA) erfasste KMD 2,5 SD unter dem mittleren Normwert gesunder junger Erwachsener liegt (i. e., T‑Score ≤ −2,5); *bedingte Empfehlung*.
- Der von der WHO empfohlene diagnostische Schwellenwert (T-Score ≤ −2,5) sollte nicht als therapeutischer Schwellenwert angewandt werden; *starke Empfehlung*.
- Eine Osteoporose sollte jedenfalls diagnostiziert werden, wenn bei nur osteopenisch verminderter oder normaler KMD eine Fraktur unter geringem Trauma auftritt; *starke Empfehlung*.

Die Osteoporose ist eine systemische Skeletterkrankung, welche durch eine verminderte Knochenmasse und eine gestörte Mikroarchitektur des Knochens charakterisiert ist. Diese Veränderungen führen zu einer erhöhten Brüchigkeit des Knochens und folglich zu einem erhöhten Frakturrisiko [1]. Während die Mikroarchitektur mangels geeigneter und verfügbarer Technologien in der täglichen klinischen Praxis nicht erfasst werden kann, lässt sich die Knochenmasse mittels 2‑Spektren-Röntgenabsorptiometrie (engl. „dual X‑ray absorptiometry“ [DXA]) gut quantifizieren. Die operationale Definition der Osteoporose basiert daher entsprechend einer Empfehlung der Weltgesundheitsorganisation (WHO) auf den Ergebnissen einer solchen Messung. Liegt der gemessene Wert (ausgedrückt als T‑Score) 2,5 Standardabweichungen (SD) unter dem mittleren Normwert gesunder junger Erwachsener, so kann die Diagnose einer Osteoporose gestellt werden [1]; *Evidenzgrad IIa*. Das gleichzeitige Vorliegen einer osteoporotisch verminderten Knochenmineraldichte (KMD) und einer prävalenten Fraktur rechtfertigt die Diagnose einer „manifesten Osteoporose“ (s. Kap. 5 „Diagnose“). Die triviale und wenig wissenschaftliche Rationale, einen T‑Score von −2,5 als diagnostische Schwelle bei postmenopausalen Frauen einzuführen, basiert auf Daten aus den 1990er-Jahren, welche gezeigt haben, dass bei diesem Wert ungefähr 30 % aller postmenopausalen Frauen eine Osteoporose aufweisen. Diese von der WHO gestützten diagnostischen Kategorien haben grundsätzlich auch heute noch ihre Gültigkeit, dürfen jedoch keinesfalls mit Behandlungsschwellen gleichgesetzt werden. So bedeutet zum Beispiel ein T‑Score von −2,5 bei einer jungen postmenopausalen Frau ohne sonstige klinische Risikofaktoren nicht gleichzeitig, dass mit einer Osteoporose-spezifischen Behandlung zu beginnen ist (s. Abschn. 6.3. „Interventionsschwellen“). Eine Frau gleichen Alters mit einem T‑Score von −1,9 und einer rezenten niedrigtraumatischen Wirbelkörperfraktur, einem der stärksten Risikofaktoren für weitere osteoporotische Frakturen, ist hingegen umgehend zu behandeln, da die rezente Wirbelkörperfraktur alleine bereits eine Behandlungsindikation darstellt. Eine manifeste Osteoporose ist jedenfalls auch dann zu diagnostizieren, wenn eine Fraktur bei geringem Trauma und bei lediglich osteopenisch verminderter oder sogar normaler KMD auftritt [2]. Der Vollständigkeit halber sei hier noch erwähnt, dass es seit 1994 nur noch einen einzigen Versuch gab, die ursprüngliche Definition der Osteoporose an den aktuellen Wissensstand anzupassen [3]. Im Rahmen einer National Institutes of Health (NIH) Consensus Development Panel Conference wurde vorgeschlagen, die Osteoporose als Knochenerkrankung zu definieren, welche durch eine eingeschränkte Festigkeit des Knochens charakterisiert ist, wodurch ein erhöhtes Frakturrisiko bedingt ist. Dieser Vorschlag verblieb allerdings mangels globaler Zustimmung durch maßgebliche wissenschaftliche Gesellschaften auf nationaler Ebene.

### 1.3 Definition der osteoporotischen Fraktur

Empfehlungen:

- Tritt eine Fraktur unter Krafteinwirkung eines Sturzes aus Standhöhe oder weniger auf, kann eine osteoporotische Fraktur angenommen werden; *bedingte Empfehlung*.
- Die Mehrheit aller Frakturen tritt bei nicht-osteoporotisch verminderter KMD auf; *starke Empfehlung*.
- Ein osteoporotisch veränderter Knochen bricht unter einem schweren Trauma eher als ein nicht-osteoporotisch veränderter Knochen; *starke Empfehlung*.

Für die osteoporotische Fraktur per se steht bislang keine konsensuell entwickelte Definition zur Verfügung. Begriffe wie „Insuffizienzfraktur“, „osteoporotische Fraktur“, „atraumatische Fraktur“, „niedrigtraumatische Fraktur“ oder „Fragilitätsfraktur“ werden in der Praxis beliebig und wechselweise angewandt. Einer Empfehlung der WHO zufolge kann von einer Osteoporose ausgegangen werden, wenn eine Fraktur unter einem Trauma auftritt, welches einer Krafteinwirkung eines Sturzes aus Standhöhe oder darunter entspricht [4]. Insgesamt ereignet sich die Mehrheit aller Frakturen bei nicht-osteoporotisch verminderter KMD [2, 5]; *Evidenzgrad Ib*. Unabhängig davon sollte bedacht werden, dass ein osteoporotisch veränderter Knochen auch unter einem schweren Trauma eher bricht als ein nicht-osteoporotisch veränderter Knochen [6]. Zu den am häufigsten betroffenen Skelettregionen zählen die Brust- und Lendenwirbelsäule, die Hüfte (proximales Femur), der proximale Humerus sowie der distale Unterarm. Diese typischen osteoporotischen Frakturen werden daher häufig unter dem Begriff der „major osteoporotic fractures“ (MOF; hüftnahe Fraktur, klinisch vertebrale Fraktur, Unterarmfraktur, Humerusfraktur) zusammengefasst [7]. Auch die Beckenfraktur (Schambeinast) ist eine häufige osteoporotische Fraktur.

## 2 Epidemiologie

Im Kontext epidemiologischer Untersuchungen zum Thema Osteoporose ist grundsätzlich zu unterscheiden, ob sich die erhobenen Daten auf die Prävalenz der Osteoporose im Sinne der WHO-Definition, also einen T‑Score von −2,5 oder kleiner (T-Score ≤ −2,5), in der KMD-Messung (mittels DXA-Methode) beziehen oder auf die Prävalenz und Inzidenz von (osteoporotischen) Frakturen.

### 2.1 Prävalenz der Osteoporose im Sinne der WHO-Definition

Daten zur Prävalenz der Osteoporose im Sinne der WHO-Definition sind grundsätzlich bestenfalls grobe Schätzungen, da es weltweit in keinem einzigen Land flächendeckende Erhebungen hierzu gibt. Zumeist handelt es sich um Extrapolationen von lokal oder innerhalb von einzelnen Gesundheitseinrichtungen erhobenen Daten. Für die Mitgliedstaaten der Europäischen Union (EU) wird in einer vergleichenden Untersuchung davon ausgegangen, dass die durchschnittliche KMD der Hüfte von Frauen und Männern der Altersgruppe ≥ 50 Jahre in allen Staaten ähnlich ist [8]. Unter dieser Prämisse ergibt sich für Frauen der Altersgruppe ≥ 50 Jahre in den Ländern der Europäischen Union eine Prävalenz von 19,3 % (Zypern) bis 23,4 % (Italien) und für Männer eine Prävalenz von 5,8 % (Polen) bis 6,9 % (Italien). Österreich liegt hier jeweils im oberen Drittel (Frauen 22,2 %, Männer 6,5 %). Obwohl die KMD ein wichtiger Prädiktor für das Frakturrisiko ist, wird sie aufgrund des sehr unterschiedlichen Frakturrisikos einzelner Staaten nicht als epidemiologisch maßgebliches Kriterium eingestuft [9].

### 2.2 Inzidenz osteoporotischer Frakturen

Daten zur Inzidenz typischer osteoporotischer Frakturen in der Altersgruppe ≥ 50 Jahre stehen weltweit nur sehr spärlich zur Verfügung. Die zuverlässigsten epidemiologischen Daten unter allen (osteoporotischen) Frakturen liegen für die hüftnahe Fraktur vor. Dies ist dem Umstand geschuldet, dass de facto alle hüftnahen Frakturen zu einer stationären Aufnahme führen und die meisten Staaten weltweit die International Classification of Diseases (ICD) zur Diagnose-Codierung im stationären Bereich verwenden. Diese Daten werden zumeist in einem zentralen nationalen Register gesichert und verarbeitet und stehen daher epidemiologischen Fragestellungen zur Verfügung [10]. Wesentlich spärlicher, und dies auch nur für einige wenige Staaten wie etwa Österreich, stehen belastbare Zahlen zur Inzidenz der übrigen MOF zur Verfügung.

### 2.3 Inzidenz von hüftnahen Frakturen

Die weltweite Inzidenz von hüftnahen Frakturen wird für das Jahr 2019 auf etwa 14,2 Mio. (182/100.000) geschätzt [11]. Daten zur alters- und geschlechtsstandardisierten Inzidenz zeigen eine Inzidenz von 95,1/100.000 in Brasilien bis zu 315,9/100.000 in Dänemark [12]. Während in zahlreichen Ländern die Inzidenzzahlen für hüftnahe Frakturen gleich bleiben oder sinken, ist aufgrund des globalen Zuwachses der älteren Bevölkerung mit einem weiteren Anstieg der Zahlen bis 2050 zu rechnen. Ein detaillierterer Vergleich für das Jahr 2010 macht die großen regionalen Unterschiede in der alters- und geschlechtsstandardisierten Inzidenz für hüftnahe Frakturen deutlich. Diese beträgt in Dänemark etwa das Zehnfache der Inzidenz der hüftnahen Frakturen von Südafrika [13].

Österreich zählt global zu den Ländern mit den höchsten Inzidenzraten und wird im EU-Raum nur noch von Schweden und Dänemark übertroffen. Eigene nationale Analysen über einen gesamten Zeitraum von 30 Jahren (1989–2018) zeigen, dass die altersstandardisierte Inzidenz nach einem steilen Anstieg bis zum Jahr 2000 in eine Plateauphase überging, welcher ab dem Jahr 2008 ein deutlicher Abwärtstrend folgte [14]. Erwähnt sei, dass hierbei die Absolutzahlen beim männlichen Geschlecht bis zum Ende des Beobachtungszeitraumes stetig anstiegen. Im Jahr 2010 betrug die altersstandardisierte Inzidenz von hüftnahen Frakturen in Österreich für Frauen 629/100.000 und für Männer 285/100.000.

### 2.4 Inzidenz von distalen Unterarmfrakturen

Ein weltweiter Vergleich der Inzidenz von distalen Unterarmfrakturen steht aus den oben erwähnten Gründen nicht zur Verfügung. Nur einige wenige Staaten, wie etwa Schweden oder Österreich, haben belastbare Daten veröffentlicht, welche sowohl ambulant als auch stationär behandelte Frakturen berücksichtigen. Für Österreich wurde solcherart im Beobachtungszeitraum 1989 bis 2010 für Frauen eine durchschnittliche altersstandardisierte Inzidenz von 658/100.000 und für Männer von 166,5/100.000 errechnet [15], mit einem leichten Abwärtstrend für Frauen über den gesamten Beobachtungszeitraum. Daten aus jüngeren Untersuchungen sind nicht veröffentlicht.

### 2.5 Inzidenz von proximalen Humerusfrakturen

Ein weltweiter Vergleich der Inzidenz von proximalen Humerusfrakturen steht aus den oben erwähnten Gründen ebenfalls nicht zur Verfügung. Einige wenige Länder, wie etwa Finnland oder Australien, haben mehr oder weniger belastbare Daten hierzu veröffentlicht, aber selbst hier sind Ländervergleiche wenig zielführend, da zumeist keine flächendeckenden nationalen Daten, sondern nur Kohortendaten veröffentlicht sind. Österreichische Daten berücksichtigen wiederum sowohl ambulante als auch stationäre Daten, weswegen eine gute Annäherung an die tatsächliche Inzidenz angenommen werden kann [16]. Die durchschnittliche altersstandardisierte Inzidenz im Zeitraum über 20 Jahre (1989–2008) betrug für Frauen 302,5/100.000 und für Männer 126,5/100.000, mit steigender Tendenz in beiden Geschlechtern bis zum Ende der Beobachtungsperiode. Daten aus jüngeren Untersuchungen sind nicht veröffentlicht.

### 2.6 Inzidenz von vertebralen Frakturen

Die Inzidenz vertebraler Frakturen ist jene, welche am schwierigsten von allen MOF zu erfassen ist. Eine der maßgeblichen Ursachen ist, dass neben den unterschiedlichen Definitionen einer vertebralen Fraktur ein Großteil davon (rund zwei Drittel) nicht durch ein erinnerliches Trauma entsteht, sondern durch eine fortschreitende Höhenabnahme der betreffenden Wirbelkörper [17]. Diese langsame Höhenabnahme verursacht zumeist keine akuten Schmerzen, wodurch eine unmittelbare weiterführende radiologische Abklärung ausbleibt. Vielmehr ist es der sich im Verlauf entwickelnde Weichteilschmerz entlang der Wirbelsäule, welcher durch die Verkürzung der paravertebralen Muskulatur bedingt ist. Daten zur echten Inzidenz vertebraler Frakturen stehen daher zumeist nur für die sog. klinisch-vertebralen Frakturen zur Verfügung. Ergebnisse der Global Burden of Disease Study sind daher mit großer Vorsicht zu interpretieren [18]. Eine einigermaßen zuverlässige, aber knapp 2 Dekaden zurückliegende prospektive Studie, an welcher auch Österreich teilnahm, war die European Prospective Osteoporosis Study (EPOS) [17]. Die altersstandardisierte Inzidenz von radiologisch erfassten vertebralen Frakturen betrug in dieser Studie für Frauen 1070/100.000 und für Männer 570/100.000. Daten für das einzige österreichische Zentrum sind aufgrund der zu geringen Fallzahl nicht gesondert veröffentlicht.

### 2.7 Inzidenz von Beckenfrakturen

Beckenfrakturen rücken zunehmend in den Fokus epidemiologischer Untersuchungen, da sie als starker Risikofaktor für Folgefrakturen identifiziert werden konnten und mit einer erhöhten Mortalität assoziiert sind [19, 20]. Inzidenzraten innerhalb der österreichischen Population wurden für einen Zeitraum von 2010 bis 2018 untersucht. Dabei zeigte sich eine steigende altersstandardisierte Inzidenz für Frauen von 219/100.000 auf 225/100.000 und für Männer von 125/100.000 auf 138/100.000. Es ist bemerkenswert, dass im vergleichbaren Zeitraum die altersstandardisierte Inzidenz von hüftnahen Frakturen der österreichischen Population eine sinkende Tendenz aufwies [14].

### 2.8 Epidemiologie „echter“ Fragilitätsfrakturen in Österreich

Eine der wichtigsten Fragestellungen im Zusammenhang mit der epidemiologischen Erfassung von typischen osteoporotischen Frakturen ist jene zur Schwere des jeweiligen der Fraktur zugrunde liegenden Traumas. Insbesondere nationale, auf ICD-Codierungen basierende Daten berücksichtigen die Schwere des Traumas nur unzureichend oder gar nicht. Um diese Unschärfe von Inzidenzzahlen weitgehend zu korrigieren, wurden kürzlich für Österreich unter der Schirmherrschaft der Gesundheit Österreich GmbH (GÖG) die entsprechenden epidemiologischen Daten mittels validierter Methode aufgearbeitet. Für das Jahr 2018 wurde solcherart eine Anzahl von knapp 93.000 „echten“ osteoporotischen Frakturen (einschließlich Rippen‑, Becken- und Tibiafrakturen) ermittelt [21]. Dies entspricht einer Prävalenz von 2600/100.000 in der Altersgruppe ≥ 50 Jahre, wobei rund 72 % der Frakturen auf Frauen entfielen. Die höchste Prävalenz entfiel hierbei auf die distale Unterarmfraktur, gefolgt von Rippenfraktur und hüftnaher Fraktur. Zwischen den 9 Bundesländern gab es kaum Unterschiede in der Frakturinzidenz.

## 3 Risikofaktoren für Osteoporose

### 3.1 Klinische Risikofaktoren

Empfehlungen:

- Klinische Risikofaktoren beeinflussen das individuelle Bruchrisiko unabhängig vom Alter und von der KMD; *starke Empfehlung*.
- Arithmetische Anpassungen der FRAX®-Wahrscheinlichkeit für eine MOF (hüftnahe Fraktur, klinisch vertebrale Fraktur, Unterarmfraktur, Humerusfraktur) oder hüftnahe Fraktur (s. Tab. 5) können in der klinischen Praxis berücksichtigt werden und werden vom FRAX®-Algorithmus automatisch kalkuliert. Ebenfalls gilt dies für zusätzliche klinische Risikofaktoren, wie z. B. eine Glukokortikoidtherapie, eine diskordant niedrige KMD an der Lendenwirbelsäule, einen Diabetes mellitus Typ 2 oder Stürze in der Anamnese; *bedingte Empfehlung*.

Die Vorhersagekraft der KMD-Messung durch DXA kann durch die gleichzeitige Berücksichtigung klinischer Risikofaktoren, die unabhängig von der KMD wirken, verbessert werden. Von besonderer Bedeutung ist das Alter, welches unabhängig von der KMD zum Risiko beiträgt [22, 23]; *Evidenzgrad Ia*.

Einige klinische Risikofaktoren liefern Informationen über das Frakturrisiko unabhängig von Alter und KMD. So gibt es Hinweise auf *geschlechtsspezifische Unterschiede*. Bei Männern > 60 Jahre ist das Frakturrisiko halb so hoch wie bei Frauen gleichen Alters [24]; *Evidenzgrad IIa*.

Ein *niedriger Body Mass Index *(BMI) ist ein signifikanter Risikofaktor für hüftnahe Frakturen. Der Wert des BMI bei der Vorhersage anderer Frakturen ist jedoch sehr viel geringer, wenn dieser mit der KMD adjustiert wird [25]; *Evidenzgrad Ia*.

Eine *vorangegangene Fraktur*, insbesondere wenn sie durch ein geringes Trauma und an einer für Osteoporose charakteristischen anatomischen Lokalisation erlitten wurde, ist ein wichtiger Risikofaktor für weitere Frakturen [5]. Diese Risiken sind zum Teil unabhängig von der KMD [26]. Das Frakturrisiko ist ungefähr doppelt so hoch, wenn bereits eine prävalente nicht-vertebrale Fraktur vorliegt. Eine prävalente vertebrale Fraktur erhöht das Risiko für weitere vertebrale Frakturen um nahezu das 5fache [27]. Das inkludiert asymptomatische moderate oder schwere (Genant Grad 2 oder 3; s. Abschn. 5.2. „Bildgebende Verfahren“) morphometrische Wirbelkörperfrakturen. Eine Wirbelkörperfraktur in der Anamnese ist eine spezielle Situation. Eine rein radiographisch entdeckte Fraktur (also eine morphometrische Wirbelkörperfraktur) zählt als frühere Fraktur. Eine frühere klinische Wirbelkörperfraktur oder eine hüftnahe Fraktur sind besonders gewichtige Risikofaktoren. Das gerechnete Frakturrisiko kann in diesen Fällen unterschätzt sein. Dieses wird auch bei multiplen Frakturen unterschätzt [26, 28]; *Evidenzgrad Ia*. Der Anstieg des Risikos ist bei mehr als einer Wirbelkörperfraktur noch deutlicher. Nach einer Fraktur ist das Risiko einer weiteren Fraktur im unmittelbaren Post-Fraktur-Intervall am höchsten (sog. imminentes Frakturrisiko), wobei mehr als ein Drittel der Folgefrakturen über einen Zeitraum von 10 Jahren innerhalb des ersten Jahres auftreten [29]; *Evidenzgrad Ic*.

Eine hüftnahe Fraktur in der *Anamnese der Eltern* ist ein signifikanter Risikofaktor, der weitgehend unabhängig von der KMD ist [30]; *Evidenzgrad Ia*.

*Aktuelles Rauchen* ist ein Risikofaktor, der auch zum Teil von der KMD abhängig ist [31]; *Evidenzgrad Ia*.

Eine orale *Glukokortikoidtherapie* erhöht das Frakturrisiko in Abhängigkeit von der Dosis (s. Abschn. 3.2. „Sekundäre Osteoporosen“, Tab. 3). Das Frakturrisiko durch Glukokortikoide ist jedoch nicht allein vom Knochenverlust abhängig, es wurden auch KMD-unabhängige Risiken festgestellt [32, 33]; *Evidenzgrad Ia*.

Es gibt viele *sekundäre Ursachen für Osteoporose *(z. B. entzündliche Darmerkrankungen, endokrine Erkrankungen etc.). In den meisten Fällen ist unklar, inwieweit ein erhöhtes Frakturrisiko von einer niedrigen KMD oder anderen Faktoren wie der Einnahme von Glukokortikoiden abhängt (s. Abschn. 3.2. „Sekundäre Osteoporosen“, Tab. 2). Im Gegensatz dazu erhöht die rheumatoide Arthritis das Frakturrisiko unabhängig von der KMD und der Einnahme von Glukokortikoiden [33]; *Evidenzgrad Ia*.

*Diabetes mellitus* (sowohl Typ 1 als auch Typ 2) ist mit einem erhöhten Risiko für hüftnahe und nicht-vertebrale Frakturen verbunden. Bei Diabetes mellitus Typ 2 sind eine längere Krankheitsdauer und Insulingabe mit einem erhöhten Risiko verbunden, das teilweise unabhängig von der KMD ist [34–37]; *Evidenzgrad Ia*.

Der individuelle *Alkoholkonsum* steht in einem dosisabhängigen Verhältnis zum Frakturrisiko. Liegt ein Alkoholkonsum von durchschnittlich 2 Einheiten oder weniger pro Tag vor, wurde keine Erhöhung des Risikos festgestellt. Ein Konsum von 3 oder mehr Einheiten täglich ist mit einem dosisabhängigen Anstieg des Frakturrisikos verbunden. Eine Einheit entspricht 10 ml oder 8 g reinem Alkohol [38]; *Evidenzgrad Ia*.

Die alleinige Verwendung kombinierter klinischer Risikofaktoren zur Vorhersage des Frakturrisikos ist ähnlich gut geeignet wie die alleinige Verwendung der KMD [39]. Die Verwendung von klinischen Risikofaktoren unter Hinzunahme der KMD ist optimal. Die KMD-Messung kann auf Personen angewendet werden, deren individuelles Risiko nahe an der Schwelle zwischen niedrigem/hohem Risiko oder nahe an der Schwelle zu hohem/sehr hohem Risiko liegt (s. Abschn. 6.2. „Bestimmung des Frakturrisikos“).

Es existieren viele zusätzliche klinische Risikofaktoren für Frakturen, die nicht im FRAX® enthalten sind, einschließlich Risiken, die nur durch eine Verringerung der KMD wirken, die weniger gut validiert sind oder die ein Risiko identifizieren, das möglicherweise auf spezifische Behandlungen nicht anspricht [22, 40]. Die Sturzgefahr ist ein Beispiel für Letzteres, bei dem das Frakturrisiko hoch ist. Dieses Risiko wird bei einer Behandlung mit Medikamenten, die den Knochenstoffwechsel beeinflussen, möglicherweise nicht vollständig abgebildet [41].

Im Folgenden (Tab. 1) werden klinische Risikofaktoren vorgestellt und definiert, für welche das relative Frakturrisiko für mindestens eine der MOF (hüftnahe Fraktur, klinisch vertebrale Fraktur, Unterarmfraktur, Humerusfraktur) um mindestens das 1,5fache erhöht ist [42–44].

**Tab. 1:** Klinische Frakturrisikofaktoren

Risikofaktor	Frakturrisiko	Evidenz
*Niedrige KMD* Referenz: DXA Schenkelhals	Eine KMD-Abnahme um eine Standardabweichung steigert das Frakturrisiko um das ca. 2fache [2, 45]	*Evidenzgrad Ia*
	Bei gleicher KMD im Schenkelhals haben Frauen und Männer dasselbe Risiko [46, 47]	*Evidenzgrad IIa*
*Alter*	Frakturrisiko steigt unabhängig von der KMD [22, 23]	*Evidenzgrad Ia*
*Geschlecht*	Lebenszeitrisiko für osteoporotische Fraktur bei Frauen 40–50 %, bei Männern 13–22 % [24]	*Evidenzgrad IIa*
*Niederer BMI*	Vor allem hüftnahe Fraktur [25] Verglichen mit einem BMI von 25 kg/m^2^ ist ein BMI von 20 kg/m^2^ mit einem ca. 2fachen Anstieg des Risikos für eine hüftnahe Fraktur assoziiert [25]	*Evidenzgrad Ia*
*Vorbestehende Fraktur*	Risiko verdoppelt bis nahezu verfünffacht für Folgefraktur insbesondere nach niedrig traumatischer Fraktur in für Osteoporose typischer Lokalisation (auch bei asymptomatischer moderater oder schwerer morphometrischer Wirbelkörperfraktur) [26–28]	*Evidenzgrad Ia*
	*Imminentes Risiko*: Risiko einer Folgefraktur am höchsten unmittelbar nach einer Fraktur, mehr als ein Drittel der Folgefrakturen (über 10 Jahre) im ersten Jahr [29, 48]	*Evidenzgrad Ic*
*Hüftnahe Fraktur bei Eltern*	RR 1,54 für osteoporotische Fraktur, RR 2,27 für hüftnahe Fraktur, Anstieg weitgehend unabhängig von KMD [30]	*Evidenzgrad Ia*
*Rauchen*	Gegenwärtiges Rauchen: RR für hüftnahe Fraktur 1,84. Bei Raucheranamnese Frakturrisiko höher als durch KMD zu erwarten [31]	*Evidenzgrad Ia*
*Alkoholkonsum*	Frakturrisiko steigt dosisabhängig ab ≥ 3 Einheiten Alkohol/Tag (1 Einheit: 8–10 g Alkohol) Abnahme der KMD und vermehrt Stürze RR 1,38 für osteoporotische Fraktur, RR 1,68 für hüftnahe Fraktur [38]	*Evidenzgrad Ia*
*Orale Glukokortikoidtherapie*	Frakturrisiko steigt dosisabhängig [32, 33] Siehe auch GIOP (Abschn. 3.2. „Sekundäre Osteoporosen“)	*Evidenzgrad Ia*
*Sturzanamnese*	Sturz innerhalb von 4 Monaten bei älteren Frauen: im Folgejahr 2,5fach höheres nicht-vertebrales Frakturrisiko und 3,1fach höheres Risiko einer hüftnahen Fraktur im Vergleich zu Frauen ohne Stürze (s. Abb. 1). Ähnliches Risikoprofil bei Männern [49] Frakturrisikosteigerung für MOF und Hüfte um 30 % bei wiederholten Stürzen (≥ 2 Stürze im letzten Jahr) [44, 50]	*Evidenzgrad Ib*
*Immobilität*	Erhöhtes Frakturrisiko bei Personen, die in ihrer Mobilität stark eingeschränkt sind Erhöhtes Risiko für MOF und hüftnahe Fraktur bei Frauen (75 bis 80 Jahre), wenn Timed-up-and-Go-Test > 12 s [51]	*Evidenzgrad IIb*
*Apoplektischer Insult*	2fach erhöhtes Risiko für hüftnahe Fraktur [52]	*Evidenzgrad Ia*
*Morbus Alzheimer/Demenz*	2fach erhöhtes Frakturrisiko [53]	*Evidenzgrad Ia*
*Chronische Hyponatriämie ≤* *135* *mmol/l*	Sturzrisiko erhöht 2fach erhöhtes Frakturrisiko, v. a. hüftnahe Fraktur [54]	*Evidenzgrad Ia*
*Thoraxkyphose, Höhenverlust >* *4* *cm*	Erhöhtes Frakturrisiko [44, 55]	*Evidenzgrad IIa*
*Sekundäre Osteoporosen*	Siehe Abschn. 3.2. „Sekundäre Osteoporosen“	*Evidenzgrad Ia*
*Arzneimittelinduzierte Osteoporose*	Siehe Abschn. 3.2. „Sekundäre Osteoporosen“	*Evidenzgrad Ia*

*BMI* Body Mass Index, *DXA* 2-Spektren-Röntgenabsorptiometrie, *GIOP* glukokortikoidinduzierte Osteoporose, *KMD* Knochenmineraldichte, *MOF* „major osteoporotic fracture“ (hüftnahe Fraktur, klinisch vertebrale Fraktur, Unterarmfraktur, Humerusfraktur), *RR* relatives Risiko

**Abb. 1:**
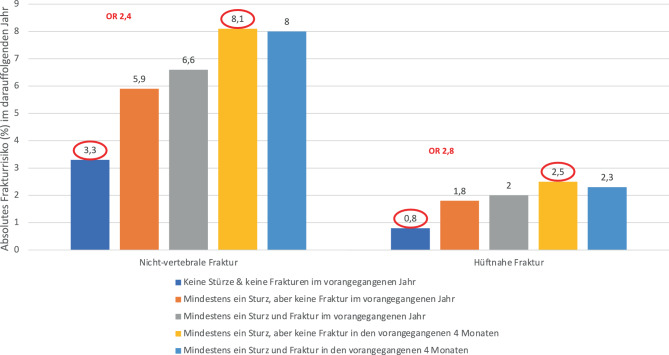
*Absolutes Risiko* *(%) von nicht-vertebralen Frakturen und hüftnahen Frakturen bei älteren Frauen und Männern in Abhängigkeit von Stürzen in den vorangegangenen 4* *Monaten oder im vorangegangenen Jahr. OR* Odds Ratio. (Reproduziert und übersetzt mit entsprechender Lizenz von [49])

Neben allgemeinen Risikofaktoren für klinische Frakturen stellen auch Erkrankungen, die zu einer sekundären Osteoporose führen können, spezifische Risikofaktoren dar. Längerfristig verabreichte Medikamente können ebenfalls ein erhöhtes Frakturrisiko bewirken (arzneimittelinduzierte Osteoporose). Diese werden detailliert im folgenden Abschn. 3.2. „Sekundäre Osteoporosen“ dargestellt.

### 3.2 Sekundäre Osteoporosen

Die sekundäre Osteoporose ist definiert als Knochenmineralverlust, verursacht durch spezifische, klar definierte Erkrankungen [56]. Im weiteren Sinne zählt dazu auch die arzneimittelinduzierte Osteoporose. Eine sekundäre Ursache für die Osteoporose liegt bei 50 % der prämenopausalen Frauen, 30 % der postmenopausalen Frauen und 66 % der Männer mit Osteoporose vor [57]. Der Nachweis einer sekundären Ursache ist von großer Bedeutung, da die Behandlung der zugrunde liegenden Erkrankung ein wesentlicher Teil der Osteoporosetherapie ist.

Einige sekundäre Osteoporosen werden bei der Frakturrisikobestimmung mittels FRAX® berücksichtigt (für eine detaillierte Auflistung s. Tab. 2, 3 und 4). Alle anderen sekundären Osteoporoseformen sind als Risikofaktoren zu berücksichtigen, und eine Frakturrisikoeinschätzung wird empfohlen. Pharmakologische Interventionsschwellen können bei sekundärer Osteoporose von denen bei primärer Osteoporose differieren. Auch eine Kombination von primärer und sekundärer Osteoporose ist im Einzelfall in Betracht zu ziehen.

**Tab. 2:** Erkrankungen, die eine sekundäre Osteoporose verursachen können

Erkrankung	Ergänzung	Evidenz
***Endokrine Erkrankungen***		
**Cushing-Syndrom, subklinischer Hyperkortisolismus**	KMD und TBS reduziert bei aktiver Erkrankung Frakturrisiko bei Frauen und Männern erhöht, teilweise unabhängig von KMD, teilweise reversibel nach Behandlung der Grunderkrankung Keine Validierung für FRAX® für endogenen Hyperkortisolismus [58–61]	*Evidenzgrad IIIb*
**Hyperthyreose**, manifest und subklinisch	FRAX®-Score: „sekundäre Osteoporose“ [62]	*Evidenzgrad Ia*
**Diabetes mellitus Typ 1**	FRAX®-Score: „sekundäre Osteoporose“ Erhöhtes Risiko für hüftnahe Frakturen und periphere Frakturen [34]	*Evidenzgrad Ia*
**Diabetes mellitus Typ 2**	FRAX®-Score: Adjustierung „Rheumatoide Arthritis“ Erhöhtes Risiko für hüftnahe Frakturen und periphere Frakturen. Steigendes Frakturrisiko durch Erkrankungsdauer und Insulintherapie, teilweise unabhängig von KMD und bei HbA_1c_ > 7 [34, 63, 64]	*Evidenzgrad Ia*
**Hypogonadismus beim Mann**	FRAX®-Score: „sekundäre Osteoporose“ Erniedrigtes Serumtestosteron assoziiert mit erhöhtem Frakturrisiko [65]	*Evidenzgrad IIa*
**Hypogonadismus bei Frauen (Östrogenmangel)**	FRAX®-Score: „sekundäre Osteoporose“ [66] Primäre Amenorrhö, späte Menarche, frühe Menopause (< 45 Jahre) [42]	*Evidenzgrad IIb*
**Primärer Hyperparathyreoidismus**	Frakturrisiko erhöht, OR 2,01 [67]	*Evidenzgrad Ia*
**Wachstumshormonmangel bei Hypophyseninsuffizienz**	Bei Wachstumshormonmangel 2,66fach höhere Frakturrate als bei normalem Wachstumshormonspiegel [68]	*Evidenzgrad IIIb*
***Chronisch entzündliche Erkrankungen***		
**Rheumatoide Arthritis**	FRAX®-Score: „Rheumatoide Arthritis“ Bis zu ein Drittel der Patienten haben Osteoporose, Hüft- und Wirbelkörperfrakturen [69, 70]	*Evidenzgrad Ia*
	Niedrig dosierte Glukokortikoidtherapie bei rheumatoider Arthritis scheint das Frakturrisiko nicht zu erhöhen, der Risikofaktor „rheumatoide Arthritis“ bleibt jedoch erhalten [71]	*Evidenzgrad IIIb*
**Spondylitis ankylosans**	Erhöhtes Risiko für Wirbelkörperfrakturen [72]	*Evidenzgrad Ia*
**Chronische Atemwegserkrankungen** (COPD, Asthma, zystische Fibrose, interstitielle Lungenerkrankungen/Sarkoidose)	Osteoporoseprävalenz bei COPD 38 % [73–75]	*Evidenzgrad Ia*
**Tuberkulose**	Risiko für hüftnahe Frakturen am höchsten [76]	*Evidenzgrad Ib*
***Nierenerkrankung***		
**CKD**	CKD assoziiert mit erhöhtem Risiko für hüftnahe Frakturen und nicht-vertebrale Frakturen. Frakturrisiko steigt mit Abnahme der Nierenfunktion [77–79]	*Evidenzgrad Ia*
***Neuromuskuläre Erkrankungen***		
**Epilepsie bzw. Antiepileptika**	Frakturrisiko erhöht, häufig im Rahmen von Anfällen [80]	*Evidenzgrad Ia*
	Leberenzym-induzierende Antiepileptika assoziiert mit höherem Frakturrisiko vs. nicht-Leberenzym-induzierende Antiepileptika [81]	*Evidenzgrad IIb*
**Morbus Parkinson**	Risiko für hüftnahe Frakturen und nicht-vertebrale Frakturen erhöht [82], Stürze um das 3fache vermehrt [69]	*Evidenzgrad Ia*
**Multiple Sklerose**	Erhöhtes Frakturrisiko bei Frauen unabhängig von Medikation und Sturzrisiko [83]	*Evidenzgrad Ia*
***Gastrointestinale Erkrankungen***		
**Chronisch entzündliche Darmerkrankungen**	FRAX®-Score: „sekundäre Osteoporose“ Morbus Crohn, Colitis ulcerosa Erhöhtes Frakturrisiko, signifikant für Wirbelkörperfrakturen [84]	*Evidenzgrad Ia*
**Laktoseintoleranz**	FRAX®-Score: „sekundäre Osteoporose“ Niedrigere KMD bei laktoseintoleranten postmenopausalen Frauen vs. gesunde Kontrollen [85]	*Evidenzgrad Ia*
**Chronische Lebererkrankungen**	FRAX®-Score: „sekundäre Osteoporose“ Erhöhtes Frakturrisiko bei Leberzirrhose: Alkohol-induziert, primär biliäre Zirrhose, Hepatitis C [86–88]	*Evidenzgrad Ia*
**Zöliakie**	FRAX®-Score: „sekundäre Osteoporose“ – Malabsorption [89]	*Evidenzgrad Ia*
**Bariatrische Chirurgie**	FRAX®-Score: „sekundäre Osteoporose“ – Malabsorption [90]	*Evidenzgrad Ia*
***Ernährungsstörung***		
**Anorexia nervosa**	FRAX®-Score: „sekundäre Osteoporose“ – vermehrtes Auftreten von Osteoporose (OR 12,59) und Frakturen (OR 1,84) [91]	*Evidenzgrad Ia*
***Herzinsuffizienz***	HR für alle Frakturen 1,67, für hüftnahe Frakturen 2,2 [92]	*Evidenzgrad Ia*
***Osteoporose nach Transplantation***	Im FRAX®-Score anklicken: „*Glukokortikoide“: bei Glukokortikoidtherapie* *„Sekundäre Osteoporose“: bei postoperativer immunsuppressiver Therapie nach Stammzelltransplantation, Knochenmarkstransplantation, Transplantation von Herz, Lunge, Leber, Niere [*93*]*	*Evidenzgrad IIa*
***HIV, AIDS***	AIDS per se und antiretrovirale Therapie. OR 2,3 für Wirbelkörperfrakturen bei HIV-infizierten Personen [94]	*Evidenzgrad Ia*
***Hämatoonkologische Erkrankungen***		
**Malignome**	Maligne Erkrankungen per se und Chemotherapie/endokrine Therapie/Strahlentherapie [95]	*Evidenzgrad IIa*
**Multiples Myelom**	Bei Diagnosestellung bei 20 % der Patienten pathologische Fraktur oder Osteoporose, bei 20–50 % vertebrale Frakturen [69, 96]	*Evidenzgrad IIa*
**MGUS**	Frakturrisiko erhöht (RR 1,36), v. a. für vertebrale Frakturen (RR 2,5) [97]	*Evidenzgrad Ia*
**Systemische Mastozytose**	10-Jahres-Frakturrisiko 31 % [98]	*Evidenzgrad IIa*

*AIDS* akquiriertes Immundefizienzsyndrom, *CKD* chronische Nierenerkrankung, *COPD* chronisch obstruktive Atemwegserkrankung, *FRAX* Fracture Risk Assessment Tool, *HIV* humanes Immundefizienzvirus, *HR* Hazard Ratio, *KMD* Knochenmineraldichte, *MGUS* monoklonale Gammopathie unklarer Signifikanz, *OR* Odds Ratio, *RR* relatives Risiko, *TBS* Trabecular Bone Score

**Tab. 3:** Arzneimittelinduzierte Osteoporose; Medikamente, die mit einem erhöhten Frakturrisiko assoziiert sind

Medikament	Ergänzung	Evidenz
**Systemische Glukokortikoide (GIOP)**	FRAX®-Score: „Glukokortikoide“, wenn aktuell oder über 3 Monate tägliche Dosis ≥ 5 mg Prednisolonäquivalent Rascher Anstieg des Frakturrisikos (v. a. vertebrale Frakturen) Zusammenhang KMD und kumulative Glukokortikoiddosis [99]	*Evidenzgrad Ia*
	Hohe kumulative Glukokortikoiddosen (≥ 1 g) stellen einen Risikofaktor für hüftnahe Frakturen und vertebrale Frakturen dar [100] Initial vermehrte Osteoklastenaktivität, langfristig Suppression der Osteoblastenfunktion [33]	*Evidenzgrad IIIb*
	Das Frakturrisiko unter inhalativen Glukokortikoiden ist zum jetzigen Zeitpunkt nicht vollständig geklärt. Inhalative Glukokortikoide stellen einen maximal schwachen Risikofaktor für osteoporotische Frakturen dar [101] Frakturrisiko (v. a. vertebral) dosisabhängig; bereits ab Glukokortikoidtherapiebeginn knochenprotektive Behandlung bei – *vorbestehender Fragilitätsfraktur* – *Frauen ab 70 Jahren* – *bei postmenopausalen Frauen und Männern ab 50 Jahren, bei ≥* *7,5* *mg Prednisolon oder Äquivalent/Tag über 3 Monate* – *bei Überschreiten der FRAX®-Therapieschwelle [*44*]* Anmerkung: Korrektur des Frakturrisikos nach Glukokortikoiddosis (s. Tab. 4) Empfehlungen für GIOP bei prämenopausalen Frauen und jungen Männern s. ACR-Leitlinien [102]	*Evidenzgrad Ia*
**Aromatasehemmer bei Mammakarzinom**	Hohes Frakturrisiko bei Patientinnen und Patienten für alle Frakturen unabhängig von der initialen KMD [103]	*Evidenzgrad Ib*
**Hormonablative Therapie Frauen (GnRH-Analoga)**	Geringes Frakturrisiko [104]	*Evidenzgrad IIa*
**Hormonablative Therapie bei Prostatakarzinom (Androgen-Rezeptor-Inhibitoren)**	GnRH-Agonisten: Bei Männern steigt das Frakturrisiko mit der Behandlungsdauer [104, 105]	*Evidenzgrad Ia*
**Depot-Medroxyprogesteron-Acetat**	Erhöhtes Frakturrisiko unter DMPA-Therapie – Östrogendefizit und Glukokortikoideffekt [69, 106]	*Evidenzgrad IIIb*
**Antidepressiva – selektive Serotonin-Reuptake-Inhibitoren**	Erhöhtes Frakturrisiko unter SSRI-Therapie [107]	*Evidenzgrad Ia*
**Neuroleptika**	Erhöhtes Frakturrisiko – hüftnahe Fraktur OR 1,46 [108]	*Evidenzgrad Ia*
**Protonenpumpeninhibitoren**	Hüftfrakturrisiko erhöht, steigend, je höher die PPI-Dosis. Alternative: H_2_-Blocker [109]. Langfristige gleichzeitige Einnahme von PPI und oralen Bisphosphonaten sollte vermieden werden [110]	*Evidenzgrad Ia*
**Glitazone**	Erhöhtes Risiko für periphere Frakturen, kein primärer Einsatz bei hohem Frakturrisiko, v. a. bei postmenopausalen Frauen [64, 111]	*Evidenzgrad Ia*
**Antikoagulanzien**	Gering erhöhtes Frakturrisiko bei VKA-Langzeittherapie bei Frauen und bei älteren Personen ab 65 Jahren [112]	*Evidenzgrad Ia*

*ACR* American College of Rheumatology, *DMPA* Depot-Medroxyprogesteron-Acetat, *FRAX* Fracture Risk Assessment Tool, *GIOP* glukokortikoidinduzierte Osteoporose, *GnRH* Gonadotropin-Releasing-Hormon, *KMD* Knochenmineraldichte, *PPI* Protonenpumpeninhibitoren, *SSRI* selektive Serotonin-Reuptake-Inhibitoren, *VKA* Vitamin-K-Antagonisten

**Tab. 4:** Korrektur des Frakturrisikos nach FRAX® abhängig von der täglichen Glukokortikoiddosis

Dosis	Korrektur für Wahrscheinlichkeit einer hüftnahen Fraktur (%)	Korrektur für MOF-Wahrscheinlichkeit (%)
< 2,5 mg/Tag	−35	−20
≥ 7,5 mg/Tag	+20	+15

*FRAX* Fracture Risk Assessment Tool, *MOF* „major osteoporotic fracture“ (hüftnahe Fraktur, klinisch vertebrale Fraktur, Unterarmfraktur, Humerusfraktur)

Die Dosis entspricht Prednisolon-Äquivalenten. Für die Dosierung 2,5–7,5 mg ist keine Korrektur vorzunehmen (adaptiert nach [113]). Im FRAX® ist vorgesehen, orale Glukokortikoide als Risikofaktor anzuklicken, wenn ein Patient aktuell oder jemals einen oralen Glukokortikoidschwellenwert von zumindest 5 mg über 3 Monate erhält oder erhalten hat. Eine individuell höhere oder niedrigere Dosis kann mit den Korrekturfaktoren adaptiert werden

Bei weiteren seltenen Formen von Erkrankungen (auch genetischen Erkrankungen) und/oder Konditionen mit erhöhtem Frakturrisiko wird auf die Empfehlungen der jeweiligen Fachgesellschaften verwiesen.

## 4 Prävention und Basismaßnahmen

Die KMD sowie die Mikroarchitektur des trabekulären Knochens wie auch die Dicke des spongiösen Knochens reagieren sensibel auf Lebensstilfaktoren. Auf der einen Seite spielt die Ernährung eine große Rolle für die Versorgung mit Kalzium und Vitamin D und hat damit die zentrale Bedeutung für die ausreichende Versorgung mit Knochenbausteinen inne, und auf der anderen Seite stehen Bewegung und Sport, die durch ihre mechanischen Stimuli Knochenaufbauprozesse ortsspezifisch und funktionsgebunden anregen. Im folgenden Abschn. 4.1. „Prävention durch Ernährung und Lebensstilveränderungen“ werden diese Faktoren in einem präventiven Kontext aufgearbeitet.

### 4.1 Prävention durch Ernährung und Lebensstilveränderungen

Empfehlungen:

- Eine tägliche Zufuhr von zumindest 1000 mg Kalzium, 800 IE natives Vitamin D und 0,8 g/kgKG (Sollgewicht) an Protein wird empfohlen, bei > 65-Jährigen wird mindestens 1 g/kgKG Protein empfohlen; *starke Empfehlung*.
- Besondere Ernährungsformen oder Unverträglichkeiten wie vegane Ernährung oder Laktoseintoleranz können problematisch sein und sollten im Rahmen einer individuellen Ernährungsberatung thematisiert werden; *bedingte Empfehlung*.

Eine adäquate Ernährung für die Prävention einer Osteoporose ist eine fundamentale Basismaßnahme, insbesondere für das Erreichen der bestmöglichen individuellen Peak Bone Mass, der maximal erreichbaren Knochenmasse [114]. Ein niedriger BMI ist ein bedeutender Risikofaktor für hüftnahe Frakturen, wobei die Vorhersagekraft geringer wird, wenn um die KMD bereinigt wird; *Evidenzgrad Ia*.

Besondere Ernährungsformen wie vegane Ernährung oder Unverträglichkeiten wie Laktoseintoleranz sollten im Rahmen einer individuellen Ernährungsberatung thematisiert werden. Eine vegane Ernährung wurde mit einem erhöhten Frakturrisiko assoziiert; *Evidenzgrad IIa*. Eine prospektive Kohortenstudie mit 65.000 Personen zeigte eine niedrigere KMD an der Wirbelsäule und Hüfte bei Veganern und Vegetariern sowie ein höheres Risiko für hüftnahe Frakturen bei Veganern; dies wurde durch Anpassung der Kalzium- bzw. Proteinzufuhr teilweise abgeschwächt; *Evidenzgrad IIb*.

Die Knochengesundheit kann durch allgemeine und spezifische Maßnahmen unterstützt werden. Eine *ausgewogene Ernährung* mit ausreichend Obst und Gemüse, reich an antioxidativen Vitaminen und Mineralstoffen, kann die Knochengesundheit unterstützen; *Evidenzgrad IIa***.** Eine randomisiert kontrollierte Studie über eine „gesunde Ernährung“ über 30 Tage mit Schwerpunkt auf eine kalziumreiche Ernährung mit Obst, Gemüse und fettarmen Milchprodukten (Dietary Approaches to Stop Hypertension, DASH), führte zu einem Rückgang der Knochenumbaumarker [115]; *Evidenzgrad Ib*.

#### *Optimierung der Kalziumzufuhr:*

Der Referenzwert der Deutschen und der Österreichischen Gesellschaften für Ernährung (DGE/ÖGE) für die tägliche Kalziumzufuhr ist 1000 mg für Erwachsene [116]; *Evidenzgrad IV*. Diese kann durch den Verzehr von Milchprodukten (laktosefreie bei Laktoseintoleranz), kalziumreichem Gemüse sowie anderen Lebensmitteln wie Sesam oder Mohn erreicht werden. Vegane kalziumsupplementierte Milchalternativen oder Mineralwässer können ebenfalls dazu verwendet werden. Sollten die 1000 mg nicht erreichbar sein, wird ein ergänzendes orales Kalziumsupplement empfohlen.

#### *Vitamin D:*

Eine tägliche Vitamin-D-Zufuhr von 20 µg (800 IE) für Erwachsene wird empfohlen [116]. Ein adäquater Vitamin-D-Spiegel von zumindest ≥ 20 ng/ml bzw. ≥ 50 nmol/l wird empfohlen, ein Vitamin-D-Spiegel von ≥ 50 ng/ml bzw. ≥ 125 nmol/l ist aus osteologischer Sicht aber nicht erforderlich. Neben endogener Synthese durch UVB/Sonnenexposition können Vitamin-D-reiche Nahrungsmittel wie fetter Fisch, Eigelb und angereicherte Lebensmittel berücksichtigt werden. Zumeist ist jedoch (zumindest in den Wintermonaten) eine Supplementierung von nativem Vitamin D sinnvoll/erforderlich [116]; *Evidenzgrad IV*.

#### *Protein:*

Eine tägliche Proteinzufuhr von zumindest 0,8 g pro Kilogramm Körpergewicht (kgKG) (Sollgewicht) und bei über 65-Jährigen von 1,0 g/kgKG wird von der DGE/ÖGE empfohlen [116]. Es ist unklar, ob eine höhere Zufuhr über dieser Grenze die Knochengesundheit verbessert, möglicherweise besteht ein niedrigeres Hüftfrakturrisiko bei höherer vs. niedrigerer Zufuhr [117]. Die European Society for Parenteral and Enteral Nutrition (ESPEN) empfiehlt bereits seit Längerem eine höhere Zufuhr von 1,0–1,5 g bei Menschen über 65 Jahren [118].

Diese Zufuhr sollte erhoben werden, da sie von vielen älteren Menschen nicht erreicht wird und der Proteinbedarf im Alter (ab 65 Jahren) eher noch höher wird; *Evidenzgrad IV*. Auch das bei Osteoporose empfohlene Krafttraining kann nur bei ausreichender Proteinzufuhr zum Erhalt bzw. zu einem Zuwachs an Muskelmasse und -kraft führen, um Sarkopenie zu vermeiden. Mageres Fleisch, Geflügel, Fisch, Milchprodukte, Eier, Vollkornprodukte, Nüsse und Samen sowie Hülsenfrüchte sind mögliche empfehlenswerte Proteinquellen. Im Einzelfall kann auch die Zufuhr von Shakes oder Proteinpulver (insbesondere Molkeprotein oder auch vegane Alternativen, wie z. B. Erbsenprotein) sinnvoll sein, um die Zufuhr zu gewährleisten. Dies sollte besonders bei veganer Ernährung berücksichtigt werden.

#### *Beschränkung des Alkoholkonsums:*

Ein maximal moderater Alkoholkonsum wird empfohlen, wobei Frauen auf maximal 10 g und Männer auf höchstens 20 g Alkohol pro Tag kommen sollten [116]; *Evidenzgrad IV*. Alkohol erhöht dosisabhängig das Frakturrisiko. Bei einer Aufnahme von 3 oder mehr Einheiten pro Tag ist ein dosisabhängiger Anstieg des Frakturrisikos zu verzeichnen. Eine Einheit entspricht 10 ml oder 8 g reinem Alkohol [38]; *Evidenzgrad Ia*.

#### *Gewichtsmanagement/metabolisches Syndrom/Diabetes mellitus:*

Ein gesundes Körpergewicht sollte angestrebt werden. Die Entwicklung eines Diabetes mellitus kann mit Reduktion von einfachen Kohlenhydraten sowie ausreichender körperlicher Aktivität verzögert oder verhindert werden; *Evidenzgrad IV*.

Erkrankungen, die zu einer eingeschränkten Knochengesundheit führen können, sind unter anderem Zöliakie, Laktoseintoleranz, Diabetes mellitus Typ 1 und Typ 2 sowie jede Art von Malnutrition inklusive Anorexie; *Evidenzgrad IV*; oder stattgehabtem bariatrischem Eingriff – abhängig von der Art des operativen Vorgehens [90]; *Evidenzgrad Ia.*

### 4.2 Prävention durch Bewegung und Sport

Empfehlungen:

- In der Jugend wie im jungen Erwachsenenalter wird regelmäßige körperliche Aktivität zur Reduktion eines späteren Osteoporoserisikos empfohlen; *starke Empfehlung*.
- Postmenopausalen Frauen und Männern ≥ 50 Jahre wird zur Verbesserung der KMD und Prävention von Fragilitätsfrakturen eine Kombination von abrupt gewichtsbelastendem Training („Impact Training“) und Krafttraining empfohlen; *starke Empfehlung*.
- Die Trainingsfrequenz bei Krafttraining soll mindestens 2‑mal/Woche betragen und, basierend auf der Ausgangssituation, individualisiert geplant werden; *starke Empfehlung*.
- Die Trainingsfrequenz bei Gleichgewichtstraining soll idealerweise bis zu 7‑mal/Woche betragen und, basierend auf der Ausgangssituation, individualisiert und ggf. in den Alltag eingebaut geplant werden; *starke Empfehlung*.
- Das Training soll nach Möglichkeit supervidiert ausgeführt werden; *starke Empfehlung*.
- Eine Sturzrisikoeinschätzung sollte bei allen Patienten > 65 Jahre durchgeführt werden; jenen mit erhöhtem Sturzrisiko sollte ein Übungsprogramm zur Verbesserung von Balance und Kraft empfohlen werden; *starke Empfehlung*.

Regelmäßige körperliche Aktivität und körperliches Training in der Kindheit und Jugend sind wesentlich, um eine optimale Entwicklung der Knochenmasse und -struktur über die Lebenszeit zu erreichen; dies reduziert das Osteoporoserisiko im späteren Lebensalter [114]; *Evidenzgrad Ia*. Im jungen Erwachsenalter [119]; *Evidenzgrad Ia*; und im etwas fortgeschritteneren Lebensalter hat Training ebenfalls positive Effekte auf die KMD [120–124]; *Evidenzgrad Ia*. Das gilt für früh- wie spätpostmenopausale Frauen mit normaler, osteopenischer oder osteoporotischer KMD [123]; *Evidenzgrad IIa* (Subgruppenanalyse); und Männer [122]; *Evidenzgrad Ia*. Positive Effekte von körperlicher Aktivität auf die Knochengesundheit bestehen auch bei Personen, die systemische Glukokortikoide einnehmen müssen [125]; *Evidenzgrad Ia*.

Im Hinblick auf die Kombination einer Osteoporose-spezifischen Therapie und körperlicher Aktivität konnte gezeigt werden, dass die Wirksamkeit der osteoanabolen Therapie durch Ganzkörpervibrationstraining verbessert wird [126]; *Evidenzgrad Ib*. Geeignet zur Verbesserung der KMD ist die Kombination von gewichtsbelastendem Training und Widerstandstraining bei prämenopausalen Frauen [127]; *Evidenzgrad Ia*; postmenopausalen Frauen [120, 121, 123]; *Evidenzgrad Ia*; und Männern [122]; *Evidenzgrad Ia*.

Beispiele für gewichtsbelastendes Training sind Walken, Laufen oder Tanzen. Was die Trainingssteuerung beim Krafttraining betrifft, gibt es für die Sicherheit der Austestung des Einwiederholungsmaximums (1-WH_max_) bei Osteoporosepatienten keinen klaren Beleg. Entsprechend sollte aus Sicherheitsgründen die Trainingssteuerung mittels Mehrfachwiederholungsmaxima (x-WH_max_) empfohlen werden [128]; *Evidenzgrad IV*. Moderate Trainingsintensitäten zeigen regionsübergreifend (Lendenwirbelsäule, Oberschenkelhals, Hüfte) die konsistentesten Effekte auf die KMD, wobei hochintensives Training die größten Verbesserungen der KMD in der Lendenwirbelsäule erzielt [129, 130]; *Evidenzgrad Ia*. Eine Trainingsfrequenz von mindestens 2 Trainingseinheiten pro Woche führte zu größeren Steigerungen der KMD im Lumbalbereich als eine geringere Trainingshäufigkeit [131]; *Evidenzgrad Ia*. Im Hinblick auf die Frakturrisikoreduktion zeigten sich überwiegend supervidierte Trainingseinheiten effektiver als nicht-supervidiertes Training [132]; *Evidenzgrad Ia*.

Da die meisten Frakturen im Zuge eines Sturzes auftreten, ist die Reduktion des Sturzrisikos ein wesentlicher Ansatz in der Frakturprävention (s. auch Abschn. 7.1. „Rehabilitation“). In den Richtlinien der Task Force on Global Guidelines for Falls in Older Adults stellt körperliche Aktivität bzw. das Training einen ganz wesentlichen Bestandteil der Sturzprävention dar [133]; *Evidenzgrad Ia*.

Einer Metaanalyse zufolge [134]; *Evidenzgrad Ia*; reduzieren Ganzkörpervibrationstraining und die Kombination von Balancetraining, funktionellen Übungen und Krafttraining das Sturzrisiko bei selbstständig lebenden älteren Personen [135]; *Evidenzgrad Ia*. In einzelnen Studien zeigen sich positive Effekte von Ganzkörpervibrationstraining auf die lumbale KMD, erfordern aber sehr hohe Trainingsumfänge [136]; *Evidenzgrad Ia*. Zum aktuellen Zeitpunkt können aufgrund der hohen Heterogenität der Interventionen keine methodischen Empfehlungen ausgesprochen werden.

Nach entsprechender Einschulung kann ein Übungsprogramm zur Verbesserung von Gleichgewicht und Kraft auch sehr gut in den Alltag integriert werden und vermindert bei sturzgefährdeten Personen die Sturzrate um über 30 % [137]; *Evidenzgrad Ib*. Eine Metaanalyse zeigte, dass die positiven Effekte von Training auf die KMD und das Sturzrisiko zu einer hochsignifikanten Reduktion osteoporotischer Frakturen führen [138]; *Evidenzgrad Ia*.

Es gibt keine Kontraindikation für körperliche Aktivität/Training! Das Trainingsprogramm muss lediglich an die Limitationen (z. B. kardiovaskuläre Probleme) der Betreffenden adaptiert werden [139]. Dem Algorithmus zum Management postmenopausaler Osteoporose entsprechend, sollen alle Betroffenen angepasst an ihr Frakturrisiko körperlich aktiv sein (risikogerechtes Training, eventuell mit Sturzprävention) [140].

## 5 Diagnose

Das vorliegende Kapitel befasst sich mit der Diagnostik der Osteoporose und den entsprechenden bildgebenden Untersuchungen.

### 5.1 Anamnese und Klinik

Empfehlungen:

- Osteoporose zeigt vor der ersten osteoporotischen Fraktur keine spezifischen klinischen Symptome; *starke Empfehlung*.
- Eine Abnahme der Körpergröße von ≥ 4 cm ist ein möglicher Hinweis auf eine manifeste Osteoporose mit einer Wirbelkörperfraktur; *starke Empfehlung*.
- Bei postmenopausalen Frauen und bei allen Männern ab dem 50. Lebensjahr mit einem klinischen Risikofaktor für eine Fraktur wird jedenfalls eine FRAX®-Analyse ohne KMD-Messung empfohlen; *starke Empfehlung*.

Niedrigtraumatische Frakturen (MOF: hüftnahe Fraktur, klinisch vertebrale Fraktur, Unterarmfraktur, Humerusfraktur) prägen das klinische Bild der Osteoporose und führen zu Schmerzen, Immobilisierung, Funktionseinschränkungen und erhöhter Mortalität. Die Frage nach stattgefundenen niedrigenergetischen Knochenbrüchen als Teil einer routinemäßigen Anamnese mit dem Patienten führt zur Diagnosestellung und zur Therapieindikation, unabhängig von der KMD in der DXA-Messung [8, 42]; *Evidenzgrad Ia und Ib*.

Die klinischen Symptome einer Osteoporose vor dem ersten osteoporotischen Bruch sind unspezifisch und kaum richtungsweisend. Unser Ziel muss die Diagnosestellung vor dem ersten Knochenbruch sein. Bei der Identifizierung von Risikopatienten spielen zahlreiche klinische und andere Risikofaktoren eine Rolle (s. auch Abschn. 3.1. „Klinische Risikofaktoren“ und 3.2. „Sekundäre Osteoporosen“).

Bei Vorliegen von Risikofaktoren für Osteoporose (Alter ≥ 50 Jahre, weibliches Geschlecht, positive Familienanamnese – hüftnahe Fraktur eines Elternteils –, Immobilisierung, niedriger BMI von < 20 kg/m^2^, Rauchen, Alkohol) sollte daher eine weitere klinische Abklärung erfolgen, auch wenn die oder der Betroffene keine klinischen Symptome aufweist [42, 44, 141]; *Evidenzgrad Ia und Ib*.

Im Rahmen einer solchen klinischen Untersuchung ist die Körpergröße zu messen. Eine Abnahme von ≥ 4 cm im Vergleich zur maximalen Körpergröße (z. B. Angabe im Reisepass) ist ein wichtiger Indikator für eine vorliegende Wirbelkörperfraktur. Ein standardisiertes Wirbelsäulenröntgen in 2 Ebenen (anterior-posterior und lateral) sollte veranlasst werden, um eventuelle Wirbelkörpereinbrüche zu detektieren; alternativ kann eine laterale Darstellung der Wirbelsäule mittels DXA (sog. vertebrales Frakturassessment [VFA]) erfolgen (s. auch Abschn. 5.2. „Bildgebende Verfahren“). Auch eine physikalische Untersuchung kann hilfreich sein (Untersuchung des Rückens auf eine Rundrückenbildung oder Bildung von Hautfalten [„Tannenbaumphänomen“]).

Für alle postmenopausalen Frauen und für Männer ab dem 50. Lebensjahr mit einem klinischen Risikofaktor für eine Fraktur wird eine FRAX®-Analyse (https://frax.shef.ac.uk/FRAX/tool.aspx?lang=de) empfohlen [44]; *Evidenzgrad Ia*. Die Ergebnisse der FRAX®-Analyse entscheiden über das weitere Vorgehen und ggf. über die Notwendigkeit einer zusätzlichen DXA-Messung, wie im Kap. 6 „Frakturrisiko“ ausführlich beschrieben wird.

Ergänzend sollen bei älteren (> 70 Jahre) und sturzgefährdeten Patienten mit einem klinischen Risikofaktor für eine Fraktur die Muskelkraft überprüft und Koordinationstestungen durchgeführt werden. Zu den klassischen Funktionstests in diesem Setting zählen der Chair-Rising-Test, der Timed-up-and-Go-Test und der Tandemstand (s. Kap. 4 „Prävention und Basismaßnahmen“) [42]; *Evidenzgrad Ia.*

### 5.2 Bildgebende Verfahren

Empfehlungen:

- Eine radiologische Untersuchung der Brust- und Lendenwirbelsäule mittels Röntgen zur Detektion von Wirbelkörperfrakturen ist Teil einer osteologischen Abklärung; alternativ kann eine laterale Darstellung der Wirbelsäule mittels DXA (sog. vertebrales Frakturassessment) erfolgen; *starke Empfehlung*.
- Der alleinige radiologisch bildgebende Nachweis einer verminderten KMD und/oder eines erhöhten Frakturrisikos ist aufgrund vieler Faktoren nicht möglich; *bedingte Empfehlung*.

Nur etwa ein Viertel aller Wirbelkörperfrakturen wird als eindeutiges klinisches Ereignis (= klinisch vertebrale Fraktur) erkannt, da die Symptome oft leicht und unspezifisch sind. Im Gegensatz dazu werden Wirbelkörperfrakturen häufig als Zufallsbefund im Rahmen einer radiologischen Untersuchung erkannt. Das Vorhandensein einer nicht-traumatischen Wirbelkörperfraktur ist ein unbestreitbarer Beweis für eine verminderte Knochenstärke, d. h. für eine Osteoporose. Trotz der eindeutigen klinischen Bedeutung von Wirbelkörperfrakturen werden sie in der klinischen Praxis nach wie vor zu wenig diagnostiziert.

Der radiologische Nachweis einer verminderten KMD und Osteoporose ist subjektiv und hängt von der Aufnahmetechnik, der Ausrüstung und dem Körperbau des Patienten ab. In dem Bestreben, definierbare, reproduzierbare und objektive Methoden zur Erkennung von Wirbelkörperfrakturen zu entwickeln, wurden mehrere Methoden zur Diagnose und Einstufung des Schweregrads von Wirbelkörperfrakturen auf konventionellen Röntgenbildern der Wirbelsäule entwickelt und verfeinert. Alternative Methoden sind die DXA zur Beurteilung von Wirbelkörperfrakturen im Rahmen der VFA und die Computertomographie (CT). Diese Methoden können im Großen und Ganzen entweder als qualitativ, semiquantitativ (SQ) oder quantitativ (QM) betrachtet werden [142]; *Evidenzgrad Ia*.

Bei der SQ-Analyse werden die Röntgenbilder der Wirbelsäule von einem erfahrenen Radiologen ohne vorherige Messung der Wirbelhöhen ausgewertet. Der am häufigsten verwendete SQ-Ansatz ist der von Genant et al. [143, 144].

Osteoporotische Wirbelkörperfrakturen betreffen den Corpus vertebrae und damit nur die ventrale, kompressionsbelastete Säule und werden in Stufen nach Genant von 1 (leicht) bis 3 (schwer) eingeteilt; beginnende Frakturen sind definiert als eine Zunahme um eine Stufe oder mehr auf Nachfolgeröntgenbildern. Eine Wirbelkörperfraktur des Grades 1 (leicht) entspricht einer Verringerung der vorderen, mittleren und/oder hinteren Höhe um 20–25 % im Vergleich zur erfahrungsgemäß zu erwartenden Höhe des Wirbelkörpers. Grad 2 (mäßige) Wirbelkörperfraktur ist eine Reduktion der Wirbelhöhe um etwa 25–40 %, und Grad 3 (schwere) Wirbelkörperfraktur ist eine Reduktion der Wirbelhöhe um > 40 %. Zusätzlich werden andere morphologische Veränderungen wie Endplattenknickung oder -verkrümmung und Kortikalisfrakturen in die Diagnose einbezogen, insbesondere bei der Unterscheidung zwischen einer leichten Deckplattenimpressionsfraktur und einer Wirbelkörperdeformität ohne Fraktur [143, 144]; *Evidenzgrad Ia*.

Die einfache sagittale Rekonstruktion mit der Multidetektor-CT (MDCT) ermöglicht die Beurteilung der Wirbelsäule bei allen thorakalen oder abdominalen CT-Untersuchungen, die für nicht-spinale klinische Indikationen durchgeführt werden, was die zufällige Identifizierung von Wirbelkörperfrakturen ermöglicht [145]. Die sagittalen CT-Aufnahmen sollten ebenfalls routinemäßig untersucht werden, da sie in der Regel eine größere Länge der Wirbelsäule umfassen als die axialen Schnitte [146]; *Evidenzgrad Ia*. Die Haupteinschränkung für den weit verbreiteten primären und nicht zufälligen Einsatz der CT zur Diagnose von Wirbelkörperfrakturen ist die mangelnde Verfügbarkeit und die mit der CT verbundene Strahlendosis, obwohl sich Letztere mit modernen Techniken und Geräten verbessert hat.

Da sich osteoporotische Wirbelkörperfrakturen in der Regel schrittweise entwickeln und gelegentlich ein leichtes stufenweises Fortschreiten des Schweregrads aufweisen, sind eine hohe Sensitivität und Spezifität bei der Röntgenbeurteilung möglicherweise nicht erreichbar. Eine Wirbelkörperfraktur wird auf dem Röntgenbild diagnostiziert, wenn ein Verlust von mindestens 20 % der Wirbelhöhe oder eine sichtbare Kortikalis‑/Endplattenfraktur vorliegt. Bei diesem Ansatz wird eine beträchtliche Anzahl leichter Wirbelkörperfrakturen eindeutig übersehen.

Die Magnetresonanztomographie (MRT) kann zur Lösung dieses Problems beitragen, indem sie selbst bei den leichtesten akuten Wirbelkörperfrakturen ein Knochenmarködem nachweist. Ein Knochenmarködem ist bei fehlender Knochenmarkinfiltration ein sensitives und spezifisches Zeichen für eine akute oder subakute Wirbelkörperfraktur, selbst wenn nativ-radiologisch keine Fraktur sichtbar ist. Die MRT (inkl. Short-Tau-Inversion-Recovery-Sequenz [STIRS]) hilft daher bei der Unterscheidung zwischen rezenter osteoporotischer und maligner Wirbelkörperfraktur [147] (s. auch Abschn. 5.4. „Sonstige radiographische Verfahren“); *Evidenzgrad Ia*.

### 5.3 Knochenmineraldichte

Empfehlungen:

- Das Standardverfahren ist die DXA-Messung an der Lendenwirbelsäule und am linken Femur, ggf. ergänzt oder ersetzt durch die Messung des rechten Femurs und/oder unter bestimmten Umständen des distalen Radius des weniger belasteten Unterarms. Die Untersuchung inklusive Auswertung und die Interpretation der Messdaten sollen durch medizinisches Fachpersonal mit spezieller Ausbildung/Zertifizierung in der DXA-Interpretation und in Übereinstimmung mit den aktuellen Empfehlungen der ISCD erfolgen; *starke Empfehlung*.
- Die Ergebnisse der DXA-Untersuchung sollten zeitnah dem Zuweiser mitgeteilt werden; *starke Empfehlung*.
- Ergänzend soll die Beurteilung den TBS sowie klinische Risikofaktoren beinhalten; *bedingte Empfehlung*.
- Die Verwendung von QUS und QCT wird derzeit für die Diagnose von Osteoporose nicht empfohlen. FRAX® ist für diese Methoden nicht validiert; *starke Empfehlung*.

Die DXA ist eine zweidimensionale Bildgebungstechnologie, die entwickelt wurde, um die KMD des gesamten menschlichen Skeletts und auch spezifisch für Prädilektionsstellen osteoporotischer Frakturen zu messen. Um die Interpretation der KMD-Messergebnisse zu vereinfachen und eine Vergleichbarkeit zwischen verschiedenen DXA-Geräten zu ermöglichen, wurde das T‑Score-Konzept eingeführt. Bei diesem Konzept wird die KMD eines Individuums mit dem Mittelwert einer jungen, gesunden Referenzpopulation verglichen, wobei die Differenz als SD ausgedrückt wird. Die Hersteller sollten weiterhin National Health and Nutrition Examination Survey III (NHANES III)-Daten als Referenzstandard für die T‑Scores von Oberschenkelhals und Gesamthüfte verwenden. Die Hersteller sollten weiterhin ihre eigenen Datenbanken für die Lendenwirbelsäule als Referenzstandard für T‑Scores verwenden [148].

Seit den frühen 1990er-Jahren basieren die diagnostischen Kategorien „Normal“, „Osteopenie“ und „Osteoporose“, wie sie von einer WHO-Arbeitsgruppe empfohlen werden, auf dem Konzept der KMD-Messung [149]. Diese Kriterien dürfen jedoch nicht mit einem individuellen Frakturrisiko oder einer individuellen Therapieentscheidung gleichgesetzt werden.

Das Frakturrisiko nimmt mit abnehmender KMD progressiv zu. Systematische Übersichten und Metaanalysen bevölkerungsbezogener Beobachtungsstudien mit absorptiometrischen Verfahren zeigen, dass das Frakturrisiko für jede SD-Abnahme in der KMD um etwa das 2fache steigt [2, 45]; *Evidenzgrad Ia*. Der Gradient des Frakturrisikos variiert je nach Ort und verwendeter Technik, Alter der Person und Frakturtyp [45]; *Evidenzgrad Ia*. Der prädiktive Wert der KMD für hüftnahe Frakturen ist mindestens so gut wie der des Blutdrucks allein für Schlaganfälle [150]; *Evidenzgrad IV*.

Die WHO und die International Osteoporosis Foundation (IOF) empfehlen als Referenztechnologie für die Messung der KMD die DXA-Messung am Oberschenkelhals, da sie einen höheren Vorhersagewert für Frakturen hat [151, 152]; *Evidenzgrad Ia*. Die KMD kann an jeder der beiden Hüften gemessen werden. Die bilaterale Messung ist geeignet, um Daten für T‑Scores (oder Z‑Scores) zu generieren. Wenn beide Hüften gemessen wurden, sollte der niedrigste T‑Score (oder Z‑Score) des rechten oder linken Schenkelhalses oder der Gesamthüfte für die diagnostische Klassifikation herangezogen werden, nicht aber der mittlere T‑Score (oder Z‑Score). Wenn beide Hüften wiederholt gemessen werden, sollte die mittlere bilaterale totale Hüft-KMD zur Verlaufskontrolle herangezogen werden [153]. Die Ergebnisse der mittels DXA erhobenen Oberschenkelhals-KMD-Werte können im FRAX® eingegeben werden. Die Wirbelsäule ist aufgrund der hohen Prävalenz degenerativer Veränderungen, die den KMD-Wert durch Artefakte in der Messung erhöhen, nicht immer eine anatomische Lokalisation für die Risikobewertung oder für die Diagnose von Osteoporose bei älteren Menschen. Eine Messung bei einer älteren Person, die eine niedrige KMD aufweist, ist jedoch fast immer valide und klinisch nützlich, insbesondere bei Personen mit einer im Vergleich zur Hüfte unverhältnismäßig niedrigen KMD der Wirbelsäule.

Bei gleicher DXA-gemessener Schenkelhals-KMD haben Männer und Frauen in etwa das gleiche Frakturrisiko [46]; *Evidenzgrad IIa*. Daher ist der empfohlene Referenzbereich, aus dem die T‑Scores für Schenkelhals und Gesamthüfte für Männer, Frauen und Transgenderpersonen definiert werden, der aus dem NHANES III für weiße Frauen im Alter von 20 bis 29 Jahren abgeleitete Bereich [148].

Die Referenzbereiche, aus denen die T‑Scores für die Lendenwirbelsäule und den distalen Unterarm sowohl für Männer als auch für Frauen aller Ethnizitäten berechnet werden, sind in der Regel die des Herstellers des DXA-Scanners [148].

Osteoporose kann anhand des KMD-T-Scores diagnostiziert werden, der an der gesamten Hüfte, dem Oberschenkelhals oder der Lendenwirbelsäule gemessen wird. Die Vorhersage des Frakturrisikos wird jedoch durch die Verwendung von Messungen an mehreren Stellen nicht verbessert [154, 155]; *Evidenzgrad IIa*. Wenn eine KMD-Messung an der Hüfte aus technischen Gründen nicht möglich ist oder wenn die Wirbelsäule unterschiedlich betroffen ist, können KMD-Messungen (sofern verwendbar) an der Wirbelsäule zur Diagnose herangezogen werden. Die Diagnose einer Osteoporose kann auf der Grundlage des T‑Scores des distalen Unterarms (1/3 Radius) gestellt werden, wenn weder die Wirbelsäule noch die Hüfte zuverlässig gemessen oder interpretiert werden können oder wenn ein Patient die Gewichtsgrenze für die DXA-Tabelle überschreitet oder ein primärer Hyperparathyreoidismus vorliegt. Der T‑Score am Radius oder der Lendenwirbelsäule darf allerdings nicht im FRAX® verwendet werden [148]; *Evidenzgrad IV*. Anzumerken ist, dass die DXA in Österreich derzeit nicht als organisiertes Screeningverfahren vorgesehen ist.

Zu den speziellen DXA-Untersuchungstechniken gehören die Messung des distalen Radius, das VFA und damit zusammenhängend die eventuelle Feststellung einer Kalzifikation der Aortenwand (falsch hohe Werte) sowie die Body Composition.

Das VFA kann zum Nachweis einer Wirbelkörperfraktur verwendet werden und korreliert gut mit der Radiographie [156]. Bei Verdacht auf eine frische Wirbelkörperfraktur ist es jedoch im Vergleich zu Radiographie, CT, MRT oder nuklearmedizinischen Methoden zu ungenau, um Frakturen sicher ausschließen zu können.

Die Body Composition kann sinnvoll eingesetzt werden, um die mit einer Osteoporose oft assoziierte verminderte Muskelmasse quantitativ zu erfassen, wie es bei Anorexia nervosa, anderen Formen der chronischen Mangelernährung oder im höheren Lebensalter bei der Sarkopenie typisch ist. Auch bei Adipositas, v. a. vor bariatrisch-chirurgischen Eingriffen, ist die DXA in die entsprechende Leitlinie aufgenommen worden und soll auch postoperativ zur Kontrolle des Therapieerfolges oder der möglichen Veränderungen durchgeführt werden. Obwohl die Evidenz für das Outcome bei diesen Szenarien nicht gesichert ist, wird die DXA als das beste aller dafür verfügbaren Verfahren angesehen.

Die für präventive oder therapeutische Entscheidungen relevanten Parameter sind die Magermasse des Gesamtkörpers (Total Lean Body Mass, entspricht weitgehend der Muskulatur), die Fettmasse des Gesamtkörpers (Total Fat Mass), der prozentuelle Fettgehalt des Ganzkörpers, das Gesamtkörpergewicht (Unterschiede zum mit der Körperwaage ermittelten Wert sind wegen des unterschiedlichen Gehalts an Körperwasser oder der unterschiedlichen Methodik möglich) und das viszerale Fettgewebe (Visceral Adipose Tissue [VAT]), quantifiziert anhand der Visceral Fat Area in cm^2^.

Die serielle Messung der KMD kann zur Überwachung des Ansprechens auf die Behandlung verwendet werden (s. Abschn. 9.2. „Therapiemonitoring“) [157]. Die KMD der Lendenwirbelsäule zeigt die größten behandlungsbedingten Veränderungen und ist die bevorzugte Messstelle, obwohl bei ausgeprägten degenerativen Veränderungen der Wirbelsäule die KMD an der Hüfte eine bessere Messstelle ist.

Die Gültigkeit von KMD-Messungen hängt von einer guten Qualitätskontrolle ab. Internationale Gremien (International Society for Clinical Densitometry, Royal Osteoporosis Society UK) haben Standards für die Berichterstattung über DXA-Scans veröffentlicht. Der allgemein verwendete Parameter „Least Significant Change“ (LSC) ist definiert als der geringste Betrag der Veränderung zwischen 2 Messungen im Laufe der Zeit, der überschritten werden muss, bevor eine Veränderung als signifikant angesehen werden kann (mit 95 % Sicherheit). Die LSC findet klinische Anwendung bei der Überwachung des Krankheitsverlaufs oder der Behandlungseffekte bei der KMD und dem Knochenmineralgehalt. Konsekutive DXA-Messungen müssen daher den gerätespezifischen LSC angeben und auch darauf hinweisen, dass eine Veränderung als signifikant einzustufen ist. In klinischer Praxis ist in so einem Fall die prozentuelle Veränderung mit einem „*“ versehen [148, 158].

Die mit quantitativer CT (QCT) gemessene flächige KMD des Oberschenkelhalses sagt osteoporotische Frakturen bei Männern und Frauen voraus und ist gleichwertig mit der von der DXA abgeleiteten flächigen KMD [159–161]. Die aus zweidimensionalen Projektionen von QCT-Daten berechneten T‑Scores für den Schenkelhals und die gesamte Hüfte entsprechen den entsprechenden T‑Scores, die mit der DXA ermittelt wurden. Daher können KMD-Messungen der Schenkelhals-CT-Röntgenabsorptiometrie (CTXA) in FRAX® aufgenommen werden [148, 162–164] (s. Abschn. 6.3. „Interventionsschwellen“); *Evidenzgrad IIa*. Andere Verfahren zur Bewertung der skeletalen KMD, einschließlich Untersuchung mittels quantitativen Ultraschalls (QUS), sind weniger gut validiert als absorptiometrische Verfahren.

Bei divergenten Messergebnissen zwischen der KMD am Schenkelhals und an der Lendenwirbelsäule oder einem stark verminderten Trabecular Bone Score (TBS) gegenüber einer eventuell osteopenen KMD kann das FRAX®-Ergebnis korrigiert werden. Die Tab. 5 erläutert näherungsweise Anpassungen und Überlegungen zu Wahrscheinlichkeiten von MOF und hüftnahen Frakturen zur Unterstützung der Interpretation von FRAX®.

**Tab. 5:** Näherungsweise Anpassungen und Überlegungen zu Wahrscheinlichkeiten von MOF und hüftnahe Frakturen zur Unterstützung der Interpretation von FRAX®

Risikovariable	Anpassung an FRAX®	Literatur
Gleichzeitige Daten zur KMD der Lendenwirbelsäule (L1–L4)	Erhöhung/Verringerung der MOF-Wahrscheinlichkeit um 10 % für jede gerundete T‑Score-Differenz zwischen Lendenwirbelsäule und Schenkelhals^a^	[165, 166]
Trabecular Bone Score	Erhöhung der MOF-Wahrscheinlichkeit um 30 % für jede SD des TBS	[167]
Hüftachsenlänge	Erhöhung der Wahrscheinlichkeit von hüftnahen Frakturen um 30 % für jede SD-Erhöhung der HAL	[168]

*FRAX* Fracture Risk Assessment Tool, *HAL* Hüftachsenlänge, *KMD* Knochenmineraldichte, *L* Lendenwirbel, *MOF* „major osteoporotic fracture“ (hüftnahe Fraktur, klinisch vertebrale Fraktur, Unterarmfraktur, Humerusfraktur), *SD* Standardabweichung, *TBS* Trabecular Bone Score

^a^ Eine Anpassung der FRAX®-Wahrscheinlichkeiten nach unten sollte nur im Zusammenhang mit einer sehr zuverlässigen KMD-Messung der Lendenwirbelsäule vorgenommen werden und nicht auf der Grundlage eines nicht übereinstimmenden Ergebnisses durch ein Artefakt (z. B. eine degenerative Veränderung an der Lendenwirbelsäule)

### 5.4 Sonstige radiographische Verfahren

Empfehlungen:

- Die Radiographie (konventionelle Röntgenaufnahmen) ist der Standard zum Nachweis von Wirbelkörperfrakturen und als Surrogatmarker anerkannt. Wirbelkörperfrakturen ohne akutes Trauma sollen entsprechend der Einschätzung nach Genant semiquantitativ erfasst werden; *starke Empfehlung*.
- Zufallsbefunde von Wirbelkörperfrakturen (morphometrische Wirbelkörperfrakturen) im Rahmen einer aus anderen Gründen durchgeführten radiologischen Untersuchung sollen im Befund dokumentiert werden; *bedingte Empfehlung*.

#### *Radiographie, Computertomographie und Magnetresonanztomographie:*

Die Indikation zum Nachweis einer osteoporotischen Wirbelkörperfraktur ist bei akuten, starken, umschriebenen und neu aufgetretenen oder über Tage anhaltenden Rückenschmerzen oder bei bisher nicht abgeklärten chronischen Rückenschmerzen im Rahmen der Verdachtszeichen des Red-Flag-Konzepts gegeben (= klinisch vertebrale Fraktur). Weitere klinische Verdachtszeichen sind eine Reduktion der Körpergröße um ≥ 4 cm bei Verlaufsuntersuchungen, ein auffälliger Befund bei der klinischen Untersuchung, anamnestisch erhobene frühere Frakturen, ein hoher FRAX®-Wert oder der Befund einer niedrigen KMD.

Das opportunistische Screening, d. h. in diesem Zusammenhang das Erfassen von osteoporotischen Veränderungen als Zufallsbefund bei radiologischen Untersuchungen, v. a. auf Thoraxröntgenaufnahmen und bei CT-Aufnahmen des Körperstamms, nimmt angesichts der zahlreichen radiologischen und nuklearmedizinischen Untersuchungen einen immer höheren Stellenwert ein [169–171]. Wirbelkörperfrakturen und andere mit einer metabolischen Knochenerkrankung assoziierte Veränderungen sollen daher bei der radiologischen Befundung systematisch erfasst und dokumentiert werden.

Die radiographische Untersuchung soll an der Brust- und Lendenwirbelsäule durchgeführt werden. Die Einschätzung der Wirbel soll gemäß der semiquantitativen Methode nach Genant [143, 144] (s. auch Abschn. 5.2. „Bildgebende Verfahren“) durch subjektive Beurteilung durchgeführt werden. Ist die radiographische Beurteilung nicht aussagekräftig, soll in weiterer Folge eine CT oder eine MRT durchgeführt werden. Um nachzuweisen, ob ein Wirbeleinbruch rezent ist, soll die Beurteilung mit einer MRT kombiniert werden. Bei der CT-Untersuchung ist ein ausreichend hoher Rekonstruktionsalgorithmus nötig, um die Knochen mit beurteilbarer Ortsauflösung darzustellen. Durch technologische Entwicklungen, v. a. bei den Detektoren und neuerdings mit dem Photon-Counting-CT, ist die Strahlenexposition einer einzelnen Untersuchung deutlich geringer, sodass für Gewebe mit hohen Kontrasten Low-Dose- und zunehmend auch Ultra-Low-Dose-Techniken durchführbar sind. Mit der Dual-Energy-CT kann, falls verfügbar, ein Knochenmarködem auf diese Weise dargestellt werden.

Für die MRT-Untersuchung ist standardmäßig neben T1- und T2-gewichteten Spin-Echo-Sequenzen auf jeden Fall eine T2-gewichtete Sequenz in sagittaler Ebene mit Fettunterdrückung und für die Lendenwirbelsäule neuerdings auch eine koronare Sequenz durchzuführen.

Im Befund sollen Wirbelkörperfrakturen und andere mit einer metabolischen Knochenerkrankung assoziierte Veränderungen systematisch erfasst und dokumentiert werden. Dies inkludiert auch die bei Brust- und Lendenwirbelsäuleaufnahmen mitabgebildeten Rippen und das Os sacrum. Es sollen die Zahl und die Höhe der frakturierten Wirbel mit Einschätzung nach Genant angegeben werden, und bei Verlaufskontrollen soll auf eine etwaige Progression des Frakturgeschehens eingegangen werden. Die Genant-Klassifikation wurde mittlerweile auch von der Arbeitsgemeinschaft für Osteosynthesefragen (AO) in ihre aktuelle Klassifikation von Wirbelsäulenfrakturen integriert.

Alle Formen der Höhenreduktion eines Wirbels (veraltet Sinterung genannt), auch Wirbeldeformitäten, sollen erwähnt werden. Unter einer Wirbeldeformität versteht man eine Höhenreduktion der Wirbelkörper im gesamten Bereich zwischen viertem Thorakal- und viertem Lumbalwirbel um < 20 % oder eine Deckplattenimpression bzw. Formveränderungen aus anderer Ursache (wie Morbus Scheuermann, gewölbeförmig-überlastungsbedingte Deckplattendeformierung, atraumatische Schmorl-Knorpelhernien und an der mittleren Brustwirbelsäule zu beobachtende unspezifische, sich über mehrere Segmente erstreckende geringe Keilwirbelbildungen).

Frakturen sind so zu analysieren, dass eine differenzialdiagnostische Abgrenzung zu nicht-osteoporotischen Frakturen – insbesondere pathologischen Frakturen infolge eines malignen Geschehens – möglich ist. Neben Wirbelkörperfrakturen sind auch auf eine Osteoporose hinweisende Veränderungen der Knochenarchitektur zu beschreiben und bei schwerem Ausprägungsgrad in der Diagnose zu erwähnen. Konkret zu nennen sind eine Verschmälerung der Kortikalis (Rahmen- oder Geisterwirbel), eine stärkere Ausprägung der vertikalen gegenüber den horizontalen Trabekeln oder eine durch die Fraktur bedingte Fehlstellung wie eine anguläre Hyperkyphose. Schließlich sind etwaige Veränderungen durch eine sekundäre Osteoporose zu dokumentieren. Obligat zu befunden sind auch arterielle Gefäßverkalkungen, da die radiologisch identifizierte Arteriosklerose ein unabhängiger Risikofaktor für kardiovaskuläre Erkrankungen darstellt. Zur Graduierung kann der Abdominal Aortic Calcification Score (AAC-8) herangezogen werden (dieser ist 0, wenn keine Verkalkung vorliegt, < 3 oder „gering“ bei geringer Verkalkung an der Aortenvorder- oder -hinterwand oder ≥ 3 „ausgeprägt“ und dann mit einem erhöhten Risiko für kardiovaskuläre Erkrankungen assoziiert). Veränderungen wie eine seit einer Voruntersuchung aufgetretene starke Verschlechterung der KMD, die einer raschen Therapieentscheidung bedürfen, sollen im Befundtext hervorgehoben werden.

Mit der MRT kann als wichtige Zusatzinformation das etwaige Vorliegen eines bandförmigen, meist subchondralen Knochenmarködems als Hinweis auf eine rezente Wirbelkörperfraktur – die als Mikrofraktur auch in einem makroskopisch in seiner Form erhaltenen Wirbel beobachtet werden kann – dokumentiert werden. Für eine orthopädisch-traumatologische Versorgung sind daher Wirbelkörperfrakturen nach der Arbeitsgruppe Wirbelsäule der Deutschen Gesellschaft für Orthopädie und Unfallchirurgie (DGOU) und ergänzend zur AO-Klassifikation zu unterteilen [172]. Dabei wird als Grad 1 ein Knochenmarködem bei erhaltener Form des Wirbelkörpers definiert, als Grad 2 eine Impressionsfraktur, als Grad 3 und 4 eine inkomplette oder komplette Berstungsfraktur und als Grad 5 eine Rotations- oder Distraktionsverletzung.

#### *Trabecular Bone Score:*

Der TBS, ein aus DXA-Bildern der Lendenwirbelsäule abgeleitetes Maß für die Graustufenstruktur, verbessert die Vorhersage des Frakturrisikos über das hinaus, was die Kombination aus KMD durch DXA und klinischen Risikofaktoren bietet. Die meisten Querschnittsstudien haben gezeigt, dass ein niedriger TBS mit Wirbelkörper‑, Hüft- und anderen osteoporotischen Frakturen bei älteren Frauen und Männern verbunden ist. Es gibt konsistente klinische Hinweise darauf, dass der TBS ein Prädiktor für das Frakturrisiko bei postmenopausalen Frauen und älteren Männern ist.

Die Auswirkungen verschiedener antiresorptiver Therapien auf den TBS wurden hauptsächlich bei postmenopausalen Frauen und bei Frauen mit einem durch die Behandlung von Brustkrebs verursachten Knochenverlust untersucht. Der TBS wird klinisch an der Lendenwirbelsäule mit einer speziellen Software gemessen, die dieselbe Region (L1–L4) verwendet wie eine herkömmliche KMD-Messung.

Es gibt Hinweise darauf, dass der TBS nicht durch das Vorhandensein von degenerativen Veränderungen des Knochens beeinträchtigt wird, die die KMD der Lendenwirbelsäule in der DXA-Messung in der Regel überbewertet. Im Gegensatz dazu wird der TBS durch übermäßiges Weichteilgewebe (Adipositas) im Bauchraum beeinträchtigt, welches die Bildtextur verschlechtert und die TBS-Werte verringert. Die Software ist für einen BMI von 15–37 kg/m^2^ validiert. Eine neue Version aus dem Jahr 2023 soll diese Problematik vermindern. Es besteht kein Konsens darüber, was ein normaler und was ein abnormaler TBS ist. Der TBS-Hersteller empfiehlt, dass TBS-Werte ≥ 1,350 als normal gelten, während TBS zwischen 1,200 und 1,350 mit „teilweise verschlechterter“ trabekulärer Mikroarchitektur übereinstimmt und TBS ≤ 1,200 auf „abgebauten/verschlechterten“ Knochen hinweist.

Leitlinien der International Society of Clinical Densitometry (ISCD) und der European Society for Clinical and Economic Aspects of Osteoporosis, Osteoarthritis and Musculoskeletal Diseases (ESCEO) unterstützen den Einsatz des TBS zur Bewertung des Frakturrisikos bei postmenopausalen Frauen und bei Männern ≥ 50 Jahre. Darüber hinaus wurde der TBS für den Einsatz bei postmenopausalen Frauen mit Diabetes mellitus Typ 2 zur Vorhersage eines Frakturrisikos vorgeschlagen [173, 174]; *Evidenzgrad Ia*.

Der Online-Rechner auf der FRAX®-Website (https://frax.shef.ac.uk/FRAX/tool.aspx?lang=de) verfügt über die Option „Anpassung mit TBS“, wenn KMD des Schenkelhalses in den Rechner eingegeben wird und TBS verfügbar ist. Es erfolgt eine TBS-bereinigte 10-Jahres-Frakturwahrscheinlichkeit für MOF und hüftnahe Fraktur. Diese Anpassung enthält einen Interaktionsterm „TBS × Alter“, der die mit zunehmendem Alter abnehmende Stärke der TBS-Anpassung bei FRAX® widerspiegelt. Im Allgemeinen hat die Verwendung des TBS zur Anpassung des FRAX®-Scores eine größere klinische Auswirkung auf jene Patienten, die sich nahe an der Interventionsschwelle befinden, wenn das Risiko anhand der FRAX® ohne TBS bestimmt wird.

Angesichts der wesentlich geringeren Veränderungen des TBS im Vergleich zu den KMD-Werten bei Patienten, die mit Bisphosphonaten behandelt werden, und aufgrund des Fehlens von Studien, die belegen, dass behandlungsbedingte Veränderungen der TBS mit dem Frakturrisiko in Zusammenhang stehen, wird derzeit empfohlen, den TBS nicht zur Überwachung von Patienten unter Bisphosphonat-Therapie einzusetzen [173]. Die Rolle des TBS bei der Überwachung von Patienten, die mit Teriparatid, Denosumab oder neueren Osteoporosemedikamenten behandelt werden, bleibt derzeit ungewiss; *Evidenzgrad IV*.

#### *Quantitativer Ultraschall:*

QUS-Geräte arbeiten mit hochfrequenten Schallwellen im Ultraschallbereich, typischerweise zwischen 0,1 und 1,0 Megahertz (MHz), die von piezoelektrischen Wandlern erzeugt und erfasst werden. Die technischen Unterschiede zwischen den QUS-Systemen sind beträchtlich: Die Frequenzen variieren, und die Schallköpfe sind unterschiedlich groß.

Der Kalkaneus ist die am häufigsten untersuchte Stelle des Skeletts, obwohl auch andere Knochen wie Radius (Speiche), Tibia (Schienbein) oder Fingerknochen verwendet werden können. QUS-Geräte messen in der Regel die Schallgeschwindigkeit („speed of sound“ [SOS]) und die Breitbandultraschalldämpfung (BUA); anschließend können proprietäre Werte wie der „quantitative Ultraschallindex“ (QUI) oder der „Steifigkeitsindex“ berechnet und angegeben werden. Aus Ultraschallparametern berechnete Werte können zur Erstellung einer geschätzten KMD und eines T‑Scores verwendet werden. Ein QUS-T-Score ist nicht dasselbe wie ein DXA-T-Score, da unterschiedliche Knocheneigenschaften gemessen und unterschiedliche Referenzdatenbanken verwendet werden; daher können die von QUS abgeleiteten T‑Scores nicht mit der WHO-Klassifikation verwendet werden.

Die einzige mit QUS validierte anatomische Lokalisation für das Management von Patienten mit Osteoporose ist der Kalkaneus [175]. QUS-Messungen können nicht zur Diagnose von Osteoporose gemäß den WHO-Kriterien und zur Überwachung der Auswirkungen einer Osteoporosebehandlung verwendet werden [175]; *Evidenzgrad Ia*.

#### *Quantitative Computertomographie der Wirbelsäule und Hüfte:*

Die CT ist ein röntgenbasiertes Verfahren, das eine räumliche Verteilung des Röntgenabsorptionskoeffizienten liefert, der nach Normierung auf die Absorption von Wasser und Luft als CT-Wert definiert und in Hounsfield-Einheiten (HU) gemessen wird. Für die KMD-Analyse an der Wirbelsäule werden die Volumina von L1 + L2 empfohlen. Für die QCT der Hüfte sollte die Kombination aus Oberschenkelhals, Trochanter und Intertrochanter untersucht werden.

Die CTXA ist eine Technik zur Simulation von Projektionsbildern des DXA-Typs aus QCT-Daten für die Messung von flächiger KMD an der Hüfte (s. Abschn. 5.3. „Knochenmineraldichte“). Die QCT-Präzisionsfehler sind mit denen der DXA vergleichbar [176]; *Evidenzgrad Ia*.

Im Gegensatz zur DXA liefert die QCT regionale dreidimensionale (3D) Verteilungen der KMD. Unterschiedliche KMD-Effekte in der kortikalen, subkortikalen und trabekulären KMD verbessern das Verständnis der Pathophysiologie verschiedener antiresorptiver Behandlungen. Zusammen mit Messungen der kortikalen Dicke und des Knochenvolumens zur potenziellen Identifizierung der Periostanlagerung wird somit ein viel detaillierteres Verständnis der Behandlungseffekte erreicht. Die QCT-Messung wird weniger durch degenerative Veränderungen an der Wirbelsäule beeinträchtigt, insbesondere wenn die trabekuläre KMD gemessen wird. Mittels QCT gemessene T‑Scores können nicht in FRAX® eingesetzt werden.

#### *High-ResolutionPeripheral Quantitative Computed Tomography:*

Die High-Resolution Peripheral QCT (HR-pQCT) ist eine spezielle QCT-Technik zur Beurteilung der KMD und der trabekulären und kortikalen Knochenstruktur an peripheren Stellen wie dem distalen Radius und der distalen Tibia. Kortikale und trabekuläre Regionen können mit halbautomatischen oder automatischen Methoden segmentiert werden [177, 178]. Volumetrische (v)KMD in mg/cm^3^ kann für den gesamten Knochen im Scanbereich und für einzelne Regionen bestimmt werden. Zusätzlich zur vKMD kann eine morphometrische Analyse verwendet werden, um die Mikrostruktur des trabekulären Netzwerks zu bewerten.

Die durchschnittliche Trabekeldicke (Tb.Th) und der Abstand (Tb.Sp) werden aus dem Knochenvolumenanteil (BV/TV) und der durchschnittlichen Anzahl der Trabekel (Tb.N) abgeleitet. Das trabekuläre Volumen (BV/TV) wird aus der KMD des trabekulären Kompartiments bestimmt, indem eine Dichte von 1200 mg/cm^3^ für vollständig mineralisierten Knochen angenommen wird. Die Software des Scannerherstellers liefert verschiedene Messungen der Kortikalisdicke (Ct.Th). Ein Wert wird als Volumen des kortikalen Knochenvolumens geteilt durch die äußere Knochenoberfläche ermittelt. Der andere Wert wird direkt mithilfe von Distanztransformationsmethoden gemessen. Die kortikale Porosität (Ct.Po) wird als prozentualer Anteil der Hohlraumvoxel im Kortex berechnet.

Derzeit wird die HR-pQCT v. a. als Forschungsinstrument eingesetzt. Die Verfügbarkeit von HR-pQCT-Geräten ist immer noch begrenzt. Der zusätzliche Nutzen der Bewertung der trabekulären Architektur des distalen Radius für die Vorhersage des Frakturrisikos muss noch nachgewiesen werden, da altersbedingte Veränderungen an der Wirbelsäule stärker ausgeprägt sind.

Die Erhöhung der kortikalen Porosität scheint bei Patienten mit Diabetes mellitus Typ 2 von Bedeutung zu sein, die trotz normaler planarer KMD und erhöhter trabekulärer KMD ein höheres Frakturrisiko haben als Kontrollpersonen. Wie die HR-pQCT zeigt, haben diese Patienten eine verringerte kortikale volumetrische KMD und, im Falle von Fragilitätsfrakturen, eine erhöhte kortikale Porosität [179, 180]; *Evidenzgrad Ib*. Longitudinale kortikale und trabekuläre Veränderungen unter antiresorptiver oder anaboler medikamentöser Therapie konnten mit dieser Methode nicht-invasiv nachgewiesen werden [181]; *Evidenzgrad Ia*.

### 5.5 Osteologisches Labor

Empfehlungen:

- Bei allen Patienten mit Verdacht auf Osteoporose sollten die in der Folge dargestellten Basislaboruntersuchungen durchgeführt werden; *starke Empfehlung*.
- Diese Untersuchungen werden empfohlen, da sie dazu beitragen, sekundäre Osteoporoseformen zu identifizieren bzw. die Osteoporose von anderen Knochenerkrankungen abzugrenzen und ggf. Kontraindikationen für bestimmte Osteoporosetherapien aufzuzeigen; *starke Empfehlung*.
- Die Bestimmung von Knochenumbaumarkern wird empfohlen, wenn in bestimmten Situationen relevante Informationen erforderlich sind; *starke Empfehlung*.

Zusätzlich zur Anamnese und der klinischen Untersuchung sind bei Patienten mit Verdacht auf Osteoporose Laboruntersuchungen notwendig. Diese Untersuchungen tragen dazu bei, sekundäre Osteoporoseformen zu identifizieren [182, 183]; *Evidenzgrad IIIb*; bzw. die Osteoporose von anderen Knochenerkrankungen abzugrenzen. Darüber hinaus können diese Analysen eventuelle Kontraindikationen (z. B. Niereninsuffizienz, Hypokalziämie) gegen bestimmte, in der Osteoporosetherapie eingesetzte Medikamente, aufzeigen.

Bei allen Patienten mit Verdacht auf Osteoporose ist die Durchführung einer Laborbasisdiagnostik angezeigt. Als „*Basislabor*“ werden die Untersuchungen in Tab. 6 empfohlen.

**Tab. 6:** Laborparameter für die Basisdiagnostik der Osteoporose

Serum-Kalzium (gegebenenfalls ionisiertes Kalzium)
Serum-Albumin
Serum-Phosphat
Serum-alkalische Phosphatase
Serum-Gamma-Glutamyl-Transferase
eGFR
Komplettes Blutbild
Blutkörpersenkungsgeschwindigkeit oder C‑reaktives Protein
Serum-Eiweißelektrophorese
Serum-25-Hydroxy-Vitamin D
Serum-TSH

*eGFR* geschätzte glomeruläre Filtrationsrate, *TSH* Thyreoidea-stimulierendes Hormon

*Weitere Laboruntersuchungen* können entsprechend der Klinik und/oder eventuellen Auffälligkeiten im Basislabor angezeigt sein. Beispiele für diese Laboruntersuchungen sind: Serum-Transaminasen, Serum-Immunglobuline, Harn-Kalziumausscheidung, Parathormon, Serum-Testosteron, Sex-Hormone-Binding-Globulin, Knochenumbaumarker (z. B. Osteocalcin, Prokollagen Typ 1 N-terminales Propeptid [P1NP], „C-terminal crosslinking telopeptides of type I collagen“ [CTX]). Die Analyse des Vitamin-D-Status ist sehr gut mit 25-Hydroxy-Vitamin D möglich, die Bestimmung von 1,25-Hydroxy-Vitamin D sollte lediglich in speziellen Situationen erfolgen.

*Knochenumbaumarker* können entweder die Knochenformation (z. B. P1NP) oder den Knochenabbau (z. B. CTX) reflektieren. Die Bestimmung der Marker hat sich unter Studienbedingungen sehr gut bewährt. In der klinischen Praxis kann deren Anwendbarkeit allerdings aufgrund von präanalytischer und analytischer Variabilität eingeschränkt sein. Knochenumbaumarker müssen daher nicht bei allen Osteoporosepatienten bestimmt werden. Wie oben erwähnt, repräsentieren die Marker entweder die Knochenformation oder den Knochenabbau; Veränderungen der Marker werden jedoch auch bei nicht-osteoporotischen Knochenerkrankungen beobachtet, sodass diese nicht zur Diagnose der Osteoporose verwendet werden können [184].

In bestimmten Situationen kann der Einsatz der Marker jedoch gerechtfertigt sein. In Metaanalysen wurde gezeigt, dass höhere Spiegel der Umbaumarker ein höheres Frakturrisiko vorhersagen können [183, 185, 186]; *Evidenzgrad IIa*. Knochenumbaumarker (CTX oder P1NP) können auch zur Therapieüberwachung eingesetzt werden (s. auch Abschn. 9.2. „Therapiemonitoring“); bei der Gabe von Antiresorptiva zeigen die Marker bereits nach 3 bis 6 Monaten eine signifikante Verminderung, während diese bei knochenanabolen Therapien deutlich ansteigen [183, 187–190]; *Evidenzgrad Ia*. Nach Absetzen einer antiresorptiven Therapie wird ein Ansteigen der Knochenumbaumarker beobachtet. Entsprechend der Leitlinie der National Osteoporosis Guideline Group (NOGG) wird ein CTX-Monitoring nach dem Absetzen von Denosumab empfohlen (s. Abschn. 8.6.3.2. „Spezifische Osteoporosetherapie – Denosumab“) [44]; *Evidenzgrad IV*.

### 5.6 Knochenbiopsien

Empfehlungen:

- Bei Verdacht auf bestimmte systemische Knochenerkrankungen sollten die Biopsien am Beckenkamm entnommen und analysiert werden; *bedingte Empfehlung*.

Obwohl in der klinischen Praxis heute nur mehr selten Knochenbiopsien durchgeführt werden, sollte diese Technik auch im Rahmen dieser Leitlinie erwähnt werden. Bei Verdacht auf systemische Knochenerkrankungen werden die Biopsien am Beckenkamm entnommen; eine vorangehende „Markierung“ mit einem Tetrazyklinpräparat und die unentkalkte Aufarbeitung des Biopsates ermöglichen die Durchführung der dynamischen Histomorphometrie. Diese Technik gibt Informationen über die Struktur, die Mineralisation und die Umbauaktivitäten des Knochens.

Beispiele für Indikationen zu einer Knochenbiopsie sind unter anderem das Auftreten von Frakturen unter ungewöhnlichen Umständen, der Verdacht auf Osteomalazie (wenn die Labortests nicht schlüssig sind) oder die renale Osteodystrophie; *Evidenzgrad IV*. Nach der ÖGKM/ÖGPMR/ÖGN-Leitlinie [77] sollte bei Patienten mit chronischer Niereninsuffizienz in Einzelfällen, insbesondere bei chronischer Nierenerkrankung (CKD) Grad 5 (geschätzte glomeruläre Filtrationsrate [eGFR] < 15 ml/min/1,73 m^2^) oder CKD Grad 5D (Dialyse), eine Knochenbiopsie vor Therapieeinleitung erwogen werden [191].

## 6 Frakturrisiko

Die Klinik der Osteoporose ist unspezifisch. Im Sinne einer Prävention vor der ersten Fraktur ist die Evaluation des individuellen Frakturrisikos und eine möglicherweise daraus resultierende Behandlungsindikation ein wichtiges therapeutisches Ziel.

### 6.1 Risikomodelle – Ermittlung des Frakturrisikos

Empfehlungen:

- Eine FRAX®-Bewertung sollte bei jeder postmenopausalen Frau oder jedem Mann im Alter von ≥ 50 Jahren mit einem klinischen Risikofaktor für Fragilitätsfrakturen durchgeführt werden, um ggf. eine KMD-Messung am Schenkelhals mittels DXA zu initiieren und eine rechtzeitige Überweisung und/oder medikamentöse Behandlung einzuleiten; *starke Empfehlung*.
- Bei der Verwendung von FRAX® zur Berechnung der Frakturwahrscheinlichkeit sollte eine klinische Beurteilung erfolgen, wenn das klinische Risiko jene Faktoren übersteigt, die in FRAX® eingegeben werden können; *starke Empfehlung*.
- Arithmetische Anpassungen der FRAX®-Wahrscheinlichkeiten für MOF (hüftnahe Fraktur, klinisch vertebrale Fraktur, Unterarmfraktur, Humerusfraktur) sowie die eigenständige FRAX®-Auswertung für hüftnahe Frakturen können in der klinischen Praxis verwendet werden, um zusätzliche klinische Risikofaktoren zu berücksichtigen; *bedingte Empfehlung*.
- Die Untersuchung auf Wirbelkörperfrakturen (seitliches Röntgen der Brust- und/oder Lendenwirbelsäule, VFA im Rahmen einer DXA-Messung) ist bei postmenopausalen Frauen und Männern im Alter von ≥ 50 Jahren indiziert, wenn in der Anamnese ein Höhenverlust von ≥ 4 cm, eine Kyphose, eine kürzliche oder derzeitige systemische Glukokortikoidtherapie, andere mit einem erhöhten Risiko für vertebrale Frakturen assoziierte Faktoren oder akut auftretende Rückenschmerzen mit Risikofaktoren für Osteoporose vorliegen; *starke Empfehlung*.
- Die Ergebnisse der DXA-Untersuchung sollten dem Patienten zeitnah nach der Untersuchung von medizinischem Fachpersonal mit spezieller Ausbildung/Zertifizierung in der DXA-Interpretation in Übereinstimmung mit nationalen und internationalen Diagnose- und Berichtsstandards mitgeteilt werden; *starke Empfehlung*.
- Patienten mit Osteoporose und/oder einer Fragilitätsfraktur (= absolute Behandlungsindikation unabhängig von T‑Score) sollten auf zugrunde liegende Ursachen hin untersucht werden, wozu auch die Notwendigkeit von Basislaboruntersuchungen gehört; *starke Empfehlung*.
- Die Verwendung von QUS wird für die Diagnose von Osteoporose nicht empfohlen; *starke Empfehlung*.

#### 6.1.1 Instrumente zur Bewertung des Frakturrisikos

Die IOF und die WHO empfehlen, das Frakturrisiko als absolutes Risiko auszudrücken, d. h. als Wahrscheinlichkeit über einen Zeitraum von 10 Jahren [22]. Das absolute Frakturrisiko hängt vom Alter und der Lebenserwartung sowie vom aktuellen relativen Risiko ab. Der Zeitraum von 10 Jahren deckt die wahrscheinliche anfängliche Dauer der Behandlung und die Vorteile ab, die sich bei Abbruch der Behandlung fortsetzen können. Kürzere Zeithorizonte (z. B. 1, 2 oder 5 Jahre) sind für die Einstufung des Risikos nicht hilfreich [192, 193].

Algorithmen, die die Gewichtung der klinischen Risikofaktoren für das Frakturrisiko mit oder ohne KMD integrieren, wurden 2008 vom damaligen WHO-Kollaborationszentrum für metabolische Knochenerkrankungen in Sheffield entwickelt. Das *FRAX®-Tool* (https://frax.shef.ac.uk/FRAX/tool.aspx?lang=de) berechnet die 10-Jahres-Wahrscheinlichkeit einer hüftnahen Fraktur und/oder einer MOF (hüftnahe Fraktur, klinisch vertebrale Fraktur, Unterarmfraktur, Humerusfraktur). Das Instrument wurde in unabhängigen Kohorten extern validiert [23, 194]; *Evidenzgrad Ia*.

FRAX® basiert auf länderspezifischen Frakturdaten. Die österreichischen Kohorten wurden im Jahr 2022 aktualisiert und validiert [195].

*QFracture* basiert auf einer britischen prospektiven offenen Kohortenstudie mit routinemäßig erhobenen Daten aus Allgemeinarztpraxen, die zahlreiche klinische Risikofaktoren berücksichtigt und die kumulative 1‑ bis 10-Jahres-Inzidenz von hüftnahen Frakturen und/oder MOF schätzt (http://www.qfracture.org) [196].

Das National Institute for Health and Care Excellence (NICE) hat die Verwendung von Instrumenten zur Bewertung des Frakturrisikos (FRAX® oder QFracture) bei der Bewertung von Patienten empfohlen [4]. Da FRAX® und QFracture unterschiedliche Ergebnisse liefern (Frakturwahrscheinlichkeit unter Berücksichtigung des Mortalitätsrisikos im Falle von FRAX® und ein kumulatives Frakturrisiko im Falle von QFracture), können die beiden Rechner nicht austauschbar verwendet werden. Darüber hinaus kann die KMD nicht in die Schätzungen von QFracture einbezogen werden. Letztendlich beruhen die von den NICE-Qualitätsstandards empfohlenen NOGG-Interventionsschwellen auf der FRAX®-Wahrscheinlichkeit und können daher nicht mit dem von QFracture oder anderen Rechnern abgeleiteten Frakturrisiko verwendet werden.

Die Eingabe in FRAX® umfasst neben Alter und Geschlecht die in Tab. 1 (Kap. 3 „Risikofaktoren für Osteoporose“) aufgelisteten KMD-unabhängigen klinischen Risikofaktoren. Die KMD des Oberschenkelhalses ist eine optionale Eingabe. Bei den aufgeführten sekundären Ursachen wird konservativ angenommen, dass sie durch eine niedrige KMD getriggert werden und keine Relevanz haben, wenn die Schenkelhals-KMD in FRAX® eingegeben wird [4, 194]; *Evidenzgrad Ia*.

#### 6.1.2 Sonderfall Aromatasehemmer

Die endokrine Therapie des Mammakarzinoms nimmt Einfluss auf die Integrität des Knochens. Gemäß mehreren Phase-III-Studien sind Aromatasehemmer Tamoxifen überlegen. Sie werden somit primär zur adjuvanten Therapie des Hormonrezeptor-positiven Mammakarzinoms der postmenopausalen Frau sowie in Kombination mit einem Analogon des Gonadotropin-Releasing-Hormons (GnRH) zur Therapie des Hormonrezeptor-positiven Mammakarzinoms der prämenopausalen Frau mit hohem Risiko eingesetzt. Aromatasehemmer hemmen die Umwandlung von Androgenen in Östrogene und senken somit den Spiegel der Östrogene auf ein Minimum. Aromatasehemmer zur Therapie des Mammakarzinoms stellen daher Risikofaktoren für Frakturen dar, unabhängig von der Ausgangs-KMD.

Die Phase III prospektive, randomisierte placebokontrollierte Studie 18 der Austrian Breast and Colorectal Cancer Study Group (ABCSG-18) untersuchte die Effektivität von Denosumab vs. Placebo bezogen auf die Frakturrate bei postmenopausalen Frauen unter Aromatasehemmertherapie [197]; *Evidenzgrad Ib*. Gemäß dieser Studie haben Brustkrebspatientinnen nach 5 Jahren Aromatasehemmertherapie ein Frakturrisiko von 15 % und nach 7 Jahren von 20 % – unabhängig von der KMD zu Beginn der Studie oder anderen Risikofaktoren.

Denosumab 60 mg subkutan alle 6 Monate konnte die Frakturrate signifikant um 50 % senken. Als sekundärer Endpunkt wurde der Einfluss von Denosumab auf das krankheitsfreie Überleben untersucht und zusätzlich eine signifikante Verbesserung um absolute 3,5 % (Hazard Ratio [HR] 0,83; 95 % CI 0,71–0,97) berichtet.

Eine Metaanalyse randomisierter Studien, die die Effektivität von Bisphosphonaten bei Mammakarzinompatientinnen untersuchte, zeigte ebenso eine signifikante Verbesserung des krankheitsfreien Überlebens [198]; *Evidenzgrad Ia*.

### 6.2 Bestimmung des Frakturrisikos

Empfehlungen:

- Das Frakturrisiko einer Einzelperson sollte als absolutes Frakturrisiko oder, präziser ausgedrückt, als Frakturwahrscheinlichkeit über einen Zeitraum von 10 Jahren dargestellt werden; *starke Empfehlung*.
- Das weltweit wichtigste Werkzeug zur Berechnung der 10-Jahres-Frakturwahrscheinlichkeit ist FRAX®; *starke Empfehlung*.
- FRAX® ist unter Berücksichtigung des länderspezifischen Frakturrisikos sowie der länderspezifischen Mortalität auf die österreichische Bevölkerung kalibriert; es wird daher empfohlen, als Land „Österreich“ für die Berechnung des individuellen Frakturrisikos zu verwenden; *starke Empfehlung*.
- Der TBS kann ein zum FRAX® ergänzender Prädiktor des Frakturrisikos sein; *starke Empfehlung*.
- Eine Adjustierung von FRAX® soll für folgende klinische Risikofaktoren erfolgen:
  - niedrige, moderate oder hohe Glukokortikoidexposition; *bedingte Empfehlung*;
  - KMD der Lendenwirbelsäule; *starke Empfehlung*;
  - Hüftachsenlänge; *starke Empfehlung*;
  - Sturzanamnese; *bedingte Empfehlung*;
  - Herkunftsland; *starke Empfehlung*;
  - Diabetes mellitus Typ 2; *starke Empfehlung*;
  - rezente MOF; *starke Empfehlung*;
  - Anzahl vorangegangener Frakturen; *starke Empfehlung*.

Entsprechend einer konsensuellen Empfehlung der IOF und der ESCEO sollte das Frakturrisiko einer Einzelperson als „absolutes Frakturrisiko“ oder, präziser ausgedrückt, als Frakturwahrscheinlichkeit unter Einbeziehung eines Zeitraumes von 10 Jahren dargestellt werden [199]. Das Ausmaß der 10-Jahres-Frakturwahrscheinlichkeit hängt vom Alter, vom Geschlecht, der KMD sowie den vorliegenden Risikofaktoren und der Lebenserwartung ab. Der Zeitraum von 10 Jahren berücksichtigt dabei die mögliche Dauer einer erstmaligen medikamentösen Behandlung einschließlich eines anhaltenden Effekts auf das Frakturrisiko, wenn die Behandlung (vorzeitig) beendet wird [140]. Kürzere Zeiträume als 10 Jahre bringen in Bezug auf die Risikokategorisierung keinen Zusatznutzen [192, 193].

Das weltweit wichtigste Werkzeug zur Berechnung der 10-Jahres-Frakturwahrscheinlichkeit ist FRAX® (https://frax.shef.ac.uk/FRAX/tool.aspx?lang=de), ein Algorithmus, welcher im Jahr 2008 vom damaligen WHO Collaborating Centre for Metabolic Bone Diseases in Sheffield (U. K.) entwickelt wurde. Der Algorithmus selbst ist im Detail nicht publiziert, er berücksichtigt jedoch gewichtete klinische Risikofaktoren (s. Kap. 3 „Risikofaktoren für Osteoporose“) mit oder ohne Information zur KMD (s. Abschn. 5.2. „Bildgebende Verfahren“). Die 10-Jahres-Frakturwahrscheinlichkeit wird hierbei für MOF (hüftnahe Fraktur, klinisch vertebrale Fraktur, Unterarmfraktur, Humerusfraktur) insgesamt sowie für die hüftnahe Fraktur gesondert berechnet. FRAX® wurde in zahlreichen unabhängigen Kohorten extern validiert [23, 194] und unter Berücksichtigung des länderspezifischen Frakturrisikos sowie der länderspezifischen Mortalität auf rund 70 Länder weltweit, darunter auch Österreich, kalibriert [194].

Neben dem FRAX®-Tool stehen noch das QFracture-Tool (http://www.qfracture.org) sowie der Fracture Risk Calculator des Garvan Institute of Medical Research (https://fractureriskcalculator.com.au/calculator/) zur Verfügung. Ersteres berücksichtigt eine größere Anzahl klinischer Risikofaktoren ohne KMD, ist aber nur auf die britische Bevölkerung kalibriert. Letzteres kommt hingegen mit nur einigen wenigen klinischen Risikofaktoren aus, ist aber wiederum nur auf die australische Bevölkerung kalibriert.

#### 6.2.1 Möglichkeiten der Adjustierung des FRAX®-Tools

Der TBS ist eine für DXA-Osteodensitometer entwickelte Software, welche unter Verwendung von Osteodensitometrien der Lendenwirbelsäule und basierend auf Pixel-basierten Grauwertunterschieden Information zur Mikroarchitektur des Knochens bereitstellt [200] (s. Abschn. 5.2. „Bildgebende Verfahren“). Der TBS ist ein DXA-basierter, von FRAX® unabhängiger Prädiktor des Frakturrisikos [167]; *Evidenzgrad Ia*.

Für einige klinische Risikofaktoren, welche im FRAX®-Tool nicht abgebildet sind, stehen einfach anwendbare arithmetische Korrekturfaktoren zur Verfügung, z. B. für:

- eine niedrige, moderate oder hohe Glukokortikoidexposition [113]; *Evidenzgrad IIa*;
- eine KMD der Lendenwirbelsäule [165, 166]; *Evidenzgrad Ia*;
- Hüftachsenlänge [168]; *Evidenzgrad Ib*;
- Sturzanamnese [50]; *Evidenzgrad IIa*;
- Herkunftsland [201]; *Evidenzgrad Ib*;
- Typ-2-Diabetes mellitus [202]; *Evidenzgrad Ib*;
- rezente MOF [29]; *Evidenzgrad Ib*;
- Anzahl vorangegangener Frakturen [203]; *Evidenzgrad Ib*.

Für eine Kombination mehrerer dieser Korrekturfaktoren stehen keine ausreichend fundierten Daten zur Verfügung. Im Einzelfall sollte entschieden werden, welcher Risikofaktor mit größter Wahrscheinlichkeit den größten Einfluss auf die individuelle Frakturwahrscheinlichkeit hat [44].

### 6.3 Interventionsschwellen

Empfehlungen:

- Eine Ersteinschätzung der 10-Jahres-Frakturwahrscheinlichkeit für MOF (hüftnahe Fraktur, klinisch vertebrale Fraktur, Unterarmfraktur, Humerusfraktur) sowie die hüftnahe Fraktur isoliert sollte unter Verwendung der Österreich-spezifischen FRAX®-Version erfolgen, einschließlich einer Zuordnung zu einer der Österreich-spezifischen, FRAX®-basierten Risikokategorien „niedrig“, „mittel“, „hoch“ oder „sehr hoch“; *starke Empfehlung*.
- Alle Patienten mit prävalenter und/oder rezenter niedrigtraumatischer Fraktur sollten eine Osteoporose-spezifische Behandlung erhalten; *starke Empfehlung*.
- Männer und Frauen mit einem hohen oder sehr hohen Frakturrisiko sollten eine Osteoporose-spezifische Behandlung erhalten, und eine initiale DXA-Messung sollte zum Zweck des Therapiemonitorings durchgeführt werden; *starke Empfehlung*.
- Männer und Frauen, welche in der Österreich-spezifischen FRAX®-Version ein mittleres Risiko (d. h. zwischen der unteren und oberen Assessmentschwelle) aufweisen, sollen eine DXA-Messung erhalten. Nach erfolgter DXA-Messung sollte eine Neuberechnung der 10-Jahres-Frakturwahrscheinlichkeit mittels Österreich-spezifischem FRAX® erfolgen; *starke Empfehlung*.
- Wenn die KMD in das FRAX®-Tool integriert werden kann, ist für die Beurteilung der Risikokategorie (niedrig, hoch oder sehr hoch) die Region (d. h. MOF oder Hüfte) mit der höheren 10-Jahres-Frakturwahrscheinlichkeit heranzuziehen; *starke Empfehlung*.
- Männer und Frauen, welche in der Österreich-spezifischen FRAX®-Version ein mittleres Risiko (d. h. zwischen der unteren und oberen Assessmentschwelle) aufweisen, aber keine DXA-Messung erhalten können, sollten behandelt werden, wenn die Interventionsschwelle überschritten ist oder in der Anamnese eine Fragilitätsfraktur erhoben wurde; *starke Empfehlung*.
- Männer und Frauen mit einem niedrigen Risiko und ohne prävalente Fragilitätsfraktur sollen Empfehlungen zur Lebensstiloptimierung erhalten; *starke Empfehlung*.
- Männer und Frauen mit sehr hohem Frakturrisiko sollten bevorzugt einer osteoanabolen Therapie durch einen Spezialisten mit Kenntnis und Erfahrungen auf dem Gebiet der Osteoporose zugeführt werden. Folgende Indikationen kommen hierfür infrage; *bedingte Empfehlung*:
  - Vorhandensein eines einzigen, aber signifikanten Risikofaktors:
    - kürzliche (weniger als 24 Monate) MOF,
    - > 2 MOF (unabhängig vom Zeitpunkt des Auftretens),
    - KMD T‑Score ≤ −3,5;
  - Vorhandensein mehrerer klinischer Risikofaktoren, insbesondere im Falle einer kürzlich aufgetretenen Fragilitätsfraktur mit einem sehr hohen (unmittelbar bevorstehenden) Risiko einer nachfolgenden Fraktur;
  - andere Hinweise auf ein sehr hohes Frakturrisiko.
- Die Entscheidung, welche Therapie primär eingeleitet werden sollte, sollte unter Berücksichtigung des Frakturrisikos einschließlich allfälliger zusätzlicher klinischer Risikofaktoren sowie der Präferenz der Patienten getroffen werden; *starke Empfehlung*.
- Verweise auf die österreichische Version des FRAX®-Tools bzw. die Österreich-spezifischen Therapieschwellen sowie die vorliegenden österreichischen Osteoporose-Leitlinien sollten in medizinischen Befundberichten enthalten sein; *starke Empfehlung*.
- Für die Zuordnung zu einer der FRAX®-Risikokategorien ist die höhere 10-Jahres-Frakturwahrscheinlichkeit im FRAX® (MOF) heranzuziehen; *starke Empfehlung*.

**Abb. 2:**
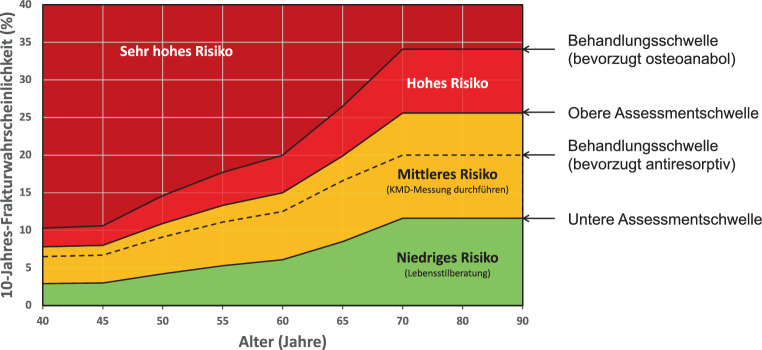
*FRAX®-basierte Assessment‑, Risiko- und Interventionsschwellen. FRAX* Fracture Risk Assessment Tool, *KMD* Knochenmineraldichte. KMD ist in dieser Abbildung nicht berücksichtigt. (Mod. und übersetzt unter einer Creative-Commons-Lizenz von [195])

Für die Ersteinschätzung der 10-Jahres-Frakturwahrscheinlichkeit vor Durchführung einer Osteodensitometrie steht eine auf die österreichische Bevölkerung kalibrierte Version des FRAX® zur Verfügung (https://frax.shef.ac.uk/FRAX/tool.aspx?lang=de) [194]; *Evidenzgrad Ib*. Die Zuordnung zu einer der Risikokategorien (niedrig, mittel, hoch, sehr hoch) gemäß Abb. 2 erfolgt dann sowohl für Männer als auch Frauen unter Verwendung der Österreich-spezifischen Schwellenwerte [195].

**Abb. 3:**
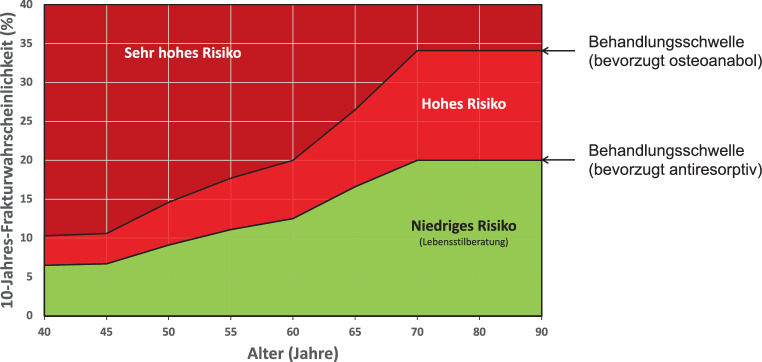
*FRAX®-basierte Interventionsschwellen für Österreich. FRAX* Fracture Risk Assessment Tool, *KMD* Knochenmineraldichte. Entspricht Abb. 2 ohne das gelbe Feld. KMD ist in dieser Abbildung nicht berücksichtigt; es wird davon ausgegangen, dass bei der Mehrzahl der Patienten vor der Einschätzung des 10-Jahres-Frakturrisikos mittels FRAX® eine KMD-Messung durchgeführt wurde. (Mod. und übersetzt unter einer Creative-Commons-Lizenz von [195])

Fällt die ermittelte 10-Jahres-Frakturwahrscheinlichkeit in die mittlere (= gelbe) Risikokategorie, sollte eine KMD-Messung durchgeführt und das Ergebnis für eine neuerliche Berechnung in das FRAX®-Tool integriert werden [22]; *Evidenzgrad Ib*. Die Durchführung einer Osteodensitometrie vor Ersteinschätzung der Frakturwahrscheinlichkeit mittels FRAX® ist mit einer vermeidbar hohen Anzahl nicht gerechtfertigter Osteodensitometrien verknüpft [195]; *Evidenzgrad Ib*. Liegt dennoch bereits eine KMD-Messung vor und wird diese für die FRAX®-basierte Risikoeinschätzung herangezogen, fällt die mittlere Risikokategorie weg (Abb. 3).

Die Interventionsschwellen für Männer und Frauen sind so festgelegt, dass sie dem Risiko einer Frau gleichen Alters mit einer prävalenten Fraktur entsprechen [7, 195, 204]. Aus diesem Grund steigen die Interventionsschwellen altersabhängig bis zum 70. Lebensjahr. Ab dem 70. Lebensjahr wird eine fixe Interventionsschwelle angewandt [205]. Die numerischen Schwellenwerte der FRAX®-basierten Österreich-spezifischen Assessment‑, Risiko- und Interventionsschwellen sind in Tab. 7 abgebildet. Eine primäre FRAX®-Einschätzung trägt zu einer deutlichen Reduktion von redundanten DXA-Messungen bei.

**Tab. 7:** Numerische Schwellenwerte für MOF und hüftnahe Frakturen, basierend auf der Österreich-spezifischen FRAX®-Version

	MOF				Hüftnahe Fraktur	
Alter, Jahre	Untere Assessmentschwelle	Obere Assessmentschwelle	Hohes Risiko (Behandlung)	Sehr hohes Risiko	Hohes Risiko (Behandlung)	Sehr hohes Risiko
40	2,9	7,8	6,5	10,3	0,6	1,0
45	3,0	8,0	6,7	10,6	0,9	1,5
50	4,2	10,9	9,1	14,6	1,4	2,2
55	5,3	13,3	11,1	17,7	1,9	3,1
60	6,1	15,5	12,5	20,0	2,7	4,4
65	8,5	19,9	16,6	26,5	4,3	6,9
70	11,6	25,6	21,3	34,1	7,1	11,3

*FRAX* Fracture Risk Assessment Tool, *MOF* „major osteoporotic fracture“ (hüftnahe Fraktur, klinisch vertebrale Fraktur, Unterarmfraktur, Humerusfraktur)

Reproduziert unter einer Creative-Commons-Lizenz von [195] und übersetzt

Anmerkung: Die genaue altersadaptierte Interventionsschwelle zwischen den Halbdekaden ist aus Abb. 2 und 3 abzulesen

Männer und Frauen mit sehr hohem Frakturrisiko sollten bevorzugt an ein auf Osteoporose spezialisiertes Zentrum zugewiesen werden, um die Indikation und Möglichkeit einer osteoanabolen Therapie abzuwägen [140, 199]. Besonderes Augenmerk wird auf den Zeitraum seit der Fraktur gelegt. Das Risiko einer Folgefraktur ist am höchsten kurz nach einer Fragilitätsfraktur (auch als imminentes Frakturrisiko bezeichnet) mit einem allmählichen Rückgang innerhalb der ersten 1 bis 2 Jahre [206]; *Evidenzgrad Ib*. Danach bleibt das Frakturrisiko zumindest über 10 Jahre im Vergleich zu einer gesunden Normalpopulation deutlich erhöht. In Anlehnung an die NOGG-Leitlinien liegt die Österreich-spezifische Schwelle zum sehr hohen Risiko 60 % über der Interventionsschwelle [195, 207].

## 7 Nicht-medikamentöse Therapie der Osteoporose

Für einen optimalen Behandlungserfolg sollte zusätzlich zur Medikation auch auf eine Reihe von Lebensstilfaktoren wie Ernährung und körperliches Training Rücksicht genommen werden. Vonseiten der Ernährung hat sich die kombinierte Gabe von Kalzium und Vitamin D – ob in Form von Präparaten oder über die reguläre Ernährung – als zentrale Maßnahme etabliert. Beim körperlichen Training gilt es, mechanische Wachstumsstimuli auf den Knochenapparat auszuüben, ohne jedoch den Knochen in seiner jeweiligen aktuellen Frakturgefährdung zu überlasten. Gegebenenfalls ist die chirurgische Versorgung einer Fraktur erforderlich.

### 7.1 Rehabilitation

Empfehlungen:

- Die Versorgung und Rehabilitation nach einer Fragilitätsfraktur soll so rasch wie möglich begonnen werden, am besten im Rahmen eines Fracture Liaison Service; *starke Empfehlung*.
- Im Fall einer Wirbelkörperfraktur soll nach entsprechender Schmerztherapie und ärztlicher Freigabe ein supervidiertes individualisiertes Trainingsprogramm begonnen werden, anfänglich, wenn nötig, in entlasteter Position; *bedingte Empfehlung*; in der Folge soll es ein progressives Kraft- und Kraftausdauertraining der Rückenmuskulatur beinhalten; *starke Empfehlung*.
- Die routinemäßige Verordnung von Orthesen im akuten oder subakuten Stadium nach Wirbelkörperfrakturen wird nicht empfohlen; *starke Empfehlung*.
- Bei peripheren Fragilitätsfrakturen sollen im Falle einer konservativen Versorgung nicht ruhiggestellte Gelenke bewegt werden; nach chirurgischer Versorgung entscheidet der Operateur bezüglich der Belastungsstabilität; *bedingte Empfehlung*.
- Zur Reduktion des Sturzrisikos werden Übungen zur Verbesserung von Gleichgewicht und Kraft (nach Möglichkeit in den Alltag eingebaut), und – wenn nötig – wird die Beseitigung von Stolperfallen empfohlen; *starke Empfehlung*.

Regelmäßige körperliche Aktivität bzw. gezieltes Training sind wesentlich in der Prävention der Osteoporose (Gonadotropin Releasing Hormone s. Abschn. 4.2. „Prävention durch Bewegung und Sport“). Ein strukturiertes Trainingsprogramm kann aber auch bei bereits bestehender Osteoporose sicher ausgeführt werden; Übungen mit ausgeprägter spinaler Flexion (manche Yogaübungen, Bauchpressen [„Sit-ups“]) sowie Reiten und Golfen sind allerdings aufgrund eines erhöhten Frakturrisikos zu meiden [208]; *Evidenzgrad IIIa*.

Die Sturzprävention ist bei Personen mit manifester Osteoporose und insbesondere bei höherem Alter besonders wichtig (s. auch Abschn. 4.2. „Prävention durch Bewegung und Sport“). Balancetraining ist am effektivsten bei Personen mit hohem Sturzrisiko [209]; *Evidenzgrad Ia*; speziell bei einer Trainingsdauer von mindestens 3 h pro Woche [135]; *Evidenzgrad Ia*. Diese Trainingsprogramme haben sich auch als sicher erwiesen [209, 210]; *Evidenzgrad Ia*. Die Kombination von Bewegung und Schulungen reduziert die Angst zu stürzen und verbessert das psychosoziale Wohlbefinden [211, 212]; *Evidenzgrad Ia*. Die Beseitigung von Stolperfallen im häuslichen Umfeld macht nur Sinn bei sturzgefährdeten Personen, und je höher das Risiko, desto größer der Benefit [213]; *Evidenzgrad Ia*.

Im initialen Management einer frischen *Wirbelkörperfraktur* erfolgt eine analgetische Therapie – wenn möglich oral [214]; *Evidenzgrad IIa*. Dem internationalen Konsensus zum Management vertebraler Frakturen entsprechend, sollte bei nachlassendem Schmerzniveau bzw. nach ärztlicher Freigabe (etwa 4 bis 12 Wochen nach der Wirbelkörperfraktur) ein individualisiertes, angeleitetes Trainingsprogramm initiiert werden [215]. Ein durch Physiotherapeuten supervidiertes Übungsprogramm verbessert Schmerz und Funktion [216]; *Evidenzgrad Ia*. Falls noch Schmerzen bestehen, ist es allerdings ratsam, die Übungen der Rückenstrecker in entlasteter Position (Rückenlage) durchzuführen [217]; *Evidenzgrad Ia*. Die Frakturheilung (etwa 12. Woche nach Fraktur) ist der Zeitpunkt, ein multimodales Trainingsprogramm (progressives Krafttraining, funktionelles Training, Balancetraining) zu beginnen [215]; *Evidenzgrad IV*. Generell gilt bei Wirbelkörperfrakturen ein Hebeverbot schwerer Lasten für 12 Wochen, bei ausgeprägter Osteoporose sogar grundsätzlich. Kyphoplastien/Vertebroplastien alleine oder in Kombination mit einer dorsalen Instrumentierung sind grundsätzlich postoperativ schmerzabhängig belastungsstabil [218]; *Evidenzgrad IIIa*. Wirbelsäulenorthesen sollen nicht generell, sondern nur nach individueller Prüfung und unter Berücksichtigung der Schmerzsituation verordnet werden, wobei akute Schmerzen den zentralen Faktor zur Verschreibung darstellen [218, 219]; *Evidenzgrad Ia*. Zur Verbesserung von Schmerz und Rumpfmuskelkraft kann das Tragen von Orthesen (2 h täglich für 6 Monate) als Maßnahme eingesetzt werden [217]; *Evidenzgrad Ia*. Elektrotherapie appliziert in der Form mittelfrequenter Ströme verbessert einer kleinen randomisierten Studie zufolge chronische Schmerzen nach einer Wirbelkörperfraktur [220]; *Evidenzgrad IIb*. Patienten mit Wirbelkörperfrakturen und/oder mehrfachen Fragilitätsfrakturen wird grundlegend empfohlen, hohe Stoßbelastungen auf die Wirbelsäule (über dem Maß alltäglicher Belastungen, z. B. höhere Stoßbelastung als bei flottem Gehen) zu vermeiden [212]; *Evidenzgrad Ia*.

*Hüftnahe Frakturen* sind postoperativ meist übungsstabil [221]; *Evidenzgrad IV*. Idealerweise folgt direkt eine Übernahme in eine Remobilisationseinrichtung mit dem Ziel des raschen Wiedererlangens des Niveaus der Alltagsaktivitäten („activities of daily living“ [ADL]) vor der Fraktur. Eine multidisziplinäre Rehabilitation insbesondere mit Einbindung progressiven Krafttrainings (stationär/ambulant) von hüftgelenksnahen Frakturen reduziert die Mortalität und verbessert die Mobilität [222, 223]; *Evidenzgrad Ia*.

Im Falle einer *distalen Radiusfraktur*, meist mit Unterarmgipsverband oder Kunststoffschiene für 4 bis 6 Wochen versorgt [224]; *Evidenzgrad Ia*; sollen Schulter (keine Schulterschlinge) und Finger nicht ruhiggestellt, sondern möglichst gut im Alltag eingesetzt werden. Operativ versorgte Frakturen sind je nach Knochenqualität (OP-Bericht) meist ab der 3. Woche übungsstabil oder ebenfalls für 4 bis 6 Wochen ruhigzustellen. Es gibt derzeit keine Evidenz, dass eine operative Versorgung von Radiusfrakturen bei Patienten > 60 Jahre der konservativen Therapie grundsätzlich überlegen ist. Daher muss die Entscheidung von Fall zu Fall getroffen werden (s. auch Abschn. 7.2. „Chirurgische Maßnahmen“) [225]; *Evidenzgrad Ia*.

Bei einer mit Schulterverband behandelten *Fraktur des proximalen Humerus* empfiehlt die European Society for Trauma and Emergency Surgery (ESTES), nach 3 Wochen mit Pendelübungen und angeleiteten Bewegungsübungen bis 90 Grad zu beginnen [226]; *Evidenzgrad IV*. Eine durch Ellbogen- und Fingerbewegungen aktivierte Muskelfunktion verbessert den Lymphabstrom und fördert den Schwellungsrückgang. Operativ versorgte Frakturen werden meist auch für 2 bis 4 Wochen in einem Schulterverband ruhiggestellt, meist mit einer passiven Beübbarkeit (Pendeln) nach 2 Wochen und aktiver Beübung nach 4 Wochen [227]; *Evidenzgrad IIIa*.

Generell gilt: Es gibt keine Kontraindikation für körperliche Aktivität/Training (s. auch Abschn. 4.2. „Prävention durch Bewegung und Sport“)!

### 7.2 Chirurgische Maßnahmen

#### 7.2.1 Hüftnahe Frakturen

Empfehlungen:

- Hüftnahe Frakturen sind zeitnah (< 48 h) operativ zu versorgen; *starke Empfehlung*.
- Bei Implantation von Hemiprothesen sollte ein zementiertes Verfahren verwendet werden; *starke Empfehlung*.
- Jede osteoporotische hüftnahe Fraktur soll noch während des stationären Aufenthaltes hinsichtlich weiterer osteoporotischer Therapie evaluiert werden; *starke Empfehlung*.
- Die Diagnose Osteoporose und die daraus resultierenden therapeutischen Konsequenzen sollen im Entlassungsbrief vermerkt werden, eine Therapieverzögerung ist zu vermeiden; *starke Empfehlung*.

Die meisten hüftnahen Frakturen sind osteoporotischer Natur. Als Traumakinematik stellt dabei der Sturz aus niedriger Höhe die Hauptursache dar. Zu den hüftnahen Frakturen zählen Frakturen des Schenkelhalses, Frakturen auf Höhe der Rollhügel (pertrochantär/intertrochantär) sowie Frakturen knapp unter der Rollhügelregion (subtrochantär). Eine konservative Behandlung ist mit einem sehr schlechten Outcome verbunden [228]; *Evidenzgrad IIIb*. Die 30-Tages-Mortalität lag in Studien bei ca. 36 %, jene nach einem Jahr bereits bei knapp 60 %. Hauptkomplikationen waren dabei Harnwegsinfektionen, Pneumonien, Sepsis, Delirium und Dekubiti. Ziel der operativen Versorgung muss daher eine schnellstmögliche Mobilisierbarkeit (s. auch Abschn. 7.1. „Rehabilitation“) und Schmerzreduktion sein.

Je nach Frakturform und Lokalisation kommen dabei kopferhaltende Verfahren (Verschraubung, dynamische Hüftschraube, Femoral-Neck-System, Marknagel mit Hüftkomponente) oder kopfersetzende Verfahren (Totalendoprothese, Hemiprothese) infrage [229]. Vor allem bei dislozierten, medialen hüftnahen Frakturen ist in der Altersgruppe ab 75 Jahren primär an eine prothetische Versorgung zu denken. Dabei sollte das zementierte Verfahren grundsätzlich bevorzugt werden [230]; *Evidenzgrad Ia*. Eine Versorgung ist am Aufnahmetag oder am Tag danach sicherzustellen [231, 232]; *Evidenzgrad IIb*; bei kopferhaltenden Verfahren und dislozierten Frakturen binnen 6 bis 12 h [221, 229]. Eine sofortige postoperative Vollbelastung ist bei den meisten Verfahren möglich, eine Teilbelastung/Entlastung ist gerade bei geriatrischen Patienten praktisch nicht möglich [233]; *Evidenzgrad IIb*.

#### 7.2.2 Periprothetische Frakturen

Empfehlungen:

- Periprothetische Frakturen sollten je nach Komplexität des Eingriffes an geeigneten Zentren zeitnah versorgt werden; *bedingte Empfehlung*.

Diese betreffen meist den Femurschaft bei vorhandenen Hüftendoprothesen und/oder Knieendoprothesen bzw. anderen osteosynthetischen Implantaten. Grundsätzlich erfolgt die Priorisierung ähnlich den hüftnahen Frakturen, ausreichende Datenlage bezüglich der operativen Versorgungsnotwendigkeit fehlt, es gelten aber ähnliche Überlegungen wie im Abschn. 7.2.1. „Chirurgische Maßnahmen – Hüftnahe Frakturen“ [234]. Aufgrund eventuell nötiger Spezialimplantate kann jedoch nicht jederzeit und an jedem Standort eine zeitgerechte Versorgung sichergestellt werden. Noch problematischer sind periprothetische Acetabulumfrakturen, die meist an Zentren versorgt werden müssen. Periprothetische Frakturen, die nicht unmittelbar die Mobilität betreffen (obere Extremitäten), sollen – wenn indiziert – elektiv an spezialisierten Einrichtungen versorgt werden [235]; *Evidenzgrad IIIb*.

#### 7.2.3 Extremitätenfrakturen

Empfehlungen:

- Distale Radiusfrakturen bei > 60-jährigen Patienten sollen primär konservativ versorgt werden; *starke Empfehlung*.

##### *Distales Femur:*

Wird meist mittels Plattenosteosynthese versorgt, Zementaugmentation der Schrauben ist bei osteoporotischem Knochen zu erwägen. Eine Revisionsprothese (distaler Femurersatz) ist bei unmöglicher Rekonstruierbarkeit zu erwägen, wobei auch die Evidenzlage bei Operation der offenen Reposition und inneren Fixierung (ORIF) vs. Megaprothese des distalen Femurs gering ist [236] und die Verplattung den Goldstandard darstellt; *Evidenzgrad IIIb*.

##### *Proximaler Humerus:*

Die meisten Frakturen v. a. bei sehr alten Patienten können konservativ behandelt werden. Als operative Verfahren kommen Verplattung (erwäge zementaugmentierte Schrauben) oder beim älteren Menschen meist die inverse Schulterprothese infrage, wobei letztere Option bei komplexeren Frakturen bessere Ergebnisse zeigt als die Osteosynthese [237]; *Evidenzgrad IIa*.

##### *Distaler Humerus:*

Ist in den meisten Fällen operativ zu versorgen (2fach Plattenosteosynthese), alternativ Ellbogenprothese bei sehr alten Patienten und intraartikulären Trümmerfrakturen [221].

##### *Distale Radiusfraktur:*

Eine konservative Therapie ist meist möglich und v. a. bei betagten Patienten auch bei größerer Fehlstellung akzeptabel und funktionell weniger bedeutend. Aktuelle Studien und Reviews zeigen keine relevanten Unterschiede zwischen konservativer und operativer Therapie bei > 60-jährigen Patienten [238]; *Evidenzgrad Ia*. Operativ kommt meist die volare Plattenosteosynthese zur Anwendung.

#### 7.2.4 Wirbelkörperfrakturen

Empfehlungen:

- Osteoporotische Wirbelkörperfrakturen (Typ A) sollen konservativ behandelt werden, wenn kein signifikanter Wirbelkörperkollaps, keine Spinalkanalkompression mit Neurologie oder Kyphose vorliegen; *starke Empfehlung*.
- Typ-B/C-Frakturen sollten operativ versorgt werden; *bedingte Empfehlung*.

Bei akuten Wirbelkörperfrakturen können sowohl Wirbelkörper (ventrale krafttragende Säule) als auch die mittleren und die hinteren Säulen (dorsale Zuggurtungsstrukturen) betroffen sein. Bei osteoporotischen Wirbelkörperfrakturen wird das Augenmerk meist auf die Wirbelkörper gelegt, Verletzungen der mittleren/hinteren Säule kommen aber auch bei Osteoporose vor und müssen ausgeschlossen werden (CT bei akuten Frakturen mandatorisch), da diese die Instabilität deutlich erhöhen können.

Laut AO-Klassifikation werden 3 Typen von Wirbelkörperfrakturen unterschieden: Typ A (Kompressionsverletzungen), Typ B (Distraktionsverletzungen) und Typ C (Translationsverletzungen). Instabile Verletzungen (Typ-B- und -C-Verletzungen nach AO mit Involvierung aller 3 Säulen der Wirbelsäule) werden wie auch bei nicht-osteoporotischen Frakturen in der Regel operativ versorgt [239]; *Evidenzgrad Ia*. Isolierte Verletzungen der Wirbelkörper werden je nachdem, ob Deckplatte und Bodenplatte bzw. die Hinterkante betroffen ist, in A1 (Kompressionsbruch nur Deckplatte), A2 (Spaltbruch Deck- und Bodenplatte), A3 (inkomplette Berstung Deckplatte und Hinterkante), A4 (komplette Berstung Deck- und Bodenplatte und Hinterkante) eingeteilt. Vor allem A3- und A4-Frakturen gelten als nachsinterungsgefährdet, und es kann dabei die dislozierte Hinterkante in unterschiedlichem Maße den Spinalkanal einengen. Die Genant-Klassifikation berücksichtigt hingegen das Ausmaß der Höhenminderung und findet v. a. bei nicht-rezenten Frakturen ihre Anwendung.

Frakturen werden bei entsprechender Höhenminderung und Kyphose in Mitteleuropa meist operativ versorgt, stabile Frakturen konservativ [44]. Eine hohe Evidenz zur operativen Versorgungsnotwendigkeit gibt es derzeit nicht. Sonderformen sind sog. Chalk-Stick-Frakturen bei verknöcherten Bewegungssegmenten (axiale Spondylarthropathie, diffuse idiopathische Skeletthyperostose). Diese gelten als hochinstabil und müssen nahezu immer operativ versorgt werden. Einen allgemeinen evidenten Beleg für den Nutzen von Wirbelsäulenmieder/-orthesen gibt es nicht [218]; *Evidenzgrad Ia*; dennoch können sie bei Bedarf zur Schmerzreduktion eingesetzt werden. Ziel aller Maßnahmen sollte die schnellstmögliche Mobilisation der Patienten sein.

## 8 Medikamentöse Therapie der Osteoporose

Die Basisprophylaxe und die medikamentöse Therapie der Osteoporose zielen auf eine individuelle Reduktion des Frakturrisikos ab. Allfällige Indikationen, Kontraindikationen und sonstige Warnhinweise der in dieser Leitlinie angeführten Medikamente sind immer den jeweils aktuellen Fachinformationen zu entnehmen. Die Fachinformationen werden nachfolgend nicht einzeln angeführt. Es wird auf die europäische Zulassungsbehörde (www.ema.org) und auf das österreichische Arzneispezialitätenregister (https://aspregister.basg.gv.at/) verwiesen.

### 8.1 Therapieziele

Allgemeine Empfehlungen:

- Die Wahl der medikamentösen Behandlung sollte von der Bewertung des Frakturrisikos, der Eignung (Alter, Kontraindikationen, Unverträglichkeiten von spezifischen Medikamenten) und Präferenz des Patienten abhängen. Bei den meisten Personen mit einem erhöhten Frakturrisiko ist eine antiresorptive Therapie die erste Wahl. Ziel jeder Osteoporosetherapie ist die Verhinderung der ersten Fraktur bzw. einer weiteren Fraktur; *starke Empfehlung*.
- Bei allen postmenopausalen Frauen und allen Männern ab dem 50. Lebensjahr soll eine FRAX®-Analyse zur Erfassung des individuellen Frakturrisikos erfolgen; *starke Empfehlung*.
- Alle Patienten ab dem 50. Lebensjahr mit einer MOF (hüftnahen Fraktur, klinisch vertebralen Fraktur, Unterarmfraktur, Humerusfraktur) sind bezüglich einer Therapieeinleitung abzuklären. Nach vertebralen oder hüftnahen Frakturen ist eine unmittelbare Therapie einzuleiten; *starke Empfehlung*.

Empfehlungen zur Basisprophylaxe:

- Kalzium- und/oder Vitamin-D-Gabe ist als Ergänzung zur medikamentösen Behandlung der Osteoporose notwendig, wenn die Kalziumzufuhr über die Nahrung niedrig ist bzw. eine Vitamin-D-Insuffizienz ein Risiko darstellt; *starke Empfehlung*.
- Der Ausgleich eines Vitamin-D-Mangels oder einer Vitamin-D-Insuffizienz vor Beginn jeder medikamentösen Osteoporosetherapie ist indiziert; *starke Empfehlung*.

Empfehlungen zu antiresorptiven Therapien:

- Bisphosphonate (Alendronat, Risedronat, Ibandronat, Zoledronat) und Denosumab sind effiziente Medikamente. Zu den alternativen Optionen gehören MHT und Raloxifen; *starke Empfehlung*.
- Nach mehrjähriger oraler antiresorptiver Vorbehandlung mit Alendronat kann auf eine antiresorptive Therapie mit Denosumab oder Zoledronat umgestellt werden; *starke Empfehlung*.
- Intravenöses Zoledronat, sofern keine Kontraindikationen vorliegen, ist als erste Behandlungsoption ab mindestens 14 Tagen nach einer Hüftfraktur möglich und empfohlen; *starke Empfehlung*.
- Denosumab, sofern keine Kontraindikationen vorliegen, ist als erste Behandlungsoption unmittelbar nach einer Hüftfraktur möglich und empfohlen; *starke Empfehlung*.
- Vor Beginn der Behandlung mit Denosumab sollte sichergestellt werden, dass ein langfristiger, individueller Osteoporosebehandlungsplan vorliegt; *starke Empfehlung*.
- Ein ungeplantes Absetzen von Denosumab ist zu vermeiden, da es zu einem erhöhten Risiko für Wirbelbrüche führen kann; daher darf es nicht abgesetzt werden, ohne eine alternative Therapie in Betracht zu ziehen; *starke Empfehlung*.
- Das Absetzen von Denosumab kann zu rapidem Knochenverlust und zu multiplen Wirbelkörperfrakturen führen. Daher sollte eine Anschlusstherapie mit einem anderen osteoprotektiven Medikament durchgeführt werden; *bedingte Empfehlung*.
- Wenn eine Denosumab-Therapie abgebrochen wird, wird eine intravenöse Infusion von Zoledronat 6 Monate nach der letzten Denosumab-Injektion empfohlen, wobei die anschließende Überwachung des Serum-CTX den Zeitpunkt der weiteren Behandlung (nochmalige Applikation nach 6 Monaten) bestimmt; *bedingte Empfehlung*.
- Bei mit Denosumab vorbehandelten Patienten sollte Denosumab parallel zur neu eingeleiteten osteoanabolen Therapie mit Teriparatid oder Abaloparatid weiter verabreicht werden, entweder über die gesamte Dauer der osteoanabolen Therapie oder einen Teil derselben; *bedingte Empfehlung*.
- Die MHT kann bei peri-/postmenopausalen Frauen mit geringem Risiko für Thrombose bzw. maligne Erkrankungen bis zu einem Lebensalter von 60 Jahren bzw. bis zu 10 Jahre nach Beginn der Menopause zur Reduktion des Frakturrisikos eingesetzt werden; *starke Empfehlung*.
- Eine Besprechung gemeinsam mit der Patientin über die weitere Anwendung einer MHT nach dem 60. Lebensjahr wird empfohlen, wobei die Entscheidung auf einer individuellen Risiko-Nutzen-Analyse beruht; *bedingte Empfehlung*.

Empfehlungen zu osteoanabolen Therapien:

- Teriparatid, Abaloparatid und Romosozumab sind Erstlinienbehandlungen für postmenopausale Frauen mit sehr hohem Frakturrisiko; *starke Empfehlung*.
- Teriparatid ist eine Erstlinientherapie bei Männern ab 50 Jahren, die ein sehr hohes Frakturrisiko haben; *starke Empfehlung*.
- Nach der zugelassenen Behandlungsdauer mit Teriparatid, Abaloparatid oder Romosozumab (24 bzw. 18 Monate oder 12 Monate) sollte unverzüglich eine antiresorptive Behandlung mit Alendronat, Zoledronat oder Denosumab eingeleitet werden; *starke Empfehlung*.
- Raloxifen soll als Option für die Folgebehandlung nach einem Osteoanabolikum bei Frauen in Erwägung gezogen werden; *bedingte Empfehlung*.
- Der Effekt einer osteoanabolen Therapie im Anschluss an eine antiresorptive Therapie hinsichtlich einer KMD-Zunahme ist in der Regel geringer als bei nicht vorbehandelten Patienten; *starke Empfehlung*.

### 8.2 Überblick über die Therapieoptionen

Die bei der Behandlung von Osteoporose eingesetzten Medikamente können je nach ihrer primären Wirkungsweise in 2 große Kategorien eingeteilt werden:

- *Antiresorptive Medikamente* hemmen in erster Linie die osteoklastische Knochenresorption mit späteren Sekundäreffekten auf die Knochenbildung.
- *Osteoanabole Medikamente* stimulieren in erster Linie die Knochenbildung über erhöhte Osteoblastenaktivität mit verschiedenen Auswirkungen auf die Knochenresorption. Diese Substanzklasse führt zu einer raschen Verbesserung der Knochenmikroarchitektur und in weiterer Folge zu einer Verbesserung der Mineralisierung.

Die meisten Medikamente können entweder in die eine oder andere Kategorie eingeordnet werden. Romosozumab jedoch hat eine duale Wirkung, indem es sowohl die Knochenbildung anregt als auch die Knochenresorption hemmt, was ebenfalls zu einer raschen Verbesserung der Mikroarchitektur und Mineralisierung führt.

Es ist wichtig, für jeden Patienten vor Beginn einer Osteoporosebehandlung eine langfristige Behandlungsstrategie zu planen, da der Zeitpunkt und die Sequenz des Einsatzes bestimmter Medikamente wichtig sind.

Die in Tab. 8 aufgeführten Arzneimittel verringern nachweislich Fragilitätsfrakturen bei postmenopausalen Frauen und, sofern angezeigt, bei Männern mit Osteoporose [44]; *Evidenzgrade Ia und Ib*.

**Tab. 8:** Wirksamkeit zugelassener Medikamente zur Behandlung von Osteoporose bei postmenopausalen Frauen und Männern in Verbindung mit Kalzium und Vitamin D gegen Frakturen

Medikament	Vertebrale Frakturen	Nicht-vertebrale Frakturen	Hüftnahe Frakturen	Nachweis der Über- oder Unterlegenheit bei der Prävention von Wirbelkörperfrakturen bei postmenopausalen Frauen mit sehr hohem Frakturrisiko	Zugelassen für Männer
Romosozumab	Ib	IIb	IIb	Überlegenheit im Vergleich zu Alendronat (Ib)	Nein
Teriparatid	Ia	Ia	Ia	Überlegenheit im Vergleich zu Risedronat (Ib)	Ja
Alendronat	Ia	Ia	Ia	Unterlegenheit im Vergleich zu Romosozumab (Ib)	Ja
Ibandronat	Ib	Ib	KE	KE	Nein
Risedronat	Ia	Ia	Ia	Unterlegenheit im Vergleich zu Teriparatid (Ib)	Ja
Zoledronat	Ia	Ia	Ia	KE	Ja
Denosumab	Ia	Ia	Ia	KE	Ja
HRT/MHT	Ia	Ia	Ia	KE	Nein
Raloxifen	Ia	KE	KE	KE	Nein

*HRT* Hormonersatztherapie, *MHT* menopausale Hormontherapie, *KE* keine Evidenz

Evidenzgrade gemäß NOGG-Leitlinien [44]

Reproduziert unter einer Creative-Commons-Lizenz aus [44] und übersetzt

#### *Vertebrale Frakturen:*

Die Wirksamkeit der in Tab. 8 aufgeführten Arzneimittel zur Prävention von Wirbelkörperfrakturen ist wissenschaftlich erwiesen. Teriparatid und Romosozumab sind Risedronat bzw. Alendronat bei der Reduktion von Wirbelkörperfrakturen bei postmenopausalen Frauen mit hohem Risiko für Osteoporose überlegen.

#### *Hüftnahe Frakturen:*

Für die meisten der in Tab. 8 aufgeführten Medikamente wurde nachgewiesen, dass sie die Häufigkeit von hüftnahen Frakturen verringern. Ausnahmen sind Ibandronat und Raloxifen.

#### *Nicht-vertebrale Frakturen:*

Die in Tab. 8 aufgeführten Medikamente (mit Ausnahme von Raloxifen) verringern nachweislich die Inzidenz von nicht-vertebralen Frakturen.

### 8.3 Therapieeinleitung

#### *Einleitung in der Primär- und Sekundärversorgung:*

Orale und intravenöse Bisphosphonate, Denosumab, Raloxifen und Hormonersatztherapie (HRT) können von Ärztinnen und Ärzten der Primär- oder Sekundärversorgung eingeleitet werden. Wird die Behandlung mit Denosumab in der Primärversorgung eingeleitet, ist es ratsam, sich mit Kolleginnen und Kollegen aus der Sekundärversorgung abzustimmen, da vor Beginn der Behandlung mit Denosumab ein langfristiger personalisierter Plan zur Osteoporosebehandlung erstellt werden soll, damit Denosumab bei Bedarf kontrolliert abgesetzt werden kann. Bei Hochbetagten unter Denosumab sollten auch die Angehörigen das Therapiekonzept kennen, damit ein unkontrolliertes Absetzen möglichst vermieden wird.

#### *Einleitung der Behandlung in der Primär- und Sekundärversorgung:*

Teriparatid, Romosozumab oder Abaloparatid sollten von Ärztinnen und Ärzten der Sekundärversorgung eingeleitet werden.

#### *Ablauf der Behandlung:*

Alle Patienten, die Denosumab absetzen bzw. Teriparatid, Abaloparatid oder Romosozumab nach 24 bzw. 18 oder 12 Monaten beenden, benötigen eine sequenzielle Therapiestrategie, die in der Regel ein langfristiges antiresorptives Medikament einschließt, das zum Zeitpunkt der Einleitung der vorigen Therapie geplant werden sollte, um eine Behandlungslücke und somit eine rasche Zunahme des individuellen Frakturrisikos zu vermeiden.

### 8.4 Kalzium und Vitamin D

Empfehlungen:

- Eine tägliche Zufuhr von zumindest 1000 mg Kalzium und 800 IE Vitamin D wird empfohlen. Eine wöchentliche Vitamin-D-Zufuhr ist akzeptabel, von Bolusgaben in größeren zeitlichen Abständen ist abzuraten; *starke Empfehlung*.
- Ein adäquater Vitamin-D-Spiegel von zumindest ≥ 20 ng/ml bzw. ≥ 50 nmol/l vor Einleitung einer spezifischen osteologischen Therapie wird empfohlen. Ein Vitamin-D-Spiegel von ≥ 50 ng/ml bzw. ≥ 125 nmol/l ist aus osteologischer Sicht nicht empfohlen; *starke Empfehlung*.
- Erkrankungen des Gastrointestinaltraktes, Malnutrition oder besondere Ernährungsformen/Unverträglichkeiten inklusive Proteinmangel sollten im Rahmen einer individuellen Ernährungsberatung thematisiert werden; *bedingte Empfehlung*.
- Der Ausgleich eines Vitamin-D-Mangels oder einer Vitamin-D-Insuffizienz vor Beginn jeder medikamentösen Osteoporosetherapie ist indiziert; *starke Empfehlung*.
- Begleitend zu allen Osteoporosetherapien muss eine Kalzium- und Vitamin-D-Supplementation durchgeführt werden, wenn die alimentäre Kalziumzufuhr nicht reicht bzw. ein Vitamin-D-Mangel vorliegt; *starke Empfehlung*.

Eine adäquate tägliche Zufuhr von Kalzium und Vitamin D ist in der Behandlung einer Osteoporose unerlässlich und kann die Frakturrate senken [44, 240, 241]; *Evidenzgrad Ia*. Eine Kalzium‑/Vitamin-D-Supplementierung kann das Risiko für Nierensteine erhöhen, nicht aber für Herz-Kreislauf- oder Krebserkrankungen [44, 242]; *Evidenzgrad Ia*.

#### 8.4.1 Kalzium

Eine tägliche Zufuhr von zumindest 1000 mg wird empfohlen [116]. Dies kann mit einer kalziumbetonten Ernährung erreicht werden (Milchprodukte und Milchalternativen, kalziumreiche Gemüse, Sesam, Mohn, Mineralwässer). Wenn dies nicht möglich ist, wird ein orales Kalziumsupplement empfohlen, um insgesamt 1000 mg täglich zu erreichen. Ein einziges zusätzliches Milchprodukt konnte in australischen Pflegeheimen das Gesamtfrakturrisiko um 33 % (HR 0,67; 95 % CI 0,48–0,93, *p* = 0,02) und das Sturzrisiko um 11 % (HR 0,89; 0,78–0,98) senken [240]; dies war auch kosteneffizient [243].

#### 8.4.2 Vitamin D

Eine tägliche Vitamin-D-Zufuhr von 20 µg (800 IE) für Erwachsene wird empfohlen [116]. Im Kontext einer Osteoporose sind häufig 800–2000 IE sinnvoll [44]; *Evidenzgrad IV*. Eine Vitamin-D-Gabe ist in aller Regel zumindest in den Wintermonaten erforderlich. Arzneimittel sind gegenüber Nahrungsergänzungsmitteln zu bevorzugen. Die sichere obere Höchstgrenze wird von den meisten Fachgesellschaften mit 100 µg (4000 IE) angegeben, vereinzelt sind aber auch deutlich höhere Dosierungen erforderlich [244]. Eine Bestimmung des 25-Hydroxy-Vitamin-D-Spiegels ist daher eine günstige Möglichkeit der Umsetzung einer personalisierten Medizin. Ein schwerer Vitamin-D-Mangel (< 12 ng/ml) kann auch über den Pathomechanismus einer Osteomalazie das Frakturrisiko erhöhen. Ein adäquater Vitamin-D-Spiegel von ≥ 20 ng/ml bzw. ≥ 50 nmol/l ist generell sinnvoll, insbesondere vor Einleitung einer spezifischen osteologischen Therapie. Serumspiegel ≥ 50 ng/ml bzw. ≥ 125 nmol/l sind aus osteologischer Sicht nicht sinnvoll und nicht erforderlich.

Es soll eine Supplementierung mit nativem Vitamin D_3_ erfolgen. Üblicherweise ist mit Vitamin D natives Vitamin D_3_ gemeint. Vereinzelt (z. B. in Japan) wird aktives Vitamin D verwendet; dies ist in Österreich nicht üblich, da das therapeutische Fenster sehr schmal ist.

Für die Empfehlung von Vitamin‑D_3_/Vitamin‑K_2_-Kombinationspräparaten besteht aktuell keine ausreichende Evidenz. Bolusdosen oder seltenere Dosierungsintervalle als wöchentlich werden nicht empfohlen, da ein erhöhtes Sturzrisiko besteht [44, 245]; *Evidenzgrad Ia.*

Eine antiresorptive Therapie bei unzureichender Kalzium‑/Vitamin D-Zufuhr kann zu schwerwiegenden Nebenwirkungen führen. Praktisch alle Zulassungsstudien haben daher eine Komedikation mit Kalzium und Vitamin D angewendet [44].

### 8.5 Protein

Eine tägliche Proteinzufuhr von zumindest 0,8 g/kgKG (Sollgewicht) und bei > 65-Jährigen von 1,0 g/kgKG wird von der DGE/ÖGE empfohlen [116].

Erkrankungen des Gastrointestinaltraktes, stattgehabte bariatrische Eingriffe, Malnutrition oder besondere Ernährungsformen/Unverträglichkeiten sollten im Rahmen einer individuellen Ernährungsberatung thematisiert werden [44].

### 8.6 Spezifische Osteoporosetherapie

#### 8.6.1 Hormonersatztherapie und menopausale Hormontherapie

Bei einer Therapie oder Prophylaxe mit (weiblichen Geschlechts‑)Hormonen muss zwischen einer „menopausalen Hormontherapie“ (MHT) und einer „Hormonersatztherapie“ (HRT) unterschieden werden. Die MHT kommt bei peri- und postmenopausalen Frauen zur Anwendung. Die HRT kann bei prämenopausalen Frauen mit keiner oder unzureichender körpereigenen Hormonproduktion eingesetzt werden.

##### *Behandlungsformen:*

Eine alleinige Behandlung mit Östrogenen (Monotherapie) ist nur bei Frauen ohne Uterus zulässig. Bei Frauen mit Gebärmutter muss als Endometriumprotektion (Senkung des Risikos für Endometriumkarzinom) eine Kombinationstherapie mit Östrogenen und Gestagenen erfolgen. Diese kann v. a. in der Peri- und frühen Menopause sequenziell erfolgen (sequenziell kombinierte MHT/HRT), d. h., dass in einem 4‑Wochen-Zyklus für 2 Wochen ein Gestagen zur Östrogengabe verabreicht wird, um eine Abbruchblutung zu erzielen. Aufgrund der deutlich niedrigeren Dosierung und der fehlenden Abbruchblutung wird in der Menopause die kontinuierliche kombinierte MHT favorisiert. Diese führt zu einer Atrophie des Endometriums nach 6 Monaten.

##### *Darreichungsformen:*

Hormone können oral, transdermal oder vaginal (meist in Form von Cremen) verabreicht werden. Aufgrund der starken hepatischen Metabolisierung benötigt man oral eine ca. 10fach höhere Dosierung als transdermal.

##### *Definition der Östrogene und Gestagene:*

Physiologische Östrogene beinhalten 17β-Östradiol (E2, potentes Östrogen), Östriol (E3) und Östron (E1). Sowohl für die MHT als auch HRT stehen konjugierte equine Östrogene (CEE), mikronisiertes 17β-Östradiol und Ethinylestradiol zur Verfügung.

Der Begriff Gestagene umfasst natürliches Progesteron sowie synthetische Gestagene, welche Progesteron‑, Testosteron- oder Spironolacton-Derivate sein können und entsprechende zusätzliche hormonelle Effekte verursachen.

##### 8.6.1.1 Peri- und Postmenopause

Empfehlungen:

- Die MHT kann bei peri-/postmenopausalen Frauen mit geringem Risiko für Thrombose bzw. maligne Erkrankungen bis zu einem Lebensalter von 60 Jahren bzw. bis zu 10 Jahre nach Beginn der Menopause zur Reduktion des Frakturrisikos eingesetzt werden; *starke Empfehlung*.
- Die Einnahmedauer der MHT sollte 10 Jahre nicht überschreiten; *starke Empfehlung*.

Östrogene beeinflussen Osteozyten (Senkung von Sclerostin) als auch Osteoklasten (Senkung von Matrixmetalloproteinase-13 [MMP-13], Steigerung von Fas-Ligand) und Osteoblasten (Senkung von „receptor activator of nuclear factor kappa B ligand“ [RANKL], Steigerung von Osteoprotegerin [OPG]) vorwiegend über den Östrogenrezeptor alpha (ERα) und sind so mitverantwortlich für die Integrität des Knochens. In der Adoleszenz überwiegen die antiresorptiven und anabolen Effekte der Östrogene auf den Knochen, sodass mit ca. 30 Jahren eine „peak bone mass“ erreicht werden kann.

Der abrupte Abfall der weiblichen Geschlechtshormone in der Menopause führt zu einem genau gegenteiligen Effekt, nämlich zu verstärkter Aktivierung von RANK, verminderter OPG-Produktion, verminderter Apoptose der Osteoklasten und infolge einer negativen Bilanz im Knochen mit vermehrtem Knochenabbau und vermindertem Aufbau oft auch resultierend in einer Reduktion der Knochenqualität. Abgesehen von sog. „Fast-Losern“, bei denen es innerhalb von einem Jahr zu massivem Verlust an KMD kommen kann, führt dieses „katabole“ Stadium des Knochens über Jahre zu einem erhöhten Frakturrisiko. Dies betrifft zunächst den spongiösen Knochen (v. a. Wirbelkörper) und später den kortikalen Knochen (Röhrenknochen, Hüfte).

Eine Metaanalyse von 107 prospektiv randomisierten Studien zeigte, dass der Einsatz einer kombinierten Östrogen-Gestagen-Therapie zu einer signifikanten Risikoreduktion von hüftnahen Frakturen um 28 % (relatives Risiko [RR] 0,72; 95 % CI 0,53–0,98), von nicht-vertebralen Frakturen um 22 % (RR 0,78; 95 % CI 0,68–0,89) und von vertebralen Frakturen um 35 % (RR 0,65; 95 % CI 0,46–0,92) im Vergleich zu Placebo führte [246]; *Evidenzgrad Ia*. Eine im Rahmen der Women’s Health Initiative (WHI) durchgeführte Studie (WHI-Studie) zeigte ebenso eine signifikante Reduktion der hüftnahen Frakturen, nicht-vertebralen und vertebralen Frakturen bei Einsatz der kombinierten Östrogen-Gestagen-Gabe bzw. der alleinigen Östrogengabe [247]; *Evidenzgrad Ib*. Diese Reduktion des Risikos betrifft sowohl Frauen mit hohem als auch niedrigem Risiko für eine Fraktur [247, 248].

Gemäß der WHI-Studie erhöht v. a. die kombinierte Hormontherapie das Risiko für Brustkrebs, Schlaganfall, Thrombose, Inkontinenz sowie Erkrankungen der Gallenblase. Vor allem der Einfluss auf koronare Herzerkrankung und Karzinome (u. a. Mammakarzinom, Kolonkarzinom) ist stark abhängig von Beginn und Dauer der Therapie. Ein rezenter Review zeigt ein akzeptables Nebenwirkungsprofil für Frauen jünger als 60 Jahre (50 bis 59 Jahre) bzw. mit Dauer der Therapie nicht länger als 10 Jahre nach Beginn der Menopause [249]; *Evidenzgrad IIa*. Die Indikation für eine MHT (aber auch HRT) sollte bei Patientinnen mit kardiovaskulären bzw. onkologischen Risikofaktoren mit großer Zurückhaltung erfolgen. Im Hinblick auf das Risikoprofil sollte bevorzugt natürliches Progesteron oder Dydrogesteron als Gestagen angewandt werden. Mit zunehmender Einnahmedauer steigt das Risiko unter anderem für das Mammakarzinom je nach Wahl des Gestagens an, sodass die Einnahmedauer 10 Jahre nicht überschreiten sollte [250, 251]; *Evidenzgrad IIb*. Regelmäßige fachärztliche Kontrollen sind unter Therapie indiziert.

##### 8.6.1.2 Prämenopause

Empfehlungen zur Sonderform „premature ovarian insufficiency“:

- Die HRT soll als erste Wahl eingesetzt werden – bei fehlenden Kontraindikationen –, um die Knochengesundheit bei Patientinnen mit POI aufrechtzuerhalten; *starke Empfehlung*.
- Die HRT sollte bis zum Alter der natürlichen Menopause von durchschnittlich 51 Jahren bei Patientinnen mit POI erfolgen; *starke Empfehlung*.

„Premature ovarian insufficiency“ (POI) ist definiert als ein Verlust der ovariellen Funktion vor dem 40. Lebensjahr. Die Ätiologie der POI ist mannigfaltig und beinhaltet unter anderem genetische Veränderungen wie beispielsweise das Turner-Syndrom, Autoimmunerkrankungen wie die Thyreoiditis Hashimoto, Diabetes mellitus Typ 1, Infektionserkrankungen, aber auch iatrogene Ursachen wie z. B. die risikoreduzierende bilaterale Salpingoophorektomie, Operationen an den Ovarien aus benignen (Endometriose) und malignen Indikationen, Radiatio oder der Einsatz von Chemotherapie.

Das Beurteilen der Knochengesundheit mittels DXA ist bei Patientinnen mit POI schwierig, da der T‑Score erst nach Erreichen der „peak bone mass“ eingesetzt werden kann und z. B. der Habitus bei Turner-Patientinnen zu falsch positiven Ergebnissen der KMD führen kann [252]. Ein Z‑Score von ≤ −2 wird als geringe KMD angesehen. Das Frakturrisiko von Frauen mit POI kann nicht durch FRAX® erfasst werden [148]. Es ist zudem so, dass aufgrund des Alters das durchschnittliche Frakturrisiko der POI-Patientin gering ist. Stärkster Risikofaktor für eine Fraktur und spätester Zeitpunkt, eine spezifische Therapie einzuleiten, ist eine Fragilitätsfraktur selbst.

Die HRT – wenn nicht kontraindiziert (Thrombose, maligne Erkrankung) – ist die primäre Form der Prävention/Therapie der Patientinnen mit POI [253]; *Evidenzgrad IIa*. Bezogen auf die Knochengesundheit, scheinen 100–150 μg transdermales oder 2 mg orales Östradiol einer Gabe von 30 μg Ethinylestradiol überlegen zu sein. Eine HRT sollte bis zum Eintritt der normalen Menopause erfolgen, welche in Europa im Schnitt im Alter von 51 Jahren auftritt.

#### 8.6.2 Selektive Östrogenrezeptormodulatoren

Empfehlungen:

- Raloxifen kann bei postmenopausalen Frauen – v. a. bei erhöhtem Brustkrebsrisiko – zur Reduktion von vertebralem Frakturrisiko therapeutisch und präventiv eingesetzt werden; *starke Empfehlung*.
- Raloxifen sollte nicht bei prämenopausalen Frauen zur Therapie oder Prävention der Osteoporose eingesetzt werden; *bedingte Empfehlung*.

Selektive Östrogenrezeptormodulatoren (SERM) umfassen eine Substanzklasse, welche in Abhängigkeit von gewebeabhängigen Co-Repressoren bzw. Co-Aktivatoren antagonistische oder agonistische Effekte am Östrogenrezeptor erzielen.

Alle klinisch untersuchten SERM, wie z. B. Tamoxifen, Raloxifen, Bazedoxifen oder Lasofoxifen, weisen antagonistische Effekte im Mammaparenchym auf, was sowohl therapeutisch (Tamoxifen) als auch präventiv (Tamoxifen, Raloxifen) bezogen auf das Mammakarzinom genutzt wird. Im Endometrium wirken Raloxifen und Bazedoxifen antagonistisch, während Tamoxifen eher agonistische Effekte erzielt, was sich in den Nebenwirkungen der Endometriumhyperplasie bis hin zum Endometriumkarzinom bei Tamoxifen – nicht aber bei Raloxifen oder Bazedoxifen – widerspiegelt.

Alle klinisch untersuchten SERM weisen eine agonistische Wirkung im Knochen auf. So reduziert der Einsatz von Raloxifen das Auftreten von vertebralen, nicht aber nicht-vertebralen Frakturen oder Hüftfrakturen um 41 % (RR 0,59; 95 % CI 0,46–0,76) [246]; *Evidenzgrad Ia.* Raloxifen 60 mg per os/Tag reduziert das Risiko vertebraler Frakturen bei erniedrigter KMD (T-Score −2,5 oder weniger) mit oder ohne vorangegangener Fraktur [254]; *Evidenzgrad Ib*. Raloxifen reduziert das Frakturrisiko auch unabhängig von der Präsenz von Frakturrisikofaktoren und kann somit präventiv eingesetzt werden [255]; *Evidenzgrad Ib.* Raloxifen senkt bei Frauen mit entsprechender Prädisposition das Risiko, an invasivem Brustkrebs zu erkranken.

Unerwünschte Nebenwirkungen umfassen ein erhöhtes Thromboserisiko, Wechselbeschwerden, Beinkrämpfe und minimal erhöhtes Risiko für Schlaganfall. Da SERM am Knochen einen geringer potenten agonistischen Effekt am Östrogenrezeptor aufweisen als Östradiol, zeigt die Literatur einen Abfall der KMD bei Anwendung von Tamoxifen oder Raloxifen bei prämenopausalen Frauen.

Raloxifen ist der einzige SERM, welcher derzeit in Österreich zur Behandlung und Prävention der Osteoporose bei postmenopausalen Frauen eingesetzt wird. Bazedoxifen weist ähnliche Effekte bezogen auf den Knochen (vertebrale Frakturen: RR 0,61; 95 % CI 0,41–0,90) [246] und Nebenwirkungen wie Raloxifen auf, steht aber in Österreich für die Osteoporosetherapie nicht zu Verfügung. Für Lasofoxifen wiederum ist die Gültigkeit der Marktzulassung für die Osteoporosetherapie in der Europäischen Union im Jahr 2012 erloschen.

#### 8.6.3 Antiresorptive Medikamente

Empfehlungen:

- Patienten mit hohem Frakturrisiko ohne prävalente Frakturen sollen primär mit antiresorptiven Substanzen behandelt werden; *starke Empfehlung*.
- Bisphosphonate (Alendronat, Risedronat, Ibandronat, Zoledronat) und Denosumab sind effiziente Medikamente. Zu den alternativen Optionen gehören MHT und Raloxifen; *starke Empfehlung*.
- Nach mehrjähriger oraler antiresorptiver Vorbehandlung mit Alendronat kann auf eine antiresorptive Therapie mit Denosumab oder Zoledronat umgestellt werden; *starke Empfehlung*.
- Intravenöses Zoledronat, sofern keine Kontraindikationen vorliegen, ist als erste Behandlungsoption ab mindestens 14 Tagen nach einer hüftnahen Fraktur möglich; *starke Empfehlung*.

##### 8.6.3.1 Bisphosphonate

**Abb. 4:**
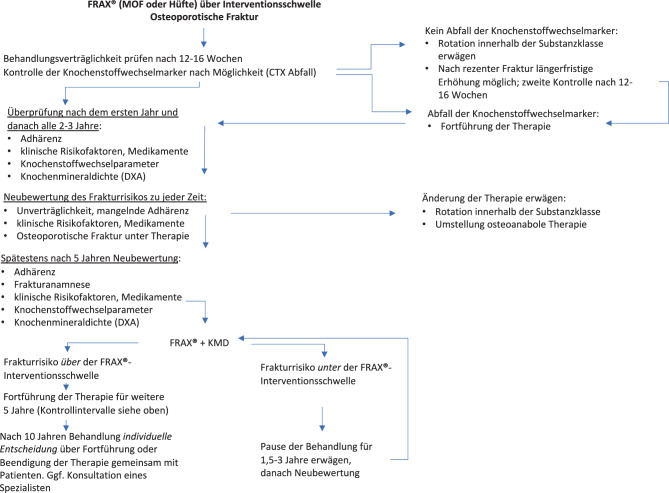
*Orale Bisphosphonate: Klinisches Flussdiagramm für Langzeitbehandlung und Therapiemonitoring. CTX* „C-terminal crosslinking telopeptides of type I collagen“, *DXA* 2-Spektren-Röntgenabsorptiometrie, *FRAX* Fracture Risk Assessment Tool, *KMD* Knochenmineraldichte, *MOF* „major osteoporotic fracture“ (hüftnahe Fraktur, klinisch vertebrale Fraktur, Unterarmfraktur, Humerusfraktur)

Bisphosphonate hemmen die Knochenresorption, sie sind in oraler und intravenöser Form verfügbar. Ein Nichtansteigen der KMD unter Bisphosphonat-Therapie ist kein Hinweis auf fehlende Wirksamkeit in Bezug auf das Knochenbruchrisiko. Ein klinisches Flussdiagramm für die Langzeitbehandlung und das Therapiemonitoring findet sich in den Abb. 4 (orale Bisphosphonate) und 5 (intravenöse Bisphosphonate).

*Alendronat 70* *mg 1‑mal wöchentlich oral *wird zur Therapie der postmenopausalen Osteoporose, zur Therapie der Osteoporose bei Männern sowie zur Therapie und Prävention der glukokortikoidinduzierten Osteoporose (GIOP) empfohlen. Bei postmenopausalen Frauen kommt es zu einer signifikanten Reduktion des Wirbelkörperfrakturrisikos, des Risikos von hüftnahen Frakturen und von nicht-vertebralen Frakturen [256]; *Evidenzgrad Ib*. Die Zulassung von Alendronat in den Indikationen männliche Osteoporose und GIOP beruht auf sog. „Bridging-Studien“ mit Analysen der Zunahme der KMD [257, 258]; *Evidenzgrad Ib*. Obwohl in der Indikation „männliche Osteoporose“ nur die tägliche Dosis mit 10 mg oral zugelassen ist, wird die 1‑mal wöchentliche 70-mg-Gabe als gleich effektiv eingestuft; *Evidenzgrad IV*.

Die Einnahme von Alendronat muss in der Früh nüchtern mit mindestens 120 ml Leitungswasser erfolgen. Der Patient muss in einer aufrechten Körperposition verharren und darf frühestens nach 30 min andere Medikamente oder Nahrung zu sich nehmen. Die häufigsten unerwünschten Nebenwirkungen umfassen gastrointestinale Beschwerden, Kopf- und Muskelschmerzen.

*Risedronat 35* *mg 1‑mal wöchentlich oral* wird zur Therapie der postmenopausalen Osteoporose, zur Therapie der Osteoporose bei Männern, zur Therapie und Prävention der GIOP bei Frauen sowie zur Therapie der GIOP bei Männern empfohlen. Für Risedronat wurde die Effektivität der 1‑mal wöchentlichen Gabe von 35 mg durch Äquivalenzstudien mit KMD-Messungen von den Zulassungsstudien mit der täglichen 5‑mg-Gabe hergeleitet [259, 260]; *Evidenzgrad Ib*. In einer Population älterer Patientinnen wurde eine signifikante Reduktion des Risikos von hüftnahen Frakturen beschrieben [41]; *Evidenzgrad Ib*. Auch die Zulassung von Risedronat in den Indikationen GIOP und Osteoporose bei Männern beruht auf Bridging-Studien [261–263]; *Evidenzgrad Ib*.

Die Einnahme von Risedronat muss in der Früh nüchtern mit mindestens 120 ml Leitungswasser erfolgen. Der Patient muss in einer aufrechten Körperposition verharren und frühestens nach 30 min andere Medikamente oder Nahrung zu sich nehmen. Die häufigsten unerwünschten Nebenwirkungen umfassen gastrointestinale Beschwerden, Kopf- und Muskelschmerzen.

*Ibandronat 150* *mg 1‑mal pro Monat oral oder 3* *mg intravenös alle 3 Monate *wird zur Therapie der postmenopausalen Osteoporose empfohlen. Sowohl die 1‑mal monatliche orale als auch die quartalsweise intravenöse Therapie wurden zugelassen auf der Grundlage von Bridging-Studien zur 2,5 mg täglichen oralen Therapie, welche eine signifikante Reduktion von Wirbelkörperfrakturen zeigen konnten [264–266]; *Evidenzgrad Ib*. Daten zur Reduktion des Risikos von hüftnahen Frakturen liegen nicht vor.

Die Einnahme muss morgens nüchtern mindestens 1 h vor der Einnahme anderer Speisen oder Getränke mit 180–240 ml Leitungswasser erfolgen. Für die nächsten 60 min muss eine aufrechte Körperposition eingenommen werden. Bei oraler Therapie sind gastrointestinale Probleme typische Nebenwirkungen. Bei intravenöser Injektion kann es bei einem geringen Prozentsatz von Patienten nach der ersten Injektion zu einer sog. Akutphasenreaktion kommen, welche mit grippeähnlichen Symptomen einhergeht und selbstlimitierend ist.

*Zoledronat 5* *mg als Kurzinfusion mit 5* *mg/100* *ml 1‑mal jährlich* wird zur Therapie der postmenopausalen Osteoporose, zur Therapie der Osteoporose bei Männern und zur Therapie der GIOP empfohlen. Bei Frauen mit postmenopausaler Osteoporose konnte neben dem Risiko für Wirbelkörperfrakturen auch jenes für nicht-vertebrale und hüftnahe Frakturen gesenkt werden [267]; *Evidenzgrad Ib*. Die Zulassung für Männer mit Osteoporose und GIOP erfolgte wiederum über Bridging-Studien [268, 269]; *Evidenzgrad Ib*. Bei Patienten nach hüftnahen Frakturen konnte durch eine Gabe von Zoledronat 5 mg intravenös, beginnend zeitnah nach der Fraktur, nach 3 Jahren nicht nur eine Risikoreduktion neuer klinischer Frakturen, sondern auch eine geringere Mortalität gezeigt werden [270]; *Evidenzgrad Ib*. Postmenopausale ältere Frauen mit Osteopenie erhielten über 6 Jahre Zoledronat 5 mg intravenös im Abstand von 18 Monaten. Auch dieses Kollektiv zeigte eine Reduktion der vertebralen und nicht-vertebralen Frakturen [271, 272]; *Evidenzgrad Ib*. Ein geringer, wenn auch nicht signifikanter Rückgang der Mortalität sowie eine reduzierte Brustkrebsinzidenz und eine reduzierte allgemeine Karzinominzidenz unter Zoledronat wurden beschrieben [271, 272].

Zoledronat 5 mg als Kurzinfusion mit 5 mg/100 ml 1‑mal jährlich sollte langsam (Minimum über 15 min) intravenös verabreicht werden. Eine typische Nebenwirkung nach der ersten Infusion ist auch hier die Akutphasenreaktion, die nach der ersten Infusion in rund einem Drittel der Patienten auftreten und durch eine Paracetamol-Gabe abgeschwächt werden kann. Nur in der Zulassungsstudie wurde als Nebenwirkung symptomatisches Vorhofflimmern dokumentiert [267]; *Evidenzgrad Ib*.

**Abb. 5:**
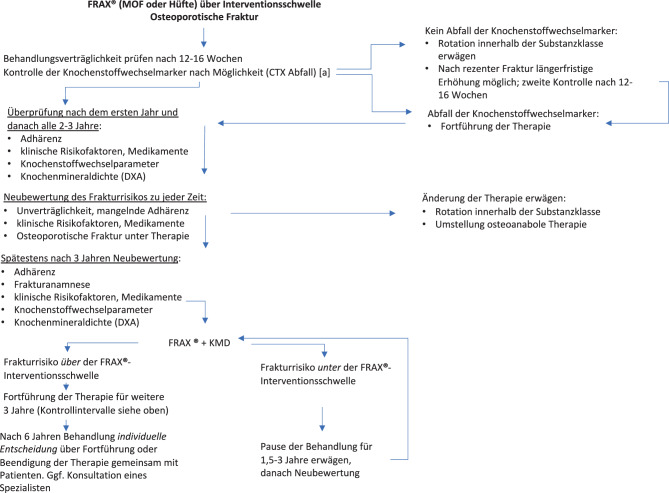
*Intravenöse Bisphosphonate: Klinisches Flussdiagramm für Langzeitbehandlung und Therapiemonitoring. CTX* „C-terminal crosslinking telopeptides of type I collagen“, *DXA* 2-Spektren-Röntgenabsorptiometrie, *FRAX* Fracture Risk Assessment Tool, *i.v.* intravenös, *KMD* Knochenmineraldichte, *MOF* „major osteoporotic fracture“ (hüftnahe Fraktur, klinisch vertebrale Fraktur, Unterarmfraktur, Humerusfraktur). [a] ausgenommen Zoledronsäure i.v. 5 mg 1‑mal/Jahr

##### *Kontraindikation für Bisphosphonate:*

Bisphosphonate sind kontraindiziert bei schwangeren oder stillenden Frauen, bei Hypokalziämie und bei Allergien gegen die Substanzgruppe. Orale Präparate sind kontraindiziert bei Personen mit Schluckbeschwerden (Achalasie, Strikturen der Speiseröhre) und bei all jenen, die nicht zumindest 30–60 min aufrecht stehen oder sitzen können. Besonderes Augenmerk ist bei Patienten mit eingeschränkter Nierenfunktion geboten. Ab einer GFR < 35 ml/min sind Zoledronat und Alendronat kontraindiziert. Auch für Ibandronat und Risedronat liegt die Schwelle bei einer GFR < 30 ml/min. Mit Vorsicht sind orale Präparate bei Patienten mit gastrointestinalen Erkrankungen zu verwenden. Eine vorbestehende Hypokalziämie muss abgeklärt werden, ein Vitamin-D-Mangel muss vor Therapiebeginn ausgeglichen werden.

##### 8.6.3.2 Denosumab

Empfehlungen:

- Vor Einleitung einer Denosumab-Therapie soll eine fortwährende Betreuung des Patienten sichergestellt werden. Eine Pause oder ein Absetzen der Medikation sollte nur nach Rücksprache mit einem Spezialisten erfolgen; *starke Empfehlung*.
- Denosumab wird nicht renal ausgeschieden und kann unbedenklich bei Patienten bis CKD 3b gegeben werden; *starke Empfehlung*.
- Das Absetzen von Denosumab kann zu rapidem Knochenverlust und zu multiplen Wirbelkörperfrakturen führen. Daher sollte eine Anschlusstherapie mit einem anderen osteoprotektiven Medikament durchgeführt werden; *bedingte Empfehlung*.
- Sechs Monate nach der letzten Denosumab-Injektion, sofern Denosumab abgesetzt wird, sollte eine Zoledronat-Infusion erfolgen mit anschließenden regelmäßigen Kontrollen des Resorptionsmarkers CTX; *bedingte Empfehlung*.
- Ist ein Monitoring des Knochenstoffwechsels nicht möglich, sollte nach 6 Monaten die nächste Zoledronat-Infusion verabreicht werden; *bedingte Empfehlung*.
- Wenn eine Zoledronat-Infusion nicht möglich ist, sollte eine orale Alendronat-Therapie erfolgen; *bedingte Empfehlung*.
- Ein regelmäßiges Monitoring des Knochenstoffwechsels unter Therapie wird empfohlen; *bedingte Empfehlung*.

**Abb. 6:**
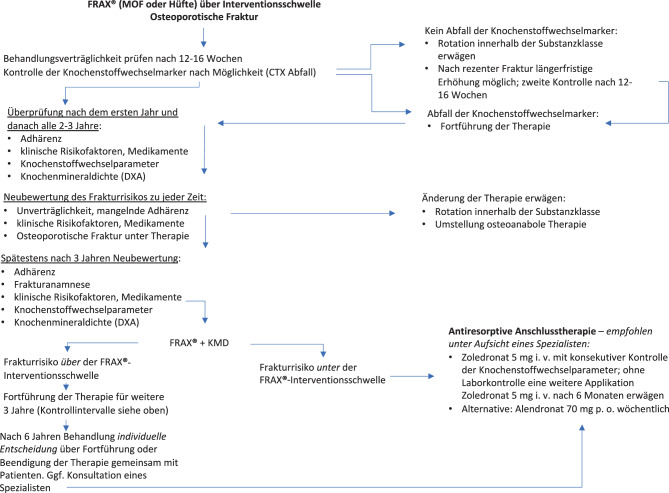
*Denosumab: Klinisches Flussdiagramm für Langzeitbehandlung und Therapiemonitoring. CTX* „C-terminal crosslinking telopeptides of type I collagen“, *DXA* 2-Spektren-Röntgenabsorptiometrie, *FRAX* Fracture Risk Assessment Tool, *KMD* Knochenmineraldichte, *MOF* „major osteoporotic fracture“ (hüftnahe Fraktur, klinisch vertebrale Fraktur, Unterarmfraktur, Humerusfraktur)

Ein klinisches Flussdiagramm für die Langzeitbehandlung mit Denosumab und das Therapiemonitoring findet sich in Abb. 6. Denosumab ist ein humaner monoklonaler IgG2-Antikörper, der an RANKL bindet und alle 6 Monate subkutan verabreicht wird. Durch die Blockade von RANKL kommt es zu einer ausgeprägten Hemmung der Osteoklastenaktivität, zum Rückgang der Osteoklastenbildung und zur verkürzten Lebensdauer der Osteoklasten.

Halbjährlich werden 60 mg Denosumab subkutan verabreicht. Denosumab zeigt eine dosisabhängige Wirkung – die 1‑malige Gabe von subkutanem Denosumab 0,01–3,0 mg/kg bewirkt eine rasche (innerhalb von 12 h), deutliche (84 %) und anhaltende (6 Monate) Reduktion der Osteoklastenaktivität [273].

Die Zulassungsstudie „Fracture Reduction Evaluation of Denosumab in Osteoporosis Every 6 Months“ (FREEDOM) mit postmenopausalen Frauen hat eine 68 % relative Risikoreduktion (RRR) neuer Wirbelkörperfrakturen, 40 % RRR neuer hüftnaher Frakturen und 20 % RRR nicht-vertebraler Frakturen während der 3‑jährigen verblindeten Phase gezeigt. Die Verlängerungsstudie zeigte außerdem 10-Jahres-Langzeitdaten, welche die anhaltende Wirkung und Therapiesicherheit von Denosumab belegen [274, 275]; *Evidenzgrad Ib*.

Im Gegensatz zu den Bisphosphonaten wird Denosumab nicht renal ausgeschieden und kann daher unbedenklich auch Patienten mit eingeschränkter Nierenfunktion gegeben werden [276, 277]; *Evidenzgrad Ib*. In die FREEDOM-Studie wurden auch Patientinnen mit CKD 3 eingeschlossen, und während der 10-jährigen Beobachtungszeit blieb in 64 % der Population die Nierenfunktion stabil, lediglich 3 % zeigten eine Progression und wurden als CKD 4 reklassifiziert [274, 276]. Die Effektivität und Sicherheit von Denosumab ist unabhängig von der Nierenfunktion, und ein Monitoring derselben ist nicht notwendig.

Denosumab ist zugelassen zur Therapie der postmenopausalen Osteoporose mit erhöhtem Frakturrisiko und zur Therapie der Osteoporose bei Männern mit erhöhtem Frakturrisiko. Des Weiteren ist Denosumab zugelassen zur Behandlung von Knochenschwund im Zusammenhang mit Hormonablation bei Männern mit Prostatakarzinom mit erhöhtem Frakturrisiko und zur Behandlung von Patienten mit GIOP und erhöhtem Frakturrisiko.

Die Zulassung in der Indikation Osteoporose bei Männern beruht auf Bridging-Studien mit Analysen der Zunahme der KMD [278, 279]; *Evidenzgrad Ib*. Denosumab zeigte eine signifikante Frakturreduktion für vertebrale, nicht-vertebrale und hüftnahe Frakturen unabhängig vom Lebensalter und Frakturrisiko. Eine Post-hoc-Analyse der FREEDOM-Studie analysiert die Frakturreduktion bei Frauen mit sehr hohem Risiko (Alter > 75 Jahre, prävalente Wirbelkörperfraktur, T‑Score an der Hüfte < −2,5) [280]; *Evidenzgrad IIa*.

Zu den Kontraindikationen von Denosumab zählen Hypokalziämie und Unverträglichkeit gegen die Substanz. Vor Therapiebeginn muss eine vorbestehende Hypokalziämie abgeklärt und ein Vitamin-D-Mangel ausgeglichen werden. Eine ausreichende Kalzium- und Vitamin-D-Einnahme wird empfohlen und ist insbesondere bei Patienten mit eingeschränkter Nierenfunktion und anderen Erkrankungen, welche mit einem Hypokalziämierisiko einhergehen, von Bedeutung. Eine Serumkalziumkontrolle vor jeder Denosumab-Injektion wird in diesem Kollektiv empfohlen, bei Patienten mit einer GFR < 35 ml/min sollte 2 Wochen nach der Injektion eine zusätzliche Kalziumbestimmung erfolgen.

Es gibt keine Empfehlungen für den Einsatz der Therapie bei Schwangeren oder Patienten vor dem vollendeten 18. Lebensjahr [274, 281]. Nebenwirkung sind lokale Hautreaktionen wie Infektionen, Zellulitis und Ekzeme, aber auch Hypokalziämie und Flatulenz [274, 275]. Nach einer Denosumab-Injektion kann es kurzfristig und passager zu einem leichten Anstieg des PTH-Spiegels kommen. Sehr seltene Nebenwirkungen wie Kieferosteonekrose („osteonecrosis of the jaw“ [ONJ]) und atypische Femurfrakturen (AFF) werden gesondert (s. Abschn. 8.9. „Nebenwirkungen der Osteoporosetherapie“) besprochen.

Nach dem Absetzen der Denosumab-Therapie kommt es zu einem beschleunigten Knochenstoffwechsel und Verlust der KMD [282–284]; *Evidenzgrad Ib.* Eine Post-hoc-Analyse der FREEDOM-Studie zeigt, dass nach Absetzen der Denosumab-Therapie ein erhöhtes Risiko für multiple Wirbelkörperfrakturen besteht. Nach Ausscheiden aus der Studie traten bei 60,7 % der Patientinnen der Therapiegruppe multiple Wirbelbrüche auf, während in der Placebogruppe signifikant (*p* = 0,049) weniger Frauen betroffen waren (38,7 %) [285, 286]; *Evidenzgrad Ib*.

Das Absetzen von Denosumab kann zu rapidem Knochenverlust und zu multiplen Wirbelkörperfrakturen führen. Daher erscheint eine Anschlusstherapie mit einem anderen osteoprotektiven Medikament sinnvoll [284] und wird empfohlen. Eine intravenöse Therapie mit Zoledronat 5 mg kann den Knochenverlust reduzieren, obwohl der protektive Effekt nicht ein volles Jahr anhält und nicht für alle Patienten nachweisbar ist [287–292]. Vor allem Patienten, die über einen Zeitraum von mehr als 3 Jahren mit Denosumab behandelt wurden, weisen dennoch einen deutlichen Knochenverlust auf [293, 294]; *Evidenzgrad IIa* und *IIb*. Hier hilft die Bestimmung der Knochenstoffwechselparameter bei der Entscheidungsfindung, ob und wann eine zweite Zoledronat-Infusion notwendig ist. Wenn eine Laboranalyse nicht möglich ist, sollte die Zweitinfusion nach 6 Monaten erfolgen [295]; *Evidenzgrad IV*.

Ein alternativer Versuch, den Knochenverlust nach Denosumab zu verhindern oder zumindest zu mitigieren, ist eine orale Anschlusstherapie mit Alendronat 70 mg 1‑mal wöchentlich oral. Unter dieser Therapie konnte beim Großteil der Patienten die KMD stabilisiert werden; 15,9 %, 7,6 % und 21,7 % zeigten jedoch einen KMD-Verlust an der Lendenwirbelsäule, der Hüfte gesamt und in der Schenkelhalsregion [296]; *Evidenzgrad IIa*.

In Anbetracht der vorliegenden publizierten Daten ist das Absetzen einer Denosumab-Therapie ein komplexer Prozess. Aus diesem Grund sollte eine Denosumab-Therapie bei jungen postmenopausalen Frauen respektive gleichaltrigen Männern kritisch hinterfragt werden und unter Observanz eines Spezialisten erfolgen.

#### 8.6.4 Osteoanabole Therapie

Empfehlungen:

- Bei Frauen mit postmenopausaler Osteoporose und sehr hohem Frakturrisiko, insbesondere bei vorbestehenden Wirbelkörperfrakturen, sollten die osteoanabolen Therapien Teriparatid, Abaloparatid oder Romosozumab als Erstlinientherapie eingesetzt werden; *starke Empfehlung*.
- Bei Männern ab dem 50. Lebensjahr mit sehr hohem Frakturrisiko, insbesondere bei vorbestehenden Wirbelkörperfrakturen, sollte Teriparatid als Erstlinientherapie eingesetzt werden; *starke Empfehlung*.
- Nach Beendigung der Therapie mit Teriparatid, Abaloparatid oder Romosozumab (24, 18 bzw. 12 Monate) sollte unmittelbar eine antiresorptive Anschlusstherapie mit Alendronat, Zoledronat oder Denosumab eingeleitet werden; *starke Empfehlung*.
- Der Effekt einer osteoanabolen Therapie im Anschluss an eine antiresorptive Therapie hinsichtlich einer KMD-Zunahme ist in der Regel geringer als bei nicht vorbehandelten Patienten; *starke Empfehlung*.
- Bei postmenopausalen Frauen ist eine anschließende Raloxifen-Therapie eine mögliche Alternative; *bedingte Empfehlung*.

##### 8.6.4.1 Teriparatid (rekombinantes humanes Parathormon rhPTH 1-34)

Teriparatid wurde 2002 in den USA und 2003 in Europa zur Therapie der postmenopausalen Osteoporose bei Frauen mit hohem Frakturrisiko zugelassen. Mittlerweile umfasst die Zulassung auch die männliche Osteoporose sowie GIOP [277]. Der osteoanabole Effekt erfolgt über eine Stimulation der Osteoblasten und der daraus resultierenden vermehrten Synthese von Knochenmikroarchitektur, Zunahme der Knochenstärke und Frakturreduktion [297].

Einmal täglich wird eine Dosis von 20 µg subkutan appliziert, und die Therapiedauer ist auf 24 Monate beschränkt. Diese Zeitspanne wurde aufgrund eines erhöhten Osteosarkomrisikos bei Ratten, welche mit Teriparatid behandelt worden waren, festgelegt [298]. Bei Menschen unter Teriparatid-Therapie ist in Post-Marketing-Studien kein erhöhtes Osteosarkomrisiko beschrieben, worauf die FDA im November 2020 die zeitliche Limitierung auf die Therapiedauer von 2 Jahren aufgehoben hat [298–300]. Eine neuerliche Therapie bei Patienten mit sehr hohem bzw. erneut sehr hohem Frakturrisiko ist damit möglich. Weiters wird ein Beibehalten der Therapie bei Patienten, welche auch nach 2 Jahren erhöhte P1NP-Serumspiegel als Hinweis auf einen persistierenden anabolen Knochenstoffwechsel aufweisen, empfohlen [299]. Zeitgleich hat die FDA auch den Hinweis auf ein potenzielles Osteosarkomrisiko aus der Fachinformation gelöscht [300]. Seitens der EMA ist bislang keine Änderung der Zulassung erfolgt, daher wird ein zweiter Zyklus derzeit nicht empfohlen. Sofern die Indikation als Einzelfallentscheidung gestellt wird, sollte dieser unter Aufsicht eines Experten verabreicht werden.

Die Zulassungsstudie zeigt in einer Population postmenopausaler Frauen mit manifester Osteoporose (≥ 2 milde oder ≥ 1 moderate Wirbelkörperfraktur) eine signifikante RRR für eine neue Wirbelkörperfraktur um 65 %, für neue moderate oder schwere Frakturen um 90 %, für multiple vertebrale Frakturen um 77 % und für nicht-vertebrale Frakturen um 35 % [301]; *Evidenzgrad Ib*. Für die Reduktion des Risikos von hüftnahen Frakturen liegen keine primären Endpunktstudien vor, jedoch zeigen Metaanalysen und ein systematischer Literaturreview eine RRR von 56 %, während kein Effekt auf Radius- und Humerusfrakturen vorliegt [302]; *Evidenzgrad Ia*. Eine Metaanalyse randomisierter Studien mit Teriparatid ergab eine RRR des Risikos von hüftnahen Frakturen von 65 % [303]; *Evidenzgrad Ia*.

Die Zulassung von Teriparatid in der Indikation Osteoporose der Männer und GIOP beruht wie bei anderen osteoprotektiven Substanzen wieder auf Bridging-Studien, basierend auf Analyse von KMD-Veränderungen [304, 305]; *Evidenzgrad Ib*.

Die EFOS-Studie (European Forsteo Observational Study) hat während einer 18-monatigen Therapie mit Teriparatid und einer anschließenden 18-monatigen Nachbeobachtungsstudie nicht nur die Reduktion von Frakturen im klinischen Alltag unter Therapie bestätigt, sondern auch einen anhaltenden Effekt über zumindest weitere 36 Monate. Neben der Frakturreduktion konnte eine Verbesserung der Lebensqualität, aber auch eine Schmerzreduktion gezeigt werden [306, 307]; *Evidenzgrad IIb*.

Eine randomisierte kontrollierte Vergleichsstudie von Teriparatid mit oralem Risedronat (VERtebral Fracture Treatment Comparison in Osteoporotic Women [VERO]) zeigte eine bessere RRR für Wirbelkörperfrakturen und klinische Frakturen unter Teriparatid. Der Unterschied bei nicht-vertebralen Frakturen war nicht signifikant [308]; *Evidenzgrad Ib*.

Die meisten bioptisch verifizierten Indizes der Knochenformation unter osteoanaboler Therapie mit Teriparatid sind bei Alendronat-vorbehandelten postmenopausalen Patientinnen geringer als bei nicht vorbehandelten Patientinnen [309].

Seit 2020 stehen Teriparatid-Biosimilars in Österreich zur Verfügung, wodurch die Kosteneffizienz deutlich verbessert wurde.

Kontraindikationen für den Einsatz von Teriparatid sind das juvenile Skelett (Alter < 18 Jahre), Schwangerschaft, Stillzeit, vorangegangene maligne Erkrankungen des Skelettsystems oder Bestrahlung, eine fortgeschrittene Nierenfunktionsstörung (GFR < 30 ml/min) und eine bestehende Hyper- oder Hypokalziämie sowie eine unklare Erhöhung der alkalischen Phosphatase.

Zu den typischen Nebenwirkungen zählen Knochenschmerzen und Muskelkrämpfe, Kopfschmerzen, Übelkeit, Schwindel und Orthostaseneigung. Teriparatid kann zu transienten Hyperkalziämien, Hyperkalziurie und Hyperurikämie führen.

##### 8.6.4.2 Abaloparatid (PTHrP 1-34)

Abaloparatid ist ein synthetisches Peptid der Aminosäuren 1‑34 des Parathormon-verwandten Peptids (PTHrP) und ein selektiver Aktivator des PTH1-Rezeptors [310]. Die Zulassung in der Indikation postmenopausale Osteoporose ist 2017 in den USA und 2022 in der EU erfolgt.

Einmal täglich wird eine Dosis von 80 μg subkutan appliziert, und die Therapiedauer ist auf 18 Monate beschränkt. Der osteoanabole Effekt entsteht durch die agonistische Wirkung auf den PTH1-Rezeptor, die zu einer vermehrten Knochenneubildung mit Zunahme der KMD und Knochenfestigkeit führt. Die Zulassungsstudie war die ACTIVE-Studie (Abaloparatide Comparator Trial In Vertebral Endpoints), eine dreiarmige, placebokontrollierte Vergleichsstudie mit Teriparatid [311]; *Evidenzgrad Ib*.

Die Studienpopulation war sehr inhomogen hinsichtlich des Schweregrades der Osteoporose: 24 % der Patientinnen hatten prävalente Frakturen, 31 % periphere Frakturen 5 Jahre vor Studienbeginn, während 37 % der Frauen noch nie eine Fraktur hatten. Während der 18 Monate andauernden Therapie zeigte sich unter Abaloparatid eine signifikante 86 % RRR des Wirbelkörperfrakturrisikos (0,58 % vs. 4,22 %) und eine 43 % RRR von nicht-vertebralen Frakturen (2,7 % vs. 4,7 %) im Vergleich zur Placebogruppe. Im parallel laufenden offenen Therapiearm mit Teriparatid kam es zu einer signifikanten 80 % RRR vertebraler Frakturen und 28 % nicht-vertebraler Frakturen. Zwischen den beiden Therapiegruppen wurde kein signifikanter Unterschied festgestellt [311].

Kontraindikationen und Nebenwirkungen decken sich mit jenen von Teriparatid.

#### 8.6.5 Dual wirksame Medikamente

##### 8.6.5.1 Romosozumab

Empfehlungen:

- Eine dual wirksame Therapie mit Romosozumab ist für postmenopausale Frauen mit einem sehr hohen Frakturrisiko (FRAX®) und einer MOF (hüftnahe Fraktur, klinisch vertebrale Fraktur, Unterarmfraktur, Humerusfraktur) innerhalb der letzten 24 Monate zu erwägen (imminentes Frakturrisiko; s. auch Kap. 6 „Frakturrisiko“); *starke Empfehlung*.
- Romosozumab ist bei postmenopausalen Frauen mit einem sehr hohen Frakturrisiko, die eine osteoanabole Therapie mit Teriparatid oder Abaloparatid nicht vertragen, insbesondere bei Wirbelkörperfrakturen, als Zweitlinienbehandlung in Betracht zu ziehen; *bedingte Empfehlung*.
- Nach einer Behandlungsdauer mit Romosozumab von 12 Monaten soll unverzüglich eine Behandlung mit Alendronat, Zoledronat oder Denosumab eingeleitet werden; *starke Empfehlung*.

Romosozumab ist ein humanisierter monoklonaler Antikörper, der an Sclerostin bindet und dieses hemmt. Er hat eine doppelte Wirkung, indem er die Knochenbildung über Osteoblastenstimulation anregt und die Knochenresorption (Osteoklasten) hemmt. Der Antikörper wurde im Oktober 2019 durch die Europäische Arzneimittelagentur für die Behandlung von schwerer Osteoporose bei postmenopausalen Frauen mit sehr hohem Frakturrisiko zugelassen. Für die Anwendung bei Männern ist die Substanz derzeit nicht zugelassen. Romosozumab wird als subkutane Injektion in einer Dosis von 210 mg (verabreicht als 2 subkutane Injektionen von je 105 mg) 1‑mal monatlich verabreicht. Die Dauer der Behandlung ist auf 12 Monate begrenzt.

Jeder Patient, der die 12-monatige Therapie mit Romosozumab beendet (oder diese zwischenzeitlich absetzen muss), benötigt eine Folgetherapiestrategie, die in der Regel ein antiresorptives Medikament einschließt, welche zum Zeitpunkt der Einleitung der Initialtherapie geplant werden sollte, um eine Behandlungslücke zu vermeiden [312].

Bei postmenopausalen Frauen mit Osteoporose, die Romosozumab 210 mg oder Placebo 1‑mal monatlich für 12 Monate subkutan erhielten, gefolgt von Denosumab 60 mg subkutan in beiden Gruppen für 12 Monate, waren neue Wirbelkörperfrakturen und klinische (nicht-vertebrale und symptomatische vertebrale) Frakturen bei Frauen, die mit Romosozumab behandelt wurden, im Vergleich zu Placebo nach 12 Monaten signifikant reduziert. Nach 24 Monaten war die Inzidenz neuer Wirbelkörperfrakturen bei Frauen, die in den ersten 12 Monaten mit Romosozumab behandelt worden waren, signifikant niedriger [313]; *Evidenzgrad Ib*. Unerwünschte Ereignisse, darunter schwerwiegende kardiovaskuläre Ereignisse, waren zwischen den Behandlungsgruppen ausgeglichen [313]; *Evidenzgrad Ib*.

Bei Frauen mit schwerer Osteoporose und hohem Frakturrisiko zeigte sich im direkten Vergleich von Romosozumab gegenüber Teriparatid eine Verbesserung der KMD (gemessen durch DXA) und der biomechanischen Eigenschaften des Knochens. Dieser Effekt wurde bereits nach 12 Monaten Therapie mit Romosozumab im Gegensatz zur gleichen Therapiedauer mit Teriparatid erreicht [314]; *Evidenzgrad Ib*. Vergleichsdaten zur Frakturreduktion liegen derzeit nicht vor.

In einer aktiv-kontrollierten Studie an postmenopausalen Frauen mit schwerer Osteoporose und prävalenten Frakturen wurde die subkutane Gabe von Romosozumab 210 mg 1‑mal monatlich über 12 Monate, gefolgt von oralem Alendronat 70 mg 1‑mal wöchentlich über 12 Monate, mit Alendronat 70 mg 1‑mal wöchentlich über 24 Monate verglichen. Neue vertebrale, klinische, nicht-vertebrale und hüftnahe Frakturen traten bei Frauen, die mit Romosozumab und anschließend mit Alendronat behandelt wurden, signifikant weniger häufig auf als bei Frauen, die nur mit Alendronat behandelt wurden [315]; *Evidenzgrad Ib*. Über einen Zeitraum von 24 Monaten waren unter Romosozumab im Vergleich zu Alendronat signifikant weniger neue Wirbelkörperfrakturen und klinische (nicht-vertebrale und symptomatische vertebrale) Frakturen aufgetreten. Während des ersten Behandlungsjahres wurden in der Romosozumab-Gruppe in dieser Studie schwerwiegende unerwünschte kardiovaskuläre Ereignisse häufiger beobachtet als in der Alendronat-Gruppe (2,0 % vs. 1,1 %) [315]; *Evidenzgrad Ib*.

Bei Frauen mit schwerer Osteoporose und hohem Frakturrisiko zeigte sich unter Romosozumab im direkten Vergleich zu Teriparatid eine signifikante Zunahme der KMD (gemessen durch DXA) der Hüfte zugunsten von Romosozumab bis Monat 12 (Mittelwert aus Monat 6 und 12). Die Differenz betrug 3,2 % (2,6 vs. −0,6 %). Zur Bestimmung eines Unterschieds im Frakturrisiko unter Romosozumab und Teriparatid war diese Studie jedoch nicht ausgelegt [314]; *Evidenzgrad Ib*.

Romosozumab ist kontraindiziert bei Patientinnen mit Hypokalziämie, Überempfindlichkeit gegen einen der Bestandteile des Präparats oder bei Patienten mit Myokardinfarkt oder Schlaganfall in der Anamnese.

Seit der internationalen Erstzulassung von Romosozumab im Jahr 2019 liegen Daten von mehr als 400.000 behandelten Patientinnen unter Romosozumab-Therapie vor. Die regelmäßige Überprüfung der gemeldeten Ereignisse von Myokardinfarkt und Schlaganfall aus Post-Marketing-Quellen ergab keine neuen Sicherheitsbedenken. Die Meldehäufigkeit von Myokardinfarkten und Schlaganfällen blieb über die Zeit stabil und ist nicht untypisch für ältere Menschen mit Osteoporose, da das zunehmende Alter einen starken klinischen Risikofaktor darstellt [316]; *Evidenzgrad IIIb*.

Bei der Entscheidung, ob Romosozumab bei einer Patientin eingesetzt werden soll, müssen sowohl das Fraktur- als auch das kardiovaskuläre Risiko (auf der Grundlage von Risikofaktoren) für das nächste Jahr der Behandlung berücksichtigt werden. Bei Patientinnen, die Romosozumab erhalten, wurde in vereinzelten Fällen eine transiente Hypokalziämie beobachtet. Eine Hypokalziämie sollte vor Beginn der Behandlung korrigiert werden, und die Patientinnen sollten ausreichend mit Kalzium und Vitamin D versorgt werden. Patientinnen mit schweren Nierenfunktionsstörungen oder Dialysepatientinnen haben ein erhöhtes Risiko, eine Hypokalziämie zu entwickeln. Über Osteonekrose des Kiefers und atypische Femurfrakturen wurde bei der Anwendung von Romosozumab sehr selten berichtet.

### 8.7 Sequenztherapien

Empfehlung:

- Nach einer osteoanabolen Therapie über 24 Monate mit Teriparatid, über 18 Monate mit Abaloparatid oder über 12 Monate mit Romosozumab sollte eine Anschlusstherapie mit Alendronat, Zoledronat oder Denosumab ohne zeitliche Verzögerung eingeleitet werden; *starke Empfehlung*.
- Eine weitere Option als Anschlusstherapie nach osteoanaboler Medikation – jedoch nur bei postmenopausalen Frauen – ist die Gabe von Raloxifen; *bedingte Empfehlung*.

Lediglich die Substanzgruppe der Bisphosphonate hat einen fraktursenkenden Effekt, der über die aktive Therapiedauer hinausgeht. Nach Beendigung einer Bisphosphonat-Therapie dauert der antiresorptive Effekt dieser Substanzklasse bis zu 10 Jahre oder sogar darüber hinaus an. Die einzelnen Bisphosphonate haben aufgrund ihrer unterschiedlichen Bindungsaffinität jedoch eine unterschiedliche Halbwertszeit im Knochengewebe. Zoledronat weist die längste Halbwertszeit auf, gefolgt von Alendronat und Risedronat [317, 318]; *Evidenzgrad Ia*. Dieser theoretische Vorteil einer langen Halbwertszeit im Knochen beinhaltet jedoch auch ein potenzielles Risiko. Denn die jahrelange Verabreichung von Bisphosphonaten kann zu einer Unterdrückung des natürlichen Knochenumbaus führen [319]; *Evidenzgrad Ib*. Mögliche Komplikationen einer verlängerten Bisphosphonat-Therapie werden im Abschn. 8.9. „Nebenwirkungen der Osteoporosetherapie“ beschrieben.

Diese potenziellen Komplikationen rechtfertigen eine Therapiepause („drug holiday“). Ebenso kann Patientinnen, bei denen nach Jahren der Bisphosphonat-Therapie keine Fraktur aufgetreten ist und die inzwischen ein niedriges oder moderates Frakturrisiko ohne prävalente Wirbelkörperfrakturen haben, eine Therapiepause empfohlen werden [320]; *Evidenzgrad Ib*. Eine solche Therapiepause entspricht auch der derzeit gültigen Empfehlung der Task Force der American Society for Bone and Mineral Research [321]; *Evidenzgrad Ia*; (s. auch Kap. 9 „Verlaufskontrollen“ und Abschn. 9.2. „Therapiemonitoring“).

Im Anschluss an diese Therapiepause und in Abhängigkeit von den zuvor verwendeten Substanzen ist eine neuerliche Therapieeinleitung notwendig. Diese kann entweder wieder mit einem Bisphosphonat, mit Denosumab oder nach zwischenzeitlich aufgetretener Fraktur mit einer osteoanabol wirksamen Substanz erfolgen [277]; *Evidenzgrad Ib*.

Im Gegensatz zu Bisphosphonaten ist eine antiresorptive Therapie mit Denosumab keine über den Behandlungszeitraum hinausgehende osteoprotektive Therapie. Daher sollte nach Beendigung der Denosumab-Therapie zeitnah eine Sequenztherapie folgen (s. Abschn. 8.6.3.2. „Denosumab“).

In der Hochrisikosituation (nach stattgehabter Fraktur – imminentes Frakturrisiko, MOF) soll als Erstlinientherapie primär eine osteoanabole bzw. dual wirksame Therapie erwogen werden. Eine mögliche Vortherapie hat jedoch Einfluss auf die Wirksamkeit der dual wirksamen Therapie mit Romosozumab. So zeigte eine Romosozumab-Therapie bei Patientinnen ohne Vortherapie eine KMD-Zunahme von 18,2 %, nach Bisphosphonat-Therapie von 10,2 % und nach Denosumab-Therapie von 6,4 % [322, 323]; *Evidenzgrad Ia*. Auch eine sequenzielle osteoanabole Therapie kann das Outcome der Zweitlinientherapie beeinflussen. So kam es nach vorangegangener Teriparatid-Therapie zu einem KMD-Zuwachs unter Romosozumab von 11,2 %, verglichen mit 18,2 % für therapienaive Patientinnen [323]; *Evidenzgrad III*.

Für Teriparatid hingegen zeigte sich in der VERO-Studie kein Einfluss der Vortherapie auf die fraktursenkende Wirkung [308]; *Evidenzgrad I*. Laut Denosumab-And-Teriparatide-Administration(DATA)-Switch-Studie führt die Sequenz Teriparatid-Denosumab zumindest bezüglich der KMD allerdings zu deutlich höheren Zugewinnen als die Sequenz Denosumab-Teriparatid [324]. Frakturdaten liegen jedoch nicht vor.

### 8.8 Therapie bei sehr hohem Frakturrisiko

Empfehlungen:

- Die Anwendung von FRAX® ermöglicht die Graduierung des individuellen Knochenbruchrisikos in ein geringes, ein mittleres, ein hohes und ein sehr hohes Frakturrisiko; *starke Empfehlung*.
- Teriparatid, Abaloparatid und Romosozumab sind Erstlinienbehandlungen für postmenopausale Frauen mit sehr hohem Frakturrisiko, insbesondere für Frauen mit Wirbelkörperfrakturen; *starke Empfehlung*.
- Teriparatid ist eine Erstlinientherapie bei Männern ab 50 Jahren, die ein sehr hohes Frakturrisiko haben, insbesondere bei Männern mit prävalenten Frakturen; *bedingte Empfehlung*.
- Nach einer osteoanabolen Therapie ist eine sequenzielle Therapie mit antiresorptiven Medikamenten erforderlich; *starke Empfehlung*.

Zwei randomisierte, prospektiv kontrollierte Studien bei postmenopausalen Frauen mit schwerer Osteoporose haben eine überlegene Wirksamkeit von osteoanabolen gegenüber antiresorptiven Medikamenten in Bezug auf Reduktion von Frakturen gezeigt.

In der VERO-Studie war subkutanes Teriparatid 20 µg 1‑mal täglich nach 24-monatiger Behandlung mit signifikant weniger neuen Wirbelkörperfrakturen und klinischen Frakturen effizienter als orales Risedronat 35 mg 1‑mal wöchentlich (56 %, *p* < 0,0001; bzw. 52 %, *p* = 0,0009) [308].

Die subkutane Gabe von Romosozumab 210 mg 1‑mal monatlich führte zu einer signifikant stärkeren Reduktion von Wirbel-, nicht-vertebralen, klinischen und hüftnahen Frakturen nach 24 Monaten (Risikosenkung um 48 %, 19 %, 27 % bzw. 38 %) und zu einer signifikant stärkeren Verringerung des Risikos neuer Wirbel- und klinischer Frakturen nach 12 Monaten im Vergleich zu oralem Alendronat 70 mg 1‑mal wöchentlich [325]; *Evidenzgrad Ib*.

Diese Studien liefern die Begründung für die Erwägung von osteoanabolen oder dual wirksamen Therapien als Erstlinienbehandlung bei postmenopausalen Frauen mit *sehr hohem Frakturrisiko* (s. Abschn. 8.1. „Therapieziele“). Vergleichsstudien zu antiresorptiven und osteoanabolen Wirkstoffen wurden bei Männern nicht durchgeführt. Nach Absetzen der Behandlung mit Teriparatid oder Romosozumab nimmt der Knochenumsatz bzw. der Knochenstoffwechsel zu, und die KMD nimmt wiederum ab. Da die zulässige Höchstdauer der Behandlung mit Teriparatid 24 Monate, mit Abaloparatid 18 Monate und mit Romosozumab 12 Monate beträgt, ist eine sofortige und längerfristige sequenzielle Therapie mit antiresorptiven Medikamenten erforderlich, um die positiven Auswirkungen auf das Skelett und das Knochenbruchrisiko zu erhalten.

Es hat sich gezeigt, dass sowohl durch Alendronat als auch Denosumab die KMD an der Wirbelsäule und der Hüfte nach einer Teriparatid- oder Romosozumab-Therapie erhalten bleibt und weiter zunimmt [324–327]. In der Verlängerungsstudie der FRActure study in postmenopausal woMen with ostEoporosis (FRAME) blieben die positiven Auswirkungen einer 12-monatigen Romosozumab-Therapie auf das vertebrale und nicht-vertebrale Frakturrisiko erhalten, wenn eine sofortige zumindest 24-monatige Denosumab-Behandlung erfolgte [328]; *Evidenzgrad IIb*.

Wenn postmenopausale Frauen von einer oralen Bisphosphonat-Therapie auf eine osteoanabole Therapie mit Teriparatid oder Romosozumab umgestellt werden, kommt es zu einer Abschwächung des Anstiegs der Wirbelsäulen- und Hüft-KMD im Vergleich zur Therapie mit diesen Medikamenten bei therapienaiven Patientinnen. Dieser Abschwächungseffekt ist bei Teriparatid größer als bei Romosozumab, insbesondere an der Hüfte [314, 329]; *Evidenzgrad IIb*. Die Auswirkungen dieser Effekte auf das Frakturrisiko sind, wenn überhaupt, unbekannt.

Bei Frauen, die zuvor mit Denosumab behandelt wurden, ist die Umstellung auf Teriparatid mit einem vorübergehenden Knochenverlust an der Wirbelsäule und einem größeren und länger anhaltenden Knochenverlust an der Hüfte verbunden. Die Auswirkungen auf das Frakturrisiko sind unbekannt [324]. Wenn Romosozumab im Anschluss an eine Denosumab-Therapie verabreicht wird, kommt es zu einer Abschwächung des KMD-Anstiegs an Wirbelsäule und Hüfte [330, 331]; *Evidenzgrad IIb*. Die Auswirkungen auf das Frakturrisiko sind unbekannt.

### 8.9 Nebenwirkungen der Osteoporosetherapie

In den vorigen Kapiteln wurden die häufigsten Kontraindikationen und Nebenwirkungen der jeweiligen osteologischen Medikamente beschrieben. Die nun folgenden 2 Abschnitte befassen sich mit den am häufigsten gestellten Fragen in der täglichen klinischen Praxis und durch Patienten.

#### 8.9.1 Medikamentenassoziierte Kiefernekrose

Das Risiko, unter antiresorptiver Osteoporosetherapie spontan eine medikamentenassoziierte Kiefernekrose (MRONJ) zu entwickeln, liegt bei 1:2000 (0,05 %). Nach invasiven zahnärztlichen Eingriffen steigt das Risiko auf etwa 1:100 (1 %) [332]. Therapien mit Teriparatid, Abaloparatid oder Romosozumab stellen nach derzeitigem Wissensstand kein Risiko für das Auftreten einer MRONJ dar. Im nachfolgenden Kapitel werden daher die Grundlagen der zahnärztlichen Versorgung im Rahmen einer Osteoporosetherapie erörtert.

Empfehlungen:

- Patienten sollten über die Ursachen und das Risiko des Auftretens der MRONJ aufgeklärt werden, und es empfiehlt sich eine zahnärztliche Untersuchung vor Therapiebeginn; *bedingte Empfehlung*.
- Elektive invasive zahnärztliche Eingriffe wie das Setzen dentaler Implantate sind unter Berücksichtigung des erhöhten Risikos einer MRONJ möglich; *bedingte Empfehlung*.
- Notwendige invasive zahnärztliche Eingriffe unter antiresorptiver Osteoporosetherapie sollen unter antibiotischer Abschirmung und speicheldichtem Wundverschluss erfolgen; *starke Empfehlung*.
- Die Gabe von i.v.-Bisphosphonaten sollte frühestens 2 Monate nach invasiven zahnärztlichen Eingriffen beginnen; *bedingte Empfehlung*.
- Invasive Eingriffe sollten gegen Ende eines Behandlungszyklus mit Denosumab durchgeführt und der Behandlungszyklus sollte nach Abheilung der Wunde fortgesetzt werden; *bedingte Empfehlung*.

Die MRONJ wurde 2003 erstmals beschrieben und kann in seltenen Fällen zu einer funktionellen Beeinträchtigung der Kau‑, Schluck- und Sprachfunktion und demnach zu einer Beeinträchtigung der Lebensqualität führen [333, 334]; *Evidenzgrad IIa*. Definitionsgemäß liegt eine MRONJ vor, wenn der Kieferknochen über 8 Wochen freiliegt und eine antiresorptive Therapie in der Anamnese aufscheint sowie eine Kopf-Hals-Radiatio ausgeschlossen werden kann [335]. Das Krankheitsbild wird in 3 Stadien eingeteilt. Die ersten beiden Stadien weisen bereits einen nekrotisch freiliegenden Knochen als Leitbild auf, jedoch sind die Anzeichen der Infektion schwächer als im folgenden Stadium ausgeprägt oder können sogar fehlen. Im schweren 3. Stadium mit exponiertem nekrotischem Knochen können zudem Fisteln und pathologische Frakturen auftreten [335]. Folglich kann eine MRONJ in ihrem Verlauf eine stationäre Behandlung mit intravenöser antiinfektiöser Therapie und der Notwendigkeit chirurgischer Eingriffe nach sich ziehen.

Das Risikoprofil, eine MRONJ zu entwickeln, steht im Zusammenhang mit der unterschiedlichen Dosierung der antiresorptiven Therapie, wobei hier zwischen Patienten mit Osteoporose [332] und onkologischen Indikationen [336] unterschieden werden muss. Beispielsweise liegt das Risiko, unter antiresorptiver Osteoporosetherapie mit Denosumab spontan eine MRONJ zu entwickeln, bei 1:2000 (0,05 %) [332]. Nach invasiven zahnärztlichen Eingriffen steigt das Risiko jedoch auf 0,68 % [332]; *Evidenzgrad Ib*. Zudem werden weitere Risikofaktoren in Betracht gezogen wie die Gabe von Glukokortikoiden, eine Chemotherapie, Rauchen, Diabetes mellitus und immunsuppressive Therapien [337]; *Evidenzgrad IIb*. Zusammenfassend sind das Faktoren, die das Risiko einer Infektion steigern und damit letztlich in Kombination mit der antiresorptiven Therapie die Entwicklung der MRONJ begünstigen [335].

Das Risiko einer MRONJ nach invasiven zahnärztlichen Eingriffen kann durch eine optimale Wundversorgung und Antibiotikagabe reduziert werden. Das Beachten der Pharmakokinetik von Bisphosphonaten und Denosumab ist für die Wahl des Zeitpunktes eines invasiven zahnärztlichen Eingriffes relevant. Intravenös verabreichte Bisphosphonate können bis kurz vor dem zahnärztlichen Eingriff gegeben werden. Bei Denosumab wird ein zahnärztlicher Eingriff am Ende des Therapiezyklus empfohlen. Die erneute Verabreichung einer antiresorptiven Therapie soll grundsätzlich nach Abheilung der Wunde begonnen werden.

Obwohl die Pathogenese nicht vollends geklärt ist, erscheint es auffällig, dass die MRONJ stets mit einer Infektion vergesellschaftet ist. Hypothetisch kommt es durch die Blockierung der Osteoklastogenese und Funktion zu einer Polarisierung der Zellen in Richtung Makrophagen, die durch die bakterielle Kontamination zu einer überschießenden und chronischen Entzündungsantwort neigen und letztlich das Absterben der Knochenzellen, speziell der Osteozyten, verursachen [338, 339]. In diesem entzündlichen Milieu wird der kompromittierte Knochen nicht wie gewünscht abgebaut, sondern bleibt als nekrotischer Knochen erhalten.

Die Hypothese, dass die MRONJ im Zusammenhang mit einer chronischen Infektion ausgelöst wird, bildet zugleich die Grundlage der Prävention. Gemäß der S3-Leitlinie für MRONJ [335] sollen Patienten über die Ursachen und das Risiko des Auftretens der MRONJ aufgeklärt und eine zahnärztliche Untersuchung vor Therapiebeginn soll empfohlen werden; *Evidenzgrad III*. Dabei sollten mögliche Infektionen und Eintrittspforten von Bakterien beseitigt werden: Dazu gehören die Entfernung nicht erhaltungswürdiger Zähne und die Beseitigung entzündlicher Veränderungen in der Mundhöhle. Bei Patienten mit abnehmbarem Zahnersatz ist auf einen guten Prothesensitz zu achten. Auch vonseiten der Hausärztinnen und Hausärzte bzw. Zahnärztinnen und Zahnärzte kann eine klinische Untersuchung der Mundhöhle auf Anzeichen von Entzündung bzw. von freiliegendem Knochen durchgeführt werden. Die S3-Leitlinie für Zahnimplantate bei medikamentöser Behandlung mit Knochenantiresorptiva [340] unterstreicht zudem, dass die Versorgung von Patienten mit dentalen Implantaten unter bzw. nach antiresorptiver Therapie möglich ist; *Evidenzgrad III*. Es sollte jedoch das individuelle Risiko einer MRONJ evaluiert werden inklusive Entzündungsfreiheit der Mundhöhle und Allgemeinerkrankungen sowie systemischer Faktoren, die mit einer erhöhten Wundheilungsstörung assoziiert sein können.

Die hohen Frakturzahlen und die damit einhergehenden erheblichen Mortalitätsraten bei unbehandelter Osteoporose stehen dem vergleichsweise geringen Risiko einer MRONJ nach zahnärztlichen Eingriffen gegenüber. Die unmittelbare Einleitung einer Therapie nach Diagnosestellung der Osteoporose ist demnach wichtig, auch wenn zahnärztliche Eingriffe anstehen. Sollte eine invasive zahnärztliche Behandlung notwendig sein, kann das unter Berücksichtigung der genannten Vorsichtsmaßnahmen jederzeit erfolgen. Es muss bedacht werden, dass Patienten, die mit Denosumab behandelt werden, die Therapieintervalle von 6 Monaten einhalten sollten, da durch den reversiblen Wirkmechanismus des Antikörpers das Frakturrisiko wieder ansteigen würde, wenn die Therapie verzögert oder abgesetzt wird [295]; *Evidenzgrad Ib*. Bei der Gabe von Bisphosphonaten kann die Verabreichung nach invasiven zahnärztlichen Eingriffen um einige Wochen verzögert werden [341, 342]; *Evidenzgrad III*.

Während das optimale Behandlungskonzept der MRONJ noch immer umstritten ist, wurden bereits mehrere ergänzende Therapien bei Osteoporosepatienten eingeführt. Dabei scheint Teriparatid eine vielversprechende Option zu sein. In mehreren Studien wurde die positive Wirkung einer Kurzzeitbehandlung mit Teriparatid beschrieben; sie zeigten eine Verbesserung der Knochenheilung [343]; *Evidenzgrad III*. Eine klinische Validierung durch eine kontrollierte prospektive Studie mit prospektivem Design wäre jedoch wünschenswert [343]. Entsprechende Studien zu Romosozumab oder Abaloparatid liegen nicht vor. Es ist davon auszugehen, dass osteoanabole Therapien grundsätzlich kein Risiko für das Auftreten einer MRONJ bei Osteoporosepatienten darstellen [313, 315]; *Evidenzgrad IV*.

Sollte es trotz prophylaktischer Maßnahmen zum Auftreten einer MRONJ kommen, sollten die nächsten Behandlungsschritte individuell festgelegt werden. Ein Zuwarten sollte wegen des Risikos des asymptomatischen Fortschreitens der MRONJ vermieden werden. Ein konservativer Therapieversuch kann bei kleineren asymptomatischen Fällen erwogen werden. Bei fortgeschrittener MRONJ sollte eine chirurgische Behandlung angedacht werden. Diese Behandlung umfasst die Entfernung des nekrotischen Knochens, die Glättung scharfer Knochenkanten und die plastische Deckung der Wunden. Sämtliche invasiv chirurgischen Maßnahmen sollen unter systemischer antibiotischer Therapie erfolgen [344, 345]; *Evidenzgrad III*.

Zusammenfassend und abschließend ist es wichtig hervorzuheben, dass eine Osteoporosetherapie von zahnärztlicher Seite nicht unterbrochen werden darf, insbesondere nicht bei Denosumab, da nach Unterbrechung das Risiko einer Fragilitätsfraktur steigt. Durch entsprechende Informationen, Aufklärung und Abstimmung der behandelnden Zahnärztinnen und Zahnärzte und der Osteoporosebehandlerinnen und -behandler ist es möglich, Patienten bestmöglich hinsichtlich ihrer „Mundgesundheit“ (einschließlich Extraktionen und Implantationen) *und* ihrer „Knochengesundheit“ zu betreuen.

#### 8.9.2 Atypische Femurfrakturen

Empfehlungen:

- Bei ossären Schmerzen im Bereich des Femurschaftes unter laufender antiresorptiver Therapie sollte eine AFF der ipsi- und der kontralateralen Seite mittels Bildgebung ausgeschlossen werden; *bedingte Empfehlung*.
- Bei subtrochantären Frakturen sollte auf die für AFF typischen kortikalen Verdickungen geachtet werden (Röntgen!), die sowohl bei Therapie mit Bisphosphonaten als auch mit Denosumab auftreten können; *bedingte Empfehlung*.
- Bei Auftreten einer AFF könnte ein Umstieg auf eine osteoanabole Therapie das Outcome verbessern; *bedingte Empfehlung*.
- Die chirurgische Versorgung erfolgt analog der subtrochantären Fraktur, welche meist mit einem Marknagel mit Hüftkomponente versorgt wird. Die Dringlichkeit entspricht jener der hüftnahen Frakturen (< 48 h); *bedingte Empfehlung*.

**Abb. 7:**
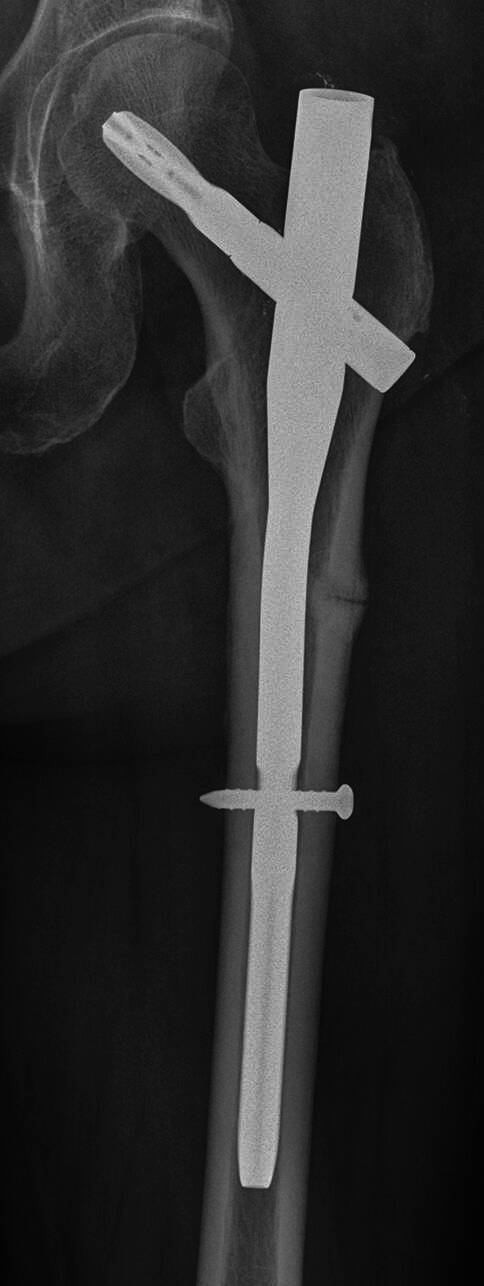
*AFF bei Bisphosphonat-Therapie nach Versorgung mit Marknagel. AFF* atypische Femurfraktur. Typisch ist die kortikale, lippenartige Verdickung um die Fraktur (©Paul Puchwein)

Das Risiko von atypischen Femurfrakturen (AFF) (Abb. 7) bei Einnahme von Bisphosphonaten war in rezenten Studien sehr gering. Auf 75 verhinderte Frakturen gab es eine AFF, erhöht war das Risiko in asiatischen Populationen (1 AFF auf nur 11 verhinderte Frakturen) [346]. Ebenso stieg das Risiko einer AFF unter längerfristiger Bisphosphonat-Therapie kontinuierlich an [346]. Ähnliche Ergebnisse zeigte eine Studie mit knapp 90.000 Patienten mit einer kontinuierlichen Zunahme des AFF-Risikos unter Bisphosphonaten [347]. Bei subtrochantären Frakturen sollte auf die für AFF typischen kortikalen Verdickungen geachtet werden (Röntgen!), ein Auftreten ist sowohl bei Therapie mit Bisphosphonaten als auch mit Denosumab möglich [348]. Jedenfalls ist aber bei ossären Schmerzen im Bereich des Femurschafts unter laufender antiresorptiver Therapie eine AFF der ipsi- und der kontralateralen Seite mittels Bildgebung auszuschließen [349].

Insgesamt ist die Evidenzlage nach stattgehabter AFF bezüglich der medikamentösen Folgetherapie sehr niedrig; *Evidenzgrad IV*. Infrage kommen Teriparatid (unter Monitoring der Knochenumsatzmarker), Weitergabe von Denosumab (speziell, wenn ein Absetzen der Therapie zu weiteren [Wirbelkörper-]Frakturen führen würde) und Raloxifen (Follow-up nach Teriparatid, hohe Knochenumsatzmarker, keine thromboembolischen Ereignisse). Daten aus einer Pilotstudie (*n* = 13) zeigten etwa, dass ein sofortiger Therapiebeginn mit Teriparatid nach operativer Versorgung günstig sein könnte [350].

## 9 Verlaufskontrollen

Die Osteoporose ist eine chronische Erkrankung mit einem dauerhaft erhöhten Frakturrisiko. Abhängig von der jeweiligen Therapie sind regelmäßige zeitliche Verlaufskontrollen mit entsprechenden Befunden Teil des therapeutischen Gesamtkonzeptes.

### 9.1 Therapiedauer

Empfehlungen:

- Eine Osteoporose-spezifische medikamentöse Behandlung sollte nicht beendet werden, da es sonst zu einem Anstieg des Frakturrisikos kommt; *starke Empfehlung*.
- Das Pausieren („drug holidays“) einer erstmaligen Bisphosphonat-Therapie nach 3 Jahren (Zoledronat) oder 5 Jahren (Alendronat, Risedronat) kann nicht empfohlen werden, wenn das Frakturrisiko anhaltend hoch ist oder eine prävalente oder inzidente Fraktur vorliegt; *starke Empfehlung*.
- Die Verlängerung einer 3‑jährigen Zoledronat-Behandlung um weitere 3 Jahre ist mit einer anhaltenden Senkung des Frakturrisikos verbunden; *starke Empfehlung*.
- Die Verlängerung einer 5‑jährigen Alendronat-Behandlung um weitere 5 Jahre ist mit einer anhaltenden Senkung des Frakturrisikos verbunden; *starke Empfehlung*.
- Die Anwendung des SERM Raloxifen kann bis zu einem Zeitraum von 8 Jahren erfolgen; *starke Empfehlung*.
- Die Anwendung von Denosumab über einen Zeitraum von mehr als 10 Jahren kann aufgrund der fehlenden Datenlage nicht empfohlen werden. Es gibt aber umgekehrt auch keine Empfehlung, die Therapie nach 10 Jahren abzusetzen. Sollte ein Absetzen gewünscht und geplant werden, erscheint eine Anschlussbehandlung mit einem anderen osteoprotektiven Medikament sinnvoll; *bedingte Empfehlung*.
- Die Therapiedauer von Teriparatid ist in Europa mit 24 Monaten limitiert, und eine antiresorptive Nachbehandlung ist notwendig; *starke Empfehlung*.
- Die Therapiedauer von Abaloparatid ist mit 18 Monaten limitiert, und eine antiresorptive Nachbehandlung ist notwendig; *starke Empfehlung*.
- Die Therapiedauer von Romosozumab ist mit 12 Monaten limitiert, und eine antiresorptive Nachbehandlung ist notwendig; *starke Empfehlung*.

Die Therapiedauer Osteoporose-spezifischer medikamentöser Therapien wird neben Compliance, Adhärenz und Verträglichkeit durch die zugrunde liegenden Zulassungsstudien sowie durch die im Verlauf einer mehrjährigen Behandlung möglicherweise auftretenden Nebenwirkungen determiniert (s. Kap. 8 „Medikamentöse Therapie der Osteoporose“ und Abb. 4, 5 und 6). Das Pausieren („drug holidays“) oder die vorzeitige Beendigung einer Behandlung mit Bisphosphonaten oder Denosumab ist mit einem Anstieg des Frakturrisikos verbunden [286]; *Evidenzgrad IIa*.

#### 9.1.1 Bisphosphonate

Bisphosphonate persistieren für mehrere Jahre nach der Anwendung im knöchernen Skelett. Dies hat zur Überlegung geführt, dass der positive Effekt auch während einer Unterbrechung oder nach Beendigung einer derartigen Behandlung andauern könnte [351]. Generell umfasst die Mehrheit aller randomisierten, kontrollierten Phase-III-Zulassungsstudien für postmenopausale Frauen eine Bisphosphonat-Therapiedauer von 3 Jahren [321, 352].

Therapieempfehlungen, welche über diesen Zeitraum hinausgehen, basieren daher auf limitierter Evidenz von Verlängerungsarmen der jeweiligen Zulassungsstudien; *Evidenzgrad IIa*. Für Männer stehen über einen Zeitraum von 3 Jahren keine randomisierten kontrollierten Zulassungsstudien Osteoporose-spezifischer Medikamente zur Verfügung. Die einzige randomisierte kontrollierte Studie mit primärem Frakturendpunkt beim männlichen Geschlecht basiert auf Zoledronat 1‑mal jährlich für einen Beobachtungszeitraum von 2 Jahren [268].

Wird eine mehrjährige Behandlung mit Alendronat beendet, sinkt die KMD innerhalb von 2 bis 3 Jahren, und der Knochenumsatz steigt [353, 354]; *Evidenzgrad Ib*. Sinngemäß Gleiches ist für Risedronat und Ibandronat, allerdings bereits nach 1 bis 2 Jahren, zu erwarten [355, 356]; *Evidenzgrad Ib*. Im Gegensatz dazu ist 3 Jahre nach Beendigung einer 3‑jährigen Therapie mit Zoledronat nur eine minimale Abnahme der KMD zu beobachten [357]; *Evidenzgrad Ib*.

Die Fortsetzung einer 5‑jährigen Behandlung mit Alendronat für weitere 5 Jahre ist mit einem signifikant niedrigeren vertebralen Frakturrisiko assoziiert, verglichen mit einer 5‑jährigen Behandlung mit Alendronat gefolgt von 5 Jahren Placebobehandlung [354]; *Evidenzgrad Ib*. Eine 3‑jährige Behandlung mit Zoledronat gefolgt von weiteren 3 Jahren Behandlung ist mit einem signifikant geringeren vertebralen Frakturrisiko verbunden, verglichen mit 3 Jahren Zoledronat-Behandlung und nachfolgend 3 Jahren Placebobehandlung [357]; *Evidenzgrad Ib*.

Den größten Nutzen einer Fortsetzung der Bisphosphonat-Therapie über die empfohlene Mindestbehandlungsdauer haben Personen, welche bereits eine prävalente Fraktur hatten, eine oder mehrere Frakturen während der ersten 3 bis 5 Therapiejahre erlitten oder einen KMD-T-Score von < −2,0 (Alendronat) oder < −2,5 (Zoledronat) aufweisen [358]; *Evidenzgrad Ib*.

#### 9.1.2 Selektive Östrogenrezeptormodulatoren

Für Raloxifen liegen Daten betreffend die Anwendungssicherheit bis zu einer Behandlungsdauer von 8 Jahren vor, darüber hinaus Knochenbiopsien von unauffälliger Histomorphometrie an einer geringen Anzahl von Patientinnen [359, 360]; *Evidenzgrad Ib*. Daten, aus welchen eine darüber hinausgehende Behandlungsdauer abgeleitet werden könnte, stehen bislang nicht zur Verfügung.

#### 9.1.3 Denosumab

Die Beendigung oder Unterbrechung einer Denosumab-Behandlung führt innerhalb von 2 Jahren zu einem vollständigen Verlust der unter Behandlung angestiegenen KMD [282]; *Evidenzgrad Ib*. Darüber hinaus zeigt sich v. a. bei Personen mit prävalenten Frakturen bei einer Therapiebeendigung nach Verabreichung von nur 2 Dosierungen ein Anstieg des Risikos für multiple vertebrale Frakturen [361]. Es stehen gegenwärtig keine Daten zur Verfügung, welche eine Denosumab-Behandlungsdauer von mehr als 10 Jahren unterstützen würden. In jedem Fall sollte bei Therapiebeendigung eine antiresorptive Therapie mit Bisphosphonaten eingeleitet werden [295]; *Evidenzgrad Ib*.

#### 9.1.4 Teriparatid

Die mediane Therapiedauer von Teriparatid betrug im Rahmen der Zulassungsstudie 21 Monate [301]. Eine Beendigung der Therapie ohne nachfolgende antiresorptive Therapie führt zu einem Verlust der während der Behandlung angestiegenen KMD, der positive Effekt auf das nicht-vertebrale Frakturrisiko bleibt jedoch erhalten [362]; *Evidenzgrad IIa*. In der Regel beträgt der Behandlungszeitraum 24 Monate, gefolgt von einer antiresorptiven konsolidierenden Anschlussbehandlung mit einem Bisphosphonat oder Denosumab. Für eine Fortsetzung der Therapie über einen Zeitraum von 24 Monaten hinausgehend stehen bislang keine entsprechenden Daten zur Verfügung. Bislang gibt es beim Menschen keinen Hinweis auf ein erhöhtes Osteosarkomrisiko unter oben genannter Behandlungsdauer [300]; *Evidenzgrad IIa*.

#### 9.1.5 Abaloparatid

Ähnlich wie für Teriparatid liegen Daten aus Tiermodellen für ein erhöhtes Osteosarkomrisiko bei hoch dosierter Langzeitanwendung vor [363]. Die Zulassungsstudie ACTIVE mit dem aktiven Komparator Teriparatid war für einen Behandlungszeitraum von 18 Monaten ausgelegt. In der Praxis wird Abaloparatid daher analog zu Teriparatid über einen Zeitraum von 18 Monaten angewandt, gefolgt von einer antiresorptiven Anschlussbehandlung. Letztere erfolgte im 2‑jährigen Verlängerungsarm der Zulassungsstudie mit der 1‑mal wöchentlichen Dosierung von Alendronat [311, 364].

#### 9.1.6 Romosozumab

Die optimale Behandlungsdauer von Romosozumab ist durch die entsprechende Zulassungsstudie mit 12 Monaten definiert, gefolgt von einer 12-monatigen antiresorptiven Anschlussbehandlung mit Denosumab [313]. Es stehen keine Daten zur Verfügung, welche eine längere Anwendung von Romosozumab oder die Dauer der Anschlussbehandlung unterstützen würden. Es gibt Studien, welche den positiven Effekt einer neuerlichen Gabe von Romosozumab bei Patientinnen mit persistierend hohem bzw. neuerlich hohem Risiko nach einer zwischengeschalteten antiresorptiven Therapie belegen [330, 365].

### 9.2 Therapiemonitoring

Empfehlungen:

- Eine Überprüfung der Adhärenz bei Männern und Frauen, die während einer medikamentösen Behandlung eine Fragilitätsfraktur erleiden, wird empfohlen; *starke Empfehlung*.
- Die Bewertung des Frakturrisikos bei Patienten, die eine medikamentöse Behandlung erhalten, sollte anhand von FRAX® mit KMD erfolgen, wobei die FRAX®-Wahrscheinlichkeiten rechnerisch angepasst werden sollen, um zusätzlichen klinischen Risikofaktoren Rechnung zu tragen (s. Abschn. 3.1. „Klinische Risikofaktoren“). Wenn die von FRAX® abgeleitete Frakturwahrscheinlichkeit den Interventionsschwellenwert überschreitet, soll die medikamentöse Behandlung fortgesetzt werden; *starke Empfehlung*.
- Wenn unter antiresorptiver Therapie biochemische Marker des Knochenumsatzes einen Wiederanstieg des unterdrückten Knochenumsatzes anzeigen und/oder die KMD nach dem Absetzen von Bisphosphonaten absinkt, sollte die Wiederaufnahme der medikamentösen Behandlung erwogen werden; *bedingte Empfehlung*.
- Nach 10 Jahren Bisphosphonat- oder Denosumab-Behandlung soll das Management der Patienten auf individueller Basis geprüft werden; *starke Empfehlung*.

Das Absetzen einer Osteoporosebehandlung, sei es mit einem Bisphosphonat oder Denosumab, ist mit einem erhöhten Risiko für Fragilitätsfrakturen verbunden, sodass eine routinemäßige Beendigung der antiresorptiven Therapie (Therapiepause, „drug holidays“) evidenzbasiert nicht unterstützt wird [286]; *Evidenzgrad IIa*.

Eine Neubewertung des Frakturrisikos bei behandelten Personen kann mithilfe von FRAX® mit KMD-Messung am Schenkelhals durchgeführt werden [366]; *Evidenzgrad IIb*. Die Interventionsschwelle kann dann als Entscheidungshilfe für die Frage dienen, ob die Behandlung für einen bestimmten Zeitraum unterbrochen werden kann oder fortgesetzt werden muss (Abb. 5, 6 und 7). FRAX® kann zwar nicht zur Beurteilung des Ansprechens auf die Behandlung verwendet werden, spielt aber eine Rolle bei der Neubewertung des aktuellen Frakturrisikos, um zu entscheiden, ob die Behandlung fortgesetzt oder abgesetzt werden muss [366]; *Evidenzgrad IIb*.

Die Erkennung einer Verringerung der medikamentösen Wirkung anhand von Veränderungen der KMD und des Knochenumsatzes liefert potenziell Informationen, die das klinische Management beeinflussen können. Derzeit gibt es jedoch keine definitiven Daten, die eine potenzielle Schwellenwertänderung der KMD oder der Knochenumsatzmarker während des Abklingens der Arzneimittelwirkung mit klinisch bedeutsamen Änderungen des Frakturrisikos in Verbindung bringen [367]; *Evidenzgrad Ia*.

### 9.3 Therapieversagen

Empfehlungen:

- Ein Therapieversagen sollte in Betracht gezogen werden, wenn unter einer antiresorptiven Therapie über einen Zeitraum von mindestens 12 Monaten bei adäquater Adhärenz und Ausschluss sekundärer Ursachen 2 oder mehrere inzidente MOF auftreten; *bedingte Empfehlung*.
- Ein Therapieversagen sollte in Betracht gezogen werden, wenn unter einer antiresorptiven Therapie über einen Zeitraum von mindestens 12 Monaten bei adäquater Adhärenz und Ausschluss sekundärer Ursachen ein signifikanter Abfall der KMD beobachtet wird; *bedingte Empfehlung*.
- Ein Therapieversagen sollte in Betracht gezogen werden, wenn unter einer antiresorptiven Therapie über einen Zeitraum von mindestens 12 Monaten bei adäquater Adhärenz und Ausschluss sekundärer Ursachen biochemische Marker des Knochenumsatzes nicht erwartungsgemäß supprimiert sind; *bedingte Empfehlung*.
- Ein Therapieversagen führt in den meisten Fällen zu einer Änderung der laufenden Osteoporosetherapie; *bedingte Empfehlung*.

Eine konsensuelle Definition des Begriffs „Therapieversagen“ steht derzeit nicht zur Verfügung. Weitgehend akzeptiert ist jedoch, dass ein Therapieversagen dann in Betracht gezogen werden sollte, wenn 2 oder mehr inzidente MOF bei Patienten auftreten, welche zumindest über 12 Monate bei nachweislich guter Compliance und Ausschluss sekundärer Ursachen mit einer antiresorptiven Substanz behandelt wurden [368]; *Evidenzgrad IV.* Eine schlechte Adhärenz liegt vor, wenn < 80 % der Behandlung korrekt eingenommen wurden. Sinngemäß kann ein Therapieversagen auch dann angenommen werden, wenn unter den genannten Therapiebedingungen ein signifikanter Verlust der KMD oder fehlende Suppression von biochemischen Markern des Knochenumsatzes beobachtet wird; *Evidenzgrad IV*. Diese Einschätzungen basieren auf dem Ergebnis einer Arbeitsgruppe des Wissenschaftsbeirates (Committee of Scientific Advisors [CSA]) der IOF. Es wird dem Umstand Rechnung getragen, dass auch bei adäquater Therapie-Compliance Frakturen während der Behandlung auftreten können, ebenso wie ein inadäquater oder fehlender Abfall der biochemischen Marker des Knochenumsatzes oder ein signifikanter Abfall der KMD.

#### 9.3.1 Mögliche Konsequenzen eines Therapieversagens

In den meisten Fällen führt ein Therapieversagen zu einer Änderung der laufenden Osteoporosetherapie [369]; *Evidenzgrad IV*. Eine Möglichkeit ist, eine antiresorptive Therapie durch eine andere antiresorptive Therapie zu ersetzen. Untersucht ist beispielsweise der Ersatz einer mehrjährigen Therapie mit Alendronat durch Denosumab oder Zoledronat [370]. Dabei zeigte sich in der mit Denosumab behandelten Gruppe ein signifikant höherer Anstieg der KMD im Vergleich zu Zoledronat [370]; *Evidenzgrad Ib*. Demnach führt Denosumab nach mehrjähriger oraler antiresorptiver Vorbehandlung mit Alendronat im Vergleich zu Zoledronat zu einem signifikant höheren KMD-Zuwachs. Der Effekt auf das Frakturrisiko war zwar tendenziell, jedoch nicht signifikant ausgeprägter [370]; *Evidenzgrad Ib*. Basierend auf den Datenanalysen von Medicare-Versicherten in den Vereinigten Staaten, zeigt sich eine signifikant bessere Frakturreduktion unter Denosumab im Vergleich zur Zoledronat-Therapie [371].

Eine andere Möglichkeit besteht darin, eine antiresorptive Therapie durch eine osteoanabole oder dual wirksame Therapie zu ersetzen; *Evidenzgrad IV*. Grundsätzlich sind die Effekte einer osteoanabolen Therapie im Anschluss an eine antiresorptive Therapie geringer ausgeprägt als bei nicht vorbehandelten Patienten [372–374]; *Evidenzgrad Ib*. Weiters zeigen Studien, dass eine osteoanabole Therapie mit Teriparatid nach mehrjähriger antiresorptiver Vorbehandlung initial sogar zu einer Abnahme der KMD führen kann [314, 375]; *Evidenzgrad Ib*. Dieser initiale KMD-Abfall bedeutet aber nicht, dass das Medikament nicht wirkt.

Demgegenüber stehen die Ergebnisse der VERO-Studie, in welcher gezeigt wurde, dass nach mehrjähriger Bisphosphonat-Vorbehandlung eine Behandlung mit Teriparatid zu einer Frakturrisikoreduktion führt, welche vergleichbar mit jener bei nicht vorbehandelten Patienten ist [308, 376]; *Evidenzgrad Ib*. Die DATA-Switch-Studie ergab, dass ein Wechsel von Denosumab auf Teriparatid zu einem Anstieg der biochemischen Marker des Knochenumsatzes sowie zu einem Anstieg der KMD an der Lendenwirbelsäule führt, dies aber begleitet wird von einer vorübergehenden Abnahme der KMD an der Hüfte [324]; *Evidenzgrad Ib*. Obwohl die Studie nicht gepowert war, um signifikante Unterschiede in der Frakturrisikoreduktion zu erfassen, kann davon ausgegangen werden, dass das Frakturrisiko zumindest in der Initialphase nach Therapiewechsel erhöht sein könnte [377, 378]; *Evidenzgrad IV*. Es wird daher empfohlen, die Vorbehandlung mit Denosumab während der gesamten (oder einem Teil der) osteoanabolen Therapiedauer mit Teriparatid (oder Abaloparatid) beizubehalten; *Evidenzgrad IV*. Diese Empfehlung stützt sich auf Ergebnisse der DATA-Studie, in welcher gezeigt wurde, dass die kombinierte Anwendung von Denosumab und Teriparatid zu ähnlichen KMD-Zuwächsen führt wie eine Teriparatid-Monotherapie [379]; *Evidenzgrad Ib*.

In der STudy evaluating the effect of RomosozUmab Compared with Teriparatide in postmenopaUsal women with osteoporosis at high risk for fracture pReviously treated with bisphosphonatE therapy (STRUCTURE) wurde bei postmenopausalen Frauen der Effekt einer Therapie mit Romosozumab oder Teriparatid im Anschluss an eine vieljährige Therapie mit Bisphosphonaten untersucht [314]. Zuwächse der KMD fielen sowohl für Romosozumab als auch für Teriparatid schwächer aus, als aus Studien mit nicht vorbehandelten Patienten zu erwarten gewesen wäre. Nichtsdestotrotz war der KMD-Zuwachs bei Patienten, welche mit Romosozumab behandelt wurden, signifikant höher als bei jenen, welche mit Teriparatid behandelt wurden; *Evidenzgrad Ib*. Die osteoanabole Behandlungsdauer betrug allerdings nur 12 Monate, weswegen ein Vergleich mit Teriparatid de facto wissenschaftlich nicht fundiert ist. Die empfohlene Behandlungsdauer für Letzteres beträgt 24 Monate, und der maximale Effekt ist daher nicht vor Ende dieser Behandlungsdauer zu erwarten; *Evidenzgrad Ib*.

## 10 Strukturierte Versorgung

### 10.1 Koordinierte Betreuungsmodelle

Empfehlungen:

- Koordinierte Betreuungsmodelle wie das FLS stellen einen systematischen, strukturierten Zugang dar, um Frakturpatienten zu identifizieren und entsprechende diagnostische und therapeutische Maßnahmen zu setzen; *starke Empfehlung*.
- Bei Frakturpatienten ≥ 50 Jahre sollen eine Abschätzung des Sturzrisikos sowie mittels FRAX® des Frakturrisikos und der Ausschluss sekundärer Ursachen für Osteoporose erfolgen; *starke Empfehlung*.
- Die Diagnose „Osteoporose“ soll im Entlassungsdokument angeführt werden; *starke Empfehlung*.
- Eine KMD-Messung mittels DXA und ein seitliches Röntgen der Brust- und Lendenwirbelsäule werden empfohlen, soll die Therapieinitiierung aber nicht verzögern; *starke Empfehlung*.
- Ergänzende nicht-pharmakologische Interventionen, um Stürze und Frakturen zu verhindern, sind empfohlen; *starke Empfehlung*.
- Die Wahl der pharmakologischen Therapie soll die Möglichkeit einer guten Adhärenz berücksichtigen und auch im Verlauf überprüft werden; *starke Empfehlung*.

Koordinierte Betreuungsmodelle wie das Fracture Liaison Service (FLS, Tab. 9) stellen einen systematischen, strukturierten und international empfohlenen Zugang dar, um Therapie zu induzieren, Adhärenz zu verbessern und Folgefrakturen zu verhindern [380, 381]; *Evidenzgrad Ia***.**

**Tab. 9:** Konzept des FLS im Krankenhaus

**1.** ***Identifizierung von Frakturpatienten***	
– Frauen und Männer ≥ 50 Jahre	
**2.** ***Evidenzbasierte Bewertung***	
– Risikostratifizierung (FRAX®-basiert)	
– Identifizierung sekundärer Ursachen der Osteoporose	
– Bildgebung der Wirbelsäule	
– DXA	
**3.** ***Initiierung einer Therapie***	
– Pharmakologische Therapie	
– Nicht-pharmakologische Interventionen (z. B. Sturzprävention, Muskelaufbautraining, Lebensstilmodifikationen [Alkohol, Nikotin, Ernährung]; s. auch Kap. 7 „Nicht-medikamentöse Therapie der Osteoporose“)	
**4.** ***Überprüfung der Therapietreue***	
– Nach 12 bis 16 Wochen und nach einem Jahr	

*DXA* 2-Spektren-Röntgenabsorptiometrie, *FLS* Fracture Liaison Service, *FRAX* Fracture Risk Assessment Tool

Modifiziert nach [382, 383] sowie nach Flowchart Abschn. 9.2 „Therapiemonitoring“

Mittels FLS können jedoch nicht nur Folgefrakturen, sondern es kann auch die Mortalität der Patienten reduziert werden [384]; *Evidenzgrad Ia*. FLS-Systeme sind darüber hinaus kosteneffizient oder sogar kostensparend [385]; *Evidenzgrad IIa*.

FLS erfordert ein multidisziplinäres Setting und wird von FLS-Koordinatorinnen und FLS-Koordinatoren in einem Krankenhaus betreut. Ziel ist die Identifizierung von stationären und ambulanten Frakturpatienten, die osteologische Abklärung und der Beginn einer pharmakologischen und nicht-pharmakologischen antiosteoporotischen Therapie.

### 10.2 Empfehlungen für den niedergelassenen Bereich

Empfehlungen:

- Jede durch ein inadäquates Trauma verursachte Fraktur ist nach Ausschluss anderer Ursachen als osteoporotische Fraktur zu klassifizieren; *starke Empfehlung*.
- Jeder Patient ≥ 50 Jahre mit einem klinischen Risikofaktor ist im Sinne der Primärprävention mittels FRAX® und ggf. mittels zusätzlicher KMD-Messung auf eine Osteoporose mit erhöhtem Frakturrisiko abzuklären; *starke Empfehlung*.
- Jede Osteoporose mit erhöhtem Frakturrisiko ist zum frühestmöglichen Zeitpunkt zu behandeln; *starke Empfehlung*.
- Die Behandlung der Osteoporose umfasst medikamentöse und nicht-medikamentöse Maßnahmen; *starke Empfehlung*.
- Bei hohem Frakturrisiko ist primär eine antiresorptive Therapie einzuleiten; *starke Empfehlung*.
- Bei sehr hohem Frakturrisiko ist primär eine osteoanabole Therapie einzuleiten; *starke Empfehlung*.

Laut WHO gehört die Osteoporose zu den 10 häufigsten Krankheiten weltweit [8]. Daraus ergibt sich, dass Diagnose und Therapie der Osteoporose nicht alleine die Aufgabe von spezialisierten Zentren sein können. Vielmehr hat die Versorgung dieser Patienten vorrangig im niedergelassenen Bereich zu erfolgen.

Die Basistherapie sollte bereits vor Eintreten einer Osteoporose beginnen. Dies vor dem Hintergrund, dass ein wesentlicher Anteil der Frakturen erfolgt, bevor eine Osteoporose diagnostiziert worden ist. Die adäquate Versorgung mit Kalzium und Vitamin D ist Basis jeder Osteoporosebehandlung. Zur Verbesserung der Adhärenz sollte einem Kombinationspräparat der Vorzug gegeben werden. Die Resorption v. a. von Vitamin D wird durch die Einnahme zusammen mit einer fetthaltigen Mahlzeit verbessert.

Vorsorgeuntersuchungen sollten dafür genutzt werden, klinische Risikofaktoren für eine Osteoporose zu evaluieren bzw. eine Osteoporose zu diagnostizieren und ggf. weitere Maßnahmen einzuleiten. Allgemeine Gültigkeit, nicht nur zur Prävention einer Osteoporose, haben Empfehlungen bezüglich eines gesunden Lebensstils. Ein Rauchstopp ist zu empfehlen, begleitet von einer gesunden Ernährung sowie ausreichender körperlicher Bewegung zur Förderung von Kraft, Ausdauer, Gleichgewicht und Koordination.

Ebenso ist das häusliche Umfeld auf Risikofaktoren zu prüfen. Gängige Stolperfallen sind beispielsweise Teppiche ohne Rutschgitter oder schlecht beleuchtete Stufen. Hier sind v. a. Ärztinnen und Ärzte, die Hausbesuche durchführen, gefordert. Denn eine effektive Sturzprophylaxe führt zu einer effektiven Prophylaxe gegen Knochenbrüche.

In der Primärprävention kommt FRAX® zur Anwendung. Es gibt wesentliche Neuerungen im Vorgehen:

- Lässt sich aus FRAX® ein niedriges Risiko für eine Fraktur ableiten, sind keine weiterführenden Maßnahmen indiziert.
- Ergibt sich aus FRAX® ein hohes Risiko, ist eine antiresorptive Therapie einzuleiten.
- Ergibt sich aus FRAX® ein sehr hohes Risiko, ist eine osteoanabole Therapie einzuleiten.
- Nur bei einem Risiko im intermediären Bereich (zwischen der unteren und der oberen Interventionsschwelle, s. auch Kap. 6 „Frakturrisiko“) ist die Durchführung einer KMD-Messung, wenn möglich in Kombination mit einem TBS, notwendig. Entsprechend dem Ergebnis ergibt sich die therapeutische Konsequenz.

Bei jedem Knochenbruch nach inadäquatem Trauma ist nach Ausschluss anderer Ursachen die Diagnose einer Osteoporose zu stellen. Jede Osteoporose ist ohne Zeitverzögerung zu behandeln. Zu beachten sind Laboruntersuchung vor Therapiebeginn (s. Abschn. 5.5 „Osteologisches Labor“), allfällige Indikationen und Kontraindikationen (s. Kap. 8 „Medikamentöse Therapie der Osteoporose“) sowie regelmäßige Kontrolluntersuchungen und mögliche Sequenztherapien (s. Kap. 9 „Verlaufskontrollen“).

Auch nach stattgehabter Fraktur sind nicht-medikamentöse Maßnahmen und Sturzprävention wesentliche Bestandteile einer umfassenden Versorgung. Die Osteoporose als chronische Erkrankung bedarf einer lebenslangen Behandlung.

### 10.3 Optimierung der Osteoporoseversorgung im Gesundheitssystem

Die folgenden Empfehlungen richten sich an die Entscheidungsträger und Institutionen im österreichischen Gesundheitssystem. Osteoporose ist eine sehr häufige Erkrankung mit einer hohen jährlichen Frakturinzidenz. Eine Erhebung des Frakturrisikos vor der ersten Fraktur ist daher notwendig, ebenso muss die Behandlungslücke nach einer osteoporotischen Fraktur optimiert werden, im Idealfall durch eine verbesserte Koordination zwischen den regionalen und überregionalen Entscheidungsträgern und Institutionen im österreichischen Gesundheitssystem, Ärztinnen und Ärzten und weiteren Betreuungsdiensten.

Empfehlungen:

- Osteoporose-bedingte Frakturen stellen ein bedeutendes und wachsendes nationales Problem für die öffentliche Gesundheit dar, das hohe Kosten für die Gesundheits- und Sozialfürsorge nach sich zieht. Fragilitätsfrakturen sollten daher in einem nationalen Gesundheitsprogramm ausdrücklich berücksichtigt werden; *starke Empfehlung*.
- Gesundheitsprogramme sollten Ansätze zur Verringerung vermeidbarer Risikofaktoren für Osteoporose und für sturzbedingte Frakturen berücksichtigen; *starke Empfehlung*.
- Elektronische Patientendatensysteme sollten FRAX® integrieren, um die Identifizierung und Behandlung von Personen mit einem erhöhten Frakturrisiko zu unterstützen. Elektronische Patientendatensysteme sollten eine klare und möglichst automatisierte elektronische Kommunikation zwischen FLS und Primärversorgungsteams zur Therapieeinleitung und -überwachung ermöglichen; *starke Empfehlung*.
- Vorkehrungen sollten getroffen werden, damit Personen mit einem erhöhten Risiko für osteoporotische Frakturen die Möglichkeit haben, geeignete Untersuchungen (z. B. Frakturrisikoabschätzung mittels FRAX®, Sturzrisikoabschätzung, KMD-Messung), Ratschläge zur Lebensführung (z. B. zu Ernährung, Bewegung und Rauchen) und eine präventive knochenspezifische medikamentöse Therapie zu erhalten; *starke Empfehlung*.
- Die routinemäßige Verankerung der Osteoporose in Früherkennungs- bzw. Vorsorgeuntersuchungen ist notwendig, da die Erkrankung eine hohe Prävalenz, Morbidität und Mortalität hat und das Gesundheitssystem stark belastet; *starke Empfehlung*.
- In der Primärprävention sollte eine Osteoporose-spezifische Anamneseerhebung vergütet werden und ggf. eine Zuweisung zu einer KMD-Messung und zusätzlich zu einer bildgebenden Untersuchung erfolgen; *starke Empfehlung*.
- In der Sekundärprävention sollte der niedergelassene Bereich in die Nachsorge eingebunden (regelmäßige Kontrolle, Reevaluierung der Therapie und Medikamentenadhärenz, klinische Risikofaktoren sowie ggf. Labor, Bildgebung, KMD-Messung) und die Leistungen sollten entsprechend vergütet werden; *starke Empfehlung*.
- Integrierte Versorgungssysteme wie ein FLS (s. Abschn. 10.1. „Koordinierte Betreuungsmodelle“) sollten für alle Patienten, die eine Fragilitätsfraktur erleiden, zur Verfügung stehen; *starke Empfehlung*.
- Integrierte Versorgungssysteme sollten aus einem Netzwerk von Allgemeinmedizinern, Spezialisten und anderen Gesundheitsberufen sowie Patientenvertretern bestehen. Ein solches Netzwerk soll Behandlungsmöglichkeiten und Überweisungswege in der Primär- und Sekundärprävention der Osteoporose sicherstellen. Ein verantwortlicher Experte zur leitlinienkonformen Umsetzung und Einhaltung der Primär- und/oder Sekundärpräventionsmaßnahmen bei Osteoporose sollte etabliert werden; *starke Empfehlung*.

Entscheidungsträger und Institutionen im österreichischen Gesundheitssystem sowie deren ausführende Organe und auch medizinisches Fachpersonal, die die Bevölkerung hinsichtlich der Prävention von Stürzen und Fragilitätsfrakturen sowie deren adäquate Behandlung beraten und aufklären, sollten sich auf diese 3 Prioritäten für die Optimierung der aktuellen Situation in Österreich konzentrieren:

1. Sturzprävention,
2. Erkennung und Behandlung der Erkrankung Osteoporose vor der ersten Fraktur,
3. optimale Unterstützung und Behandlung nach einer Fragilitätsfraktur.

Rezente Daten aus Österreich zeigen, dass die Erkrankung Osteoporose und die jährliche Anzahl der Fragilitätsfrakturen in Österreich sehr hoch ist (s. Kap. 2 „Epidemiologie“). Trotz erlittener Fraktur wird in Österreich die überwiegende Mehrheit der Patienten nicht adäquat medikamentös behandelt [386]. Osteoporose hat im Vergleich zu anderen europäischen Ländern derzeit keine nationale Priorität trotz hoher Kosten für das Gesundheitssystem und einer individuellen Belastung für den einzelnen Patienten [8, 21, 386, 387]; *Evidenzgrad Ia*.

Daten aus anderen europäischen Ländern mit integrierten und strukturierten Versorgungssystemen zeigen klar, dass verbesserte Versorgungspfade für Patienten mit einem erhöhten Fragilitätsrisiko Vorteile für den Patienten und das Gesundheitssystem bringen. Die routinemäßige Verankerung einer Leistungserbringung inklusive Remuneration für die Anamnese (Frakturen, klinische Risikofaktoren) und beispielsweise direkte Überweisungen zwischen verschiedenen Diensten der Primär- und der Sekundärversorgung straffen Versorgungspfade von Patienten mit klar belegten Vorteilen, da Therapien schneller eingeleitet werden [388, 389]; *Evidenzgrad Ia*.

## 11 Zusammenfassung

Die vorliegende Leitlinie der Österreichischen Gesellschaft für Knochen- und Mineralstoffwechsel (ÖGKM) umfasst alle Aspekte der Osteoporose und osteoporotischer Frakturen. Die Empfehlungen und die zugrunde liegende Literatur umfassen auch die sekundären Ursachen der Osteoporose, ihre Prävention und Diagnose, die Erfassung der 10-Jahres-Frakturwahrscheinlichkeit mittels FRAX®, die Ermittlung FRAX®-basierter Österreich-spezifischer Interventionsschwellen, medikamentöse und nicht-medikamentöse Therapieoptionen sowie Möglichkeiten des Therapiemonitorings. Die Empfehlungen für den niedergelassenen Bereich sowie für die Entscheidungsträger und Institutionen im österreichischen Gesundheitssystem berücksichtigen strukturierte Versorgungsmodelle sowie Möglichkeiten zur gezielten Vorsorge. Diese Leitlinie erlaubt es Ärztinnen und Ärzten aller Fachrichtungen sowie Entscheidungsträgern und Institutionen im österreichischen Gesundheitssystem, die Qualität der Versorgung von Personen mit Osteoporose oder osteoporotischen Frakturen in allen Ebenen des österreichischen Gesundheitswesens entscheidend zu verbessern.

## Appendix

### Appendix A

**Tab. A.1:** Abkürzungsverzeichnis

Abkürzung	Definition
3D	Dreidimensional
AAC	Abdominal aortic calcification
ABCSG	Austrian Breast & Colorectal Cancer Study Group
ACR	American College of Rheumatology
ADL	Alltagsaktivitäten, activities of daily living
AFF	Atypische Femurfraktur
AHRQ	Agency for Healthcare Research and Quality, National Guideline Clearinghouse
AIDS	Akquiriertes Immundefizienzsyndrom
AO	Arbeitsgemeinschaft für Osteosynthesefragen
AP	Alkalische Phosphatase
AWMF	Arbeitsgemeinschaft der wissenschaftlichen medizinischen Fachgesellschaften
BMI	Body Mass Index
BSG	Blutsenkungsgeschwindigkeit
BUA	Breitbandultraschalldämpfung
BV/TV	Knochenvolumenanteil, trabekuläres Volumen
CEE	Konjugierte equine Östrogene; conjugated equine estrogens
CKD	Chronische Nierenerkrankung, chronic kidney disease
COPD	Chronisch obstruktive Lungenerkrankung, chronic obstructive pulmonary disease
CRP	C‑reaktives Protein
CSA	Committee of Scientific Advisors
CT	Computertomographie
Ct.Po	Kortikale Porosität
Ct.Th	Kortikalisdicke
CTX	C‑terminal crosslinking telopeptides of type I collagen
CTXA	Computertomographie-Röntgenabsorptiometrie, computed tomography X‑ray absorptiometry
DASH	Dietary Approaches to Stop Hypertension
DGE	Deutsche Gesellschaft für Ernährung
DGOU	Deutsche Gesellschaft für Orthopädie und Unfallchirurgie
DMPA	Depot-Medroxyprogesteron-Acetat
COPD	Chronisch obstruktive Atemwegserkrankung
DATA-Switch	Denosumab and teriparatide administration-Switch
DVO	Dachverband Osteologie
DXA	2‑Spektren-Röntgenabsorptiometrie, dual energy X‑ray absorptiometry
eGFR	Geschätzte glomeruläre Filtrationsrate, estimated glomerular filtration rate
EPOS	European Prospective Osteoporosis Study
ERα	Östrogenrezeptor alpha
ESCEO	European Society for Clinical and Economic Aspects of Osteoporosis, Osteoarthritis and Musculoskeletal Diseases
ESPEN	European Society for Parenteral and Enteral Nutrition
ESTES	European Society for Trauma and Emergency Surgery
EU	Europäische Union
FLS	Fracture Liaison Service
FN	Oberschenkelhals, femoral neck
FRAME	FRActure study in postmenopausal woMen with ostEoporosis
FRAX®	Fracture Risk Assessment Tool
FREEDOM	Fracture Reduction Evaluation of Denosumab in Osteoporosis Every 6 Months
FSH	Follikelstimulierendes Hormon
G-I‑N	Guideline International Network
Gamma-GT	Gamma-Glutamyl-Transferase
GC	Glukokortikoid
GIOP	Glukokortikoid-induzierte Osteoporose
GnRH	Gonadotropin-Releasing-Hormon
GÖG	Gesundheit Österreich GmbH
GRADE	Grading of Recommendations Assessment, Development, and Evaluation
HAL	Hüftachsenlänge
HIV	Humanes Immundefizienzvirus
HR	Hazard Ratio
HR-pQCT	High-resolution peripheral quantitative computed tomography
HRT	Hormonersatztherapie, hormone replacement therapy
HU	Hounsfield-Einheit, Hounsfield unit
IE	Internationale Einheit
ICD	International Classification of Diseases
IOF	International Osteoporosis Foundation
ISCD	International Society for Clinical Densitometry
kg	Kilogramm
KG	Körpergewicht
KMD	Knochenmineraldichte
LH	Luteinisierendes Hormon
LSC	Least significant change
LWS	Lendenwirbelsäule
μg	Mikrogramm
MDCT	Multidetektor-Computertomographie
mg	Milligramm
MGUS	Monoklonale Gammopathie unklarer Signifikanz
MHT	Menopausale Hormontherapie
MHz	Megahertz
ml	Milliliter
MMP-13	Matrix Metalloproteinase-13
MOF	Major osteoporotic fractures: hüftnahe Fraktur, klinisch vertebrale Fraktur, Unterarmfraktur, Humerusfraktur
MRONJ	Medikamenten-assoziierte Kiefer(osteo)nekrose, medication-related osteonecrosis of the jaw
MRT	Magnetresonanztomographie
mU/l	Milliunits pro Liter
ng	Nanogramm
NHANES	National Health and Nutrition Examination Survey
NICE	National Institute for Health and Care Excellence
NIH	National Institutes of Health
NOGG	National Osteoporosis Guideline Group (Quellleitlinie)
NSAR	Nichtsteroidale Antirheumatika
NVL	Nationale Versorgungsleitlinien
ÖGE	Österreichische Gesellschaft für Ernährung
ÖGKM	Österreichische Gesellschaft für Knochen- und Mineralstoffwechsel
ONJ	Kieferosteonekrose, osteonecrosis of the jaw
OR	Odds Ratio
ORIF	Operation der offenen Reposition und inneren Fixierung
OPG	Osteoprotegerin
POI	Premature Ovarian Insufficiency
PPI	Protonenpumpeninhibitoren
pQCT	Peripheral quantitative computed tomography
P1NP	N‑terminales Prokollagen Typ I
PTH	Parathormon
PTHrP	Parathormon-verwandtes Peptid, parathyoid hormone-related peptide
QCT	Quantitative Computertomographie
QM	Quantitativ
QUI	Quantitativer Ultraschallindex
QUS	Quantitativer Ultraschall
RA	Rheumatoide Arthritis
RANKL	Receptor activator of nuclear factor kappa B ligand
RCT	Randomized controlled trial
RR	Relatives Risiko
RRR	Relative Risikoreduktion
SD	Standardabweichung, standard deviation
SERM	Selektiver Östrogenrezeptormodulator
SIGN	Scottish Intercollegiate Guidelines Network
SOS	Speed of sound
SQ	Semiquantitativ
SSRI	Selektive Serotonin-Reuptake-Inhibitoren
STIRS	Short-Tau-Inversion-Recovery-Sequenz
STRUCTURE	STudy evaluating the effect of RomosozUmab Compared with Teriparatide in postmenopaUsal women with osteoporosis at high risk for fracture pReviously treated with bisphosphonatE therapy
TBS	Trabecular Bone Score
Tb.N	Durchschnittliche Anzahl der Trabekel
Tb.Sp	Durchschnittlicher Trabekelabstand
Tb.Th	Durchschnittliche Trabekeldicke
TSH	Thyreoidea-stimulierendes Hormon
VAT	Viszerales Fettgewebe, visceral adipose tissue
VERO	VERtebral Fracture Treatment Comparison in Osteoporotic Women
VFA	Vertebrales Frakturassessment
Vitamin D	25-OH-Vitamin D, 25-Hydroxy-Vitamin D, 25-Hydroxy-Vitamin D_3_, Calcifediol, 25-Hydroxycholecalciferol, 25(OH)D
VKA	Vitamin-K-Antagonisten
vKMD	Volumetrische Knochenmineraldichte
WHI	Women’s Health Initiative
WHO	Weltgesundheitsorganisation, World Health Organization

### Appendix B

#### Evidenzgrade und Empfehlungsstärken

Evidenzgrade und/oder Empfehlungsstärken wurden unverändert von den NOGG-Leitlinien [44] übernommen. Bei Quell- und Referenzleitlinien bzw. Positionspapieren, bei welchen die Evidenzgrade und/oder Empfehlungsstärken der SIGN-Leitlinien verwendet wurden, wurde diese gemäß der Tabelle B.1 in das System der NOGG übertragen.

**Tab. B.1:** *Übertragung von SIGN* [141] *zu NOGG* [44] *Graduierung der Evidenzgrade (Evidenz für Interventionsstudien)*. [Anmerkung: die Sortierung erfolgte gemäß den NOGG-Evidenzgraden]

SIGN	Definition	NOGG	Definition
1++	High-quality meta-analyses	*Ia*	Systematic review and meta-analysis of RCTs
1++	Systematic reviews of RCTs	*Ia*	Systematic review and meta-analysis of RCTs
1+	Well-conducted meta-analyses, systematic reviews with a low risk of bias	*Ia*	Systematic review and meta-analysis of RCTs
1−	Meta-analyses, systematic reviews with a high risk of bias	*Ia*	Systematic review and meta-analysis of RCTs
1++	RCTs with a very low risk of bias	*Ib*	Individual RCT(s) (with narrow confidence intervals)
1+	RCTs with a low risk of bias	*Ib*	Individual RCT(s) (with narrow confidence intervals)
2++	High-quality systematic reviews of case-control or cohort studies	*IIa*	Systematic review of at least one non-randomised controlled trial or well-designed cohort study
1−	RCTs with a high risk of bias	*IIb*	Individual cohort study or low-quality RCTs
2++	High-quality systematic reviews of case-control or cohort studies	*IIIa*	Systematic review of at least one case-controlled study
2++	High-quality case-control or cohort studies with a very low risk of confounding or bias and a high probability that the relationship is causal	*IIIb*	Individual case-control study
2+	Well-conducted case-control or cohort studies with a low risk of confounding or bias and a moderate probability that the relationship is causal	*IIIb*	Individual case-control study
2−	Case-control or cohort studies with a high risk of confounding or bias and a significant risk that the relationship is not causal	*IIIb*	Individual case-control study
3	Non-analytic studies, e. g. case reports, case series	*IV*	Expert committee reports or opinions and/or clinical experience of authorities, case series (and poor-quality cohort and case-control studies)
4	Expert opinion	*IV*	Expert committee reports or opinions and/or clinical experience of authorities, case series (and poor-quality cohort and case-control studies)

**Tab. B.2:** *NOGG-Evidenzgrade (Evidenz für die Gültigkeit von Kandidaten für Risikofaktoren)*. Anmerkung: SIGN kennt keine Differenzierung zwischen Evidenzgraden für Interventionsstudien und Kandidaten für Risikofaktoren

NOGG	Definition
*Ia*	Systematic reviews or meta-analysis of level I studies with a high degree of homogeneity
*Ib*	Systematic reviews or meta-analysis of level I studies with moderate or poor homogeneity
*Ic*	Level I studies (with appropriate populations and internal controls)
*IIa*	Systematic reviews or meta-analysis of level II studies
*IIb*	Level II studies (inappropriate population or lacking an internal control)
*IIIa*	Systematic reviews or meta-analysis of level III studies
*IIIb*	Case-control studies
*IV*	Evidence from expert committees without explicit critical scientific analysis or that based on physiology, basic research or first principles

**Tab. B.3:** *NOGG-Empfehlungsstärken – binäre Klassifizierung von Empfehlungen: „stark“ oder „bedingt“*. Anmerkung: SIGN verwendet bevorzugt „wünschenswerte/nicht wünschenswerte Effekte“ für starke Empfehlungen und „Werte/Präferenzen“ für bedingte Empfehlungen

The balance between desirable and undesirable effects	The larger the difference between the desirable and undesirable effects, the more likely a strong recommendation is warranted
The quality of evidence	The higher the quality of evidence, the more likely a strong recommendation is warranted
Values and preferences	The more variability/uncertainty in values and preferences the more likely a conditional recommendation is warranted
Costs (resource allocation)	The higher the costs of an intervention (i.e., the more resources consumed) the more likely a conditional recommendation is warranted

### Appendix C

#### Methodik der Leitlinienrecherche

##### *Leitliniensuche und Auswahl der Quellleitlinien*

Die Empfehlungen in diesen Leitlinien folgen dem aktuellen Stand der Wissenschaft mit Stand Mitte 2023. Folgend einer Empfehlung des Europarates 2001 zur Entwicklung einer Methodik für die Ausarbeitung von Leitlinien für optimale medizinische Praxis [390], wurden den vorliegenden Leitlinien verfügbare evidenzbasierte europäische Leitlinien zugrunde gelegt und deren Empfehlungen adaptiert.

Einschlusskriterien waren eine eindeutige Zuordnung der zugrunde liegenden Evidenz zur Empfehlung, Literatursuche mit nachvollziehbarer Systematik, Publikationsdatum ab 2016 bzw. noch gültig nach dem 1. Juni 2023, die Relevanz für den ambulanten Bereich (v. a. für den Bereich Allgemeinmedizin) sowie die Erstellung durch ein unabhängiges und anerkanntes Gremium aus Europa bzw. unter Beteiligung einer oder mehrerer europäischer Fachgesellschaften.

Die Leitliniensuche erfolgte am 6. Juni 2023 in der PubMed für den Zeitraum 1. Juni 2016 bis 1. Juni 2023 mit folgender Suchstrategie: ((osteoporosis[Title]) AND (Guideline[Title])) AND ((″2016/01/01″[Date - Publication]: ″2023/06/01″[Date - Publication])). Die Suche ergab 47 Resultate, davon wurden 36 Publikationen ausgeschlossen. Die Gründe für den Ausschluss waren: nicht-europäische Leitlinien (*n* = 15), Artikeltyp nicht Leitlinie (*n* = 14), Letters oder Kommentare (*n* = 5), Leitlinien aus anderen Therapiegebieten (Werner syndrome, *n* = 1). Die verbliebenen 11 Publikationen (s. Liste der 11 Leitlinien, die den Einschlusskriterien entsprachen) wurden gesichtet und dem ExpertInnengremium zur Verfügung gestellt.

Folgende weitere Websites und nationale sowie internationale Fachgesellschaften und Leitlinienanbieter wurden manuell nach gültigen und den Einschlusskriterien entsprechenden Leitlinien durchsucht: AWMF (Arbeitsgemeinschaft der Wissenschaftlichen Fachgesellschaften), G‑I‑N (Guideline International Network), NICE (National Institute for Health and Care Excellence), AHRQ (Agency for Healthcare Research and Quality, National Guideline Clearinghouse), SIGN (Scottish Intercollegiate Guidelines Network), NVL (nationale Versorgungsleitlinien), ISCD (International Society for Clinical Densitometry) und DVO (Dachverband Osteologie).

Zwei Quell- und Referenzleitlinien und 2 Positionspapiere wurden am 13. Juni 2023 vom Leitlinienkomitee als primäre Quellen festgelegt. Am 20.10.2023 wurde der American Society for Bone and Mineral Research (ASBMR) Primer, 9. Edition, durch Entscheid des Leitlinienkomitees als weitere Quellleitlinie definiert.

Es wurden insgesamt 5 Quellleitlinien für die Aktualisierung der Osteoporose-Leitlinien festgelegt:

- Leitlinie der Österreichischen Gesellschaft für Knochen- und Mineralstoffwechsel (ÖGKM) aus 2017 [42],
- Leitlinie der National Osteoporosis Guideline Group (NOGG) aus 2022 [44],
- Positionspapier für Erwachsene der International Society for Clinical Densitometry (ISCD) aus 2019 [148],
- Positionspapier zum Algorithmus für das Management von Patienten mit unterschiedlichem Frakturrisiko von Kanis et al. aus 2020 [140],
- ASBMR Primer on the Metabolic Bone Diseases and Disorders of Mineral Metabolism, 9th edition, aus 2018 [391].

Die S3-Leitlinie des DVO wurde nicht als Referenzleitlinie genutzt, da die Gültigkeit der bestehenden Fassung aus 2017 am 31. Dezember 2022 abgelaufen ist und die Aktualisierung bis zum Stichtag (1. Juni 2023) nicht publiziert war.

*Liste der 11 Leitlinien, die den Einschlusskriterien entsprachen:*

1. PMID: 33813622
  An assessment of intervention thresholds for very high fracture risk applied to the NOGG guidelines: A report for the National Osteoporosis Guideline Group (NOGG).
  Kanis JA, Johansson H, Harvey NC, Lorentzon M, Liu E, Vandenput L, McCloskey EV. Osteoporos Int. 2021 Oct; 32(10):1951–1960. 10.1007/s00198-021-05942-2.
2. PMID: 37101043
  [Diagnosis and management of patients with diabetes and co-existing osteoporosis (Update 2023): Common guideline of the Austrian Society for Bone and Mineral Research and the Austrian Diabetes Society].
  Muschitz C, Kautzky-Willer A, Winhofer Y, Rauner M, Haschka J, Cejka D, Wakolbinger-Habel R, Pietschmann P. Wien Klin Wochenschr. 2023 Jan; 135(Suppl 1):207–224. 10.1007/s00508-022-02118-8.
3. PMID: 34132916
  [Osteoporosis in pneumological diseases: Joint guideline of the Austrian Society for Bone and Mineral Research (ÖGKM) and the Austrian Society for Pneumology (ÖGP)].
  Muschitz C, Zwick RH, Haschka J, Dimai HP, Rauner M, Amrein K, Wakolbinger R, Jaksch P, Eber E, Pietschmann P. Wien Klin Wochenschr. 2021 Jun; 133(Suppl 4):155–173. 10.1007/s00508-021-01896-x.
4. PMID: 35384942
  Physical Therapist Management of Patients With Suspected or Confirmed Osteoporosis: A Clinical Practice Guideline From the Academy of Geriatric Physical Therapy.
  Hartley GW, Roach KE, Nithman RW, Betz SR, Lindsey C, Fuchs RK, Avin KG. J Geriatr Phys Ther. 2022 Apr–Jun 01; 44(2):80. 10.1519/JPT.0000000000000357.
5. PMID: 35676194
  Executive summary clinical practice guideline of postmenopausal, glucocorticoid-induced and male osteoporosis (2022 update). Spanish Society for Bone and Mineral Metabolism Investigation (SEIOMM).
  Riancho JA, Peris P, González-Macías J, Pérez-Castrillón JL; SEIOMM Osteoporosis Guidelines Writing Group. Rev Clin Esp (Barc). 2022 Aug-Sep; 222(7):432–439. 10.1016/j.rceng.2021.12.008.
6. PMID: 30980167
  [Diagnosis and management of patients with diabetes and co-existing osteoporosis (Update 2019): Common guideline of the Austrian Society for Bone and Mineral Research and the Austrian Diabetes Society].
  Muschitz C, Kautzky-Willer A, Rauner M, Winhöfer-Stöckl Y, Haschka J. Wien Klin Wochenschr. 2019 May; 131(Suppl 1):174–185. 10.1007/s00508-019-1462-0.
7. PMID: 35585444
  Correction: UK clinical guideline for the prevention and treatment of osteoporosis.
  Gregson CL, Armstrong DJ, Bowden J, Cooper C, Edwards J, Gittoes NJL, Harvey N, Kanis J, Leyland S, Low R, McCloskey E, Moss K, Parker J, Paskins Z, Poole K, Reid DM, Stone M, Thomson J, Vine N, Compston J. Arch Osteoporos. 2022 May 19; 17(1):80. 10.1007/s11657-022-01115-8.
8. PMID: 32068863
  Pharmacological Management of Osteoporosis in Postmenopausal Women: An Endocrine Society Guideline Update.
  Shoback D, Rosen CJ, Black DM, Cheung AM, Murad MH, Eastell R. J Clin Endocrinol Metab. 2020 Mar 1; 105(3):dgaa048. 10.1210/clinem/dgaa048.
9. PMID: 28425085
  UK clinical guideline for the prevention and treatment of osteoporosis.
  Compston J, Cooper A, Cooper C, Gittoes N, Gregson C, Harvey N, Hope S, Kanis JA, McCloskey EV, Poole KES, Reid DM, Selby P, Thompson F, Thurston A, Vine N; National Osteoporosis Guideline Group (NOGG). Arch Osteoporos. 2017 Dec; 12(1):43. 10.1007/s11657-017-0324-5.
10. PMID: 30907953
  Pharmacological Management of Osteoporosis in Postmenopausal Women: An Endocrine Society Clinical Practice Guideline.
  Eastell R, Rosen CJ, Black DM, Cheung AM, Murad MH, Shoback D. J Clin Endocrinol Metab. 2019 May 1; 104(5):1595–1622. 10.1210/jc.2019-00221.
11. PMID: 35378630
  UK clinical guideline for the prevention and treatment of osteoporosis.
  Gregson CL, Armstrong DJ, Bowden J, Cooper C, Edwards J, Gittoes NJL, Harvey N, Kanis J, Leyland S, Low R, McCloskey E, Moss K, Parker J, Paskins Z, Poole K, Reid DM, Stone M, Thomson J, Vine N, Compston J. Arch Osteoporos. 2022 Apr 5; 17(1):58. 10.1007/s11657-022-01061-5.

### Appendix D

#### Beschreibung des Aktualisierungsprozesses

Die Aktualisierung der Osteoporose-Leitlinien von Arznei & Vernunft aus 2017 [42] wurde unter der Leitung von Ao. Univ.-Prof. Dr. Hans Peter Dimai, Medizinische Universität Graz, und PD Dr. Christian Muschitz, Medizinische Universität Wien, geplant und durchgeführt. Die Planung und Durchführung der Aktualisierung der Leitlinien erfolgte entsprechend den Kriterien des Appraisal of Guidelines for Research and Evaluation (AGREE) Trust zur Erstellung evidenzbasierter Leitlinien (https://www.agreetrust.org) [392].

Auf Basis der Osteoporose-Leitlinie aus 2017 und wichtiger Entwicklungen seit deren Publikation wurden die Inhalte der aktuellen Leitlinien definiert. Für die einzelnen Kapitel wurden Expertinnen und Experten für die jeweiligen Themengebiete als Leitautorinnen und -autoren bzw. Koautorinnen und -autoren gewonnen (s. Tab. D.1). Die Inhalte und Autorinnen und Autoren wurden während einer virtuellen Sitzung am 13. Juni 2023 konsensual beschlossen. Den Autorinnen und Autoren wurden die Referenzleitlinien sowie ein Musterkapitel zur Verfügung gestellt. Alle Inhalte und offenen Fragen wurden diskutiert und konsensual beschlossen. Nach der Zusammenführung aller Kapitel und einheitlicher Formatierung erfolgten eine finale Durchsicht und schriftliche Freigabe durch alle Expertinnen und Experten. In weiterer Folge wurde das finale Dokument 2 externen Gutachtern vorgelegt, deren Kommentare wurden umgesetzt und die Umsetzung wurde schriftlich dokumentiert.

Das Projektmanagement und Medical Writing erfolgte durch Dr. med. Uli Kiesswetter. Medical Writing und Koordination der Publikation wurde durch Dr. Margit Hemetsberger erbracht. Das Endlektorat erfolgte durch Mag. Heinz Javorsky.

**Tab. D.1:** Mitglieder des Leitlinienkomitees

Name	Titel	Affiliation
Karin Amrein	PD Dr. MSc	Medizinische Universität Graz
Rosemarie Bauer	Dr.	Dachverband der Sozialversicherungsträger
Daniel Cejka	PD Dr.	Ordensklinikum Linz
Hans Peter Dimai	Univ.-Prof. Dr.	Medizinische Universität Graz
Astrid Fahrleitner-Pammer	Univ.-Prof. Dr.	Medizinische Universität Graz
Rudolf Wolfgang Gasser	Univ.-Prof. Dr.	Medizinische Universität Innsbruck
Reinhard Gruber	Univ.-Prof. Dr.	Medizinische Universität Wien
Judith Haschka	OÄ Dr.	Hanusch Krankenhaus Wien
Timothy Hasenöhrl	Univ.-Ass. Mag. Dr.	Medizinische Universität Wien
Franz Kainberger	Univ.-Prof. Dr.	Medizinische Universität Wien
Katharina Kerschan-Schindl	Univ.-Prof. Dr.	Medizinische Universität Wien
Uli Kiesswetter	Dr.	Perchtoldsdorf (selbstständig)
Roland Kocijan	PD Dr.	Hanusch Krankenhaus Wien
Jürgen König	Univ.-Prof. Dr.	Universität Wien
Norbert Kroißenbrunner	Dr.	Turnau (Steiermark)
Ulrike Kuchler	PD DDr.	Medizinische Universität Wien
Christian Muschitz	PD Dr.	healthPi Medical Center Wien; Medizinische Universität Wien
Christine Oberforcher	–	Osteoporose Selbsthilfegruppe Österreich
Johannes Ott	Univ.-Prof. Dr.	Medizinische Universität Wien
Georg Pfeiler	Univ.-Prof. Dr.	Medizinische Universität Wien
Peter Pietschmann	Univ.-Prof. Dr.	Medizinische Universität Wien
Paul Puchwein	PD Dr.	Medizinische Universität Graz
Alexander Schmidt-Ilsinger	Mag. MSc	Österr. Apothekerkammer
Ralf Harun Zwick	Dr.	Therme Wien Med

## Interessenkonflikt

H.P. Dimai erhielt eine Unterstützung für das vorliegende Manuskript von der Österreichischen Gesellschaft für Knochen- und Mineralstoffwechsel und von der Medizinischen Universität Graz; Beratungshonorare, Sprecherhonorare und Reisekostenzuschüsse von Amgen, Braincon, Daiichi-Sankyo, Eli Lilly, Gedeon Richter, Medtronic, Merck Sharp & Dohme, Novartis, Nycomed, Servier, Sona-Pharm, UCB. Er hält Führungspositionen im Committee of Scientific Advisors (CAS) sowie in der International Osteoporosis Foundation (IOS). C. Muschitz erhielt Sprecherhonorare von Eli Lilly Austria, Amgen Austria, Gedeon Richter Austria, Stada Austria, UCB Austria, Austria Medical Chamber; Zahlungen für Tätigkeiten als Sachverständiger von Eli Lilly Austria, Amgen Austria, Gedeon Richter Austria; Teilnahme an Beratungsgremien von Eli Lilly Austria. K. Amrein erhielt Förderungen von Fresenius Kabi; Beratungshonorare, Zahlungen für Tätigkeiten als Sachverständige sowie Teilnahme an Beratungsgremien von Ascendis Pharma; Sprecherhonorare von Wörwag Pharma, Vifor Pharma, Gebro Pharma, Fresenius, Novo Nordisk, Amgen. D. Cejka erhielt Beratungshonorare von CSL-Vifor, Gedeon Richter, Stada; Sprecherhonorare von CSL-Vifor, Takeda, UCB. Er hält Führungspositionen in der Österreichischen Gesellschaft für Knochen- und Mineralstoffwechsel (ÖGKM; management board member) und in der Chronic Kidney Disease-Mineral and Bone Disorder (CKD-MBD) working group of the European Renal Association (ERA; vice chair). R.W. Gasser erhielt Reiseunterstützungen von der Österreichischen Gesellschaft für Knochen- und Mineralstoffwechsel (ÖGKM) und Reiseunterstützung und ein Sprecherhonorar von OSTAK Osteologie Akademie GmbH. Er hält eine Führungsposition bei der ÖGKM. J. Haschka erhielt Beratungshonorare von Eli Lilly, Janssen; Sprecherhonorare von Amgen, UCB, Kyowa Kirin, Alexion, Gedeon Richter, Eli Lilly; Reisekostenzuschüsse von UCB, Stada, Janssen. K. Kerschan-Schindl erhielt Beratungshonorare von Gedeon Richter Austria GmbH; Sprecherhonorare von Amgen GmbH, Roche Austria, Eli Lilly GmbH; Reisekostenunterstützung von Amgen GmbH, Roche Austria, Eli Lilly GmbH, Merck Sharp & Dohme GmbH; sonstiges Equipment und Materialien von Sanovi Aventis, Servier Austria. R. Kocijan erhielt Sprecherhonorare von Alexion, Amgen, Eli Lilly, Gedeon Richter, Kyowa Kirin, Novartis, Stada, Roche, UCB. Reisekostenunterstützung von Amgen, Gedeon Richter, Stada, Roche, UCB. Beratungshonorare von Alexion, UCB, Gedeon Richter, Roche. U. Kuchler hält Führungspositionen bei der Österreichischen Gesellschaft für Implantologie (ÖGI) und dem Österreichischen Führungsgremium des International Team of Implantology (ITI). J. Ott erhielt Sprecherhonorare von Lenus Pharma, Exeltis, Gedeon Richter, Gynial. G. Pfeiler erhielt Förderungen von Pfizer, AstraZeneca, Roche, Accord; Beratungshonorare, Sprecherhonorare und Reisekostenunterstützung von Pfizer, Roche, MSD, Accord, Eli Lilly, Merck, Gilead, Seagen, Novartis, Daiichi, AstraZeneca. P. Pietschmann erhielt Förderungen von Amgen GmbH, Meda Pharma/Mylan GmbH, UCB Pharma, Fresenius Kabi Austria GmbH, Shire Austria GmbH, Springer-Verlag GmbH und war als wissenschaftlicher Berater für TAmiRNA tätig. Er hält Führungspositionen bei der European Calcified Tissue Society (ECTS; President) und der Österreichischen Gesellschaft für Knochen- und Mineralstoffwechsel (ÖGKM). P. Puchwein erhielt Förderungen (Fortbildungszuschüsse und Unterstützungszahlungen an die Abteilung) von Johnson & Johnson DS, USA; Lizenzgebühren von Hofer Medical Solutions, Fürstenfeld, Österreich; Beratungshonorare von I.T.S. Lassnitzhöhe, Österreich; Sprecherhonorare für Alphamed Fischer, Österreich, Fresenius Kabi, Deutschland, Amgen GmbH, Deutschland. Er hat eine Führungsfunktion (Chair Section Education) bei der Österreichischen Gesellschaft für Orthopädie und Traumatologie (öGoUT) inne. A. Fahrleitner-Pammer erhielt Förderungen von Amgen; Beratungshonorare von Medimaps; Sprecherhonorare von Amgen, Stada, Gedeon Richter, Gebro, Gerot Lannacher, Teva Ratiopharm, UCB, Fresenius; Reisekostenunterstützung von Gedeon Richter, UCB, Medimaps. Sie hält Aktien/Aktienoptionen an Skin Screener. R. Bauer, R. Gruber, T. Hasenöhrl, F. Kainberger, J. König, N. Kroißenbrunner, C. Oberforcher, A. Schmidt-Ilsinger und R.H. Zwick geben an, dass kein Interessenkonflikt besteht.
